# Fundamentals and Design‐Led Synthesis of Emulsion‐Templated Porous Materials for Environmental Applications

**DOI:** 10.1002/advs.202102540

**Published:** 2021-09-22

**Authors:** Muhammad Ahmad Mudassir, Hafiz Zohaib Aslam, Tariq Mahmood Ansari, Haifei Zhang, Irshad Hussain

**Affiliations:** ^1^ Department of Chemistry & Chemical Engineering SBA School of Science & Engineering (SBASSE) Lahore University of Management Sciences (LUMS) Lahore 54792 Pakistan; ^2^ Department of Chemistry Khwaja Fareed University of Engineering & Information Technology (KFUEIT) Rahim Yar Khan 64200 Pakistan; ^3^ Institute of Chemical Sciences Bahauddin Zakariya University (BZU) Multan 60800 Pakistan; ^4^ Department of Chemistry University of Liverpool Oxford Street Liverpool L69 7ZD UK

**Keywords:** emulsion templating, environmental remediation, porous materials, sensing, water/air treatment

## Abstract

Emulsion templating is at the forefront of producing a wide array of porous materials that offers interconnected porous structure, easy permeability, homogeneous flow‐through, high diffusion rates, convective mass transfer, and direct accessibility to interact with atoms/ions/molecules throughout the exterior and interior of the bulk. These interesting features together with easily available ingredients, facile preparation methods, flexible pore‐size tuning protocols, controlled surface modification strategies, good physicochemical and dimensional stability, lightweight, convenient processing and subsequent recovery, superior pollutants remediation/monitoring performance, and decent recyclability underscore the benchmark potential of the emulsion‐templated porous materials in large‐scale practical environmental applications. To this end, many research breakthroughs in emulsion templating technique witnessed by the recent achievements have been widely unfolded and currently being extensively explored to address many of the environmental challenges. Taking into account the burgeoning progress of the emulsion‐templated porous materials in the environmental field, this review article provides a conceptual overview of emulsions and emulsion templating technique, sums up the general procedures to design and fabricate many state‐of‐the‐art emulsion‐templated porous materials, and presents a critical overview of their marked momentum in adsorption, separation, disinfection, catalysis/degradation, capture, and sensing of the inorganic, organic and biological contaminants in water and air.

## Graphical abstract

Understanding the structure‐property relationship of materials‐by‐design is very useful to optimize materials predictive functions. Porous materials, especially those prepared via emulsion templating, offer great promise in ubiquitous environmental remediation applications and beyond owing to their interconnected porous structure, enhanced permeability, tunable chemistry, homogeneous flow‐through, high diffusion rates, convective mass transfer, and thorough accessibility for direct interaction with the pollutants for subsequent removal.

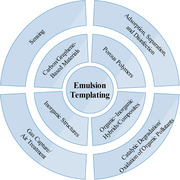

## 1 Introduction

### 1.1 Demand of Porous Materials for Environmental Applications

Despite the amazing merits of the development in science and technology for the advancement of human society, it is also associated with severe environmental concerns pertaining to population growth‐oriented rapid industrialization and rising energy demand. The pollution of water, air, and land occurring at an alarming rate has cascaded the dreadful effects on the ecological balance of this planet. The environmental imbalance is in turn plaguing the quality of public health by causing contagious diseases particularly in children of low‐ and middle‐income countries.^[^ ^1^ ^]^ To provide effective solution of such mounting environmental problems, many researchers and scientists have put forward meticulous efforts to prepare a wide array of materials including non‐conventional materials (e.g., zeolites, mineral clays, sawdust, chitosan (CS), waste biomatter, etc.) metal and metal oxides (e.g., gold, silver (Ag), iron, iron oxide, titanium oxide, etc.), polymers/dendrimers, organic–inorganic composites/hybrids, and carbon nanomaterials (e.g., commercial activated carbon, graphitic biochar) with tailored structures to treat different types of pollutants by adsorption, degradation (radical and non‐radical), biological treatment, and filtration by membranes and nanofibrous media, etc. Notwithstanding, the lack of selectivity, low pollutant remediation performance, complicated operation and handling, secondary contamination by the leaching of nano/microsized particles, production of huge quantity of toxic sludge, expensive regeneration processes etc. circumvent their use in large‐scale practical applications. It is thus a matter of substantial interest to develop novel materials with potential applications in environmental remediation.^[^ ^2^, ^3^, ^4^, ^5^ ^]^

Among a wide variety of conventional functional materials, the nanostructured zero‐dimensional (0D), one‐dimensional (1D), two‐dimensional (2D), and three‐dimensional (3D) materials are more attractive as compared to their bulk counterparts due to their greater proportion of surface atoms and higher surface energy. 0D nanomaterials have all three dimensions in the nanometric range with a size range of ≈1–100 nm such as heterogeneous particles arrays, nanoclusters, uniform particle arrays (quantum dots), core–shell quantum dots, hollow spheres, and nanolenses etc. The 1D (e.g., nanorods, nanotubes, nanowires, nanofibers, nanofilaments, nanobelts, nanoribbons, hierarchical nanostructures, etc.) and 2D (junctions, branched structures, nanoprisms, nanowalls, nanodisks, nanofilms, nanolayers, nanocoatings, nanowalls, nanosheets, nanoflakes, nanoplates, etc.) nanomaterials have one and two dimensions outside of the nanoscale regime, respectively. 3D nanomaterials including nanocoils, nanocones, nanoballs (dendritic structures), nanoflowers, and nanopillers are not confined to the nanoscale in any dimension. Although the 0D nanomaterials exhibit unique physicochemical properties because of their versatility in spatial structure and morphology, 1D and 2D nanomaterials tend to be more advantageous in general, particularly owing to their 1D/2D configuration‐oriented transport pathways. However, assembling 2D building units/blocks into hierarchical 3D architectures is drawing greater scientific and technological attention due to their higher specific surface areas with a large number of active/anchoring sites to interact with ions, atoms, and molecules throughout the surface and interior of the bulk. For example, the commonly used 3D structures of 2D materials are networks, papers, monoliths, foams, scaffolds, sponges, hydrogels, aerogels, and frameworks. The interconnected porosity in all dimensions accounts for the effective mass transfer and makes such materials practically worthwhile, especially for the development of various water treatment technologies.^[^ ^3^, ^6^, ^7^, ^8^ ^]^

The level, size, shape, volume, orientation, and surface chemistry of pores can be tuned or designed in porous materials in order to perform the anticipated functions. Based on the classification of pore sizes by the International Union of Pure and Applied Chemistry (IUPAC), porous materials can be classified as microporous (pore size ≤2 nm), mesoporous (pore size 2–50 nm), and macroporous (pore size >50 nm).^[^ ^4^, ^9^ ^]^ Each class of these porous materials offers a characteristic mechanism for the adsorption and transportation of liquids and gases in their matrix depending on their morphology, porous structure, and surface chemistry. Liquids and gases show a viscous flow and molecular diffusion process in macroporous materials, surface diffusion and capillary transport in mesoporous materials, and activated transport behavior in microporous materials. The mass transport limitations experienced by various classes of porous materials can be alleviated by the use of hierarchically porous materials composed of one, two, or more levels of interconnected micro‐, meso‐, and macropores.^[^ ^4^, ^9^, ^10^ ^]^ The hierarchy of materials on porosity spans over multiple length scales ranging from micropores to mesopores and macropores. The interconnectivity and regularity of pores at bimodal (micro‐microporous, micro‐mesoporous, micro‐macroporous, meso‐mesoporous, meso‐macroporous, macro‐macroporous) and multimodal (micro‐meso‐macroporous, meso‐meso‐macroporous) levels, high surface area, large accessible space, low density, and outstanding volume change accommodation facilitate easier mass diffusion and transport. These hierarchically structured porous materials have attracted a great deal of attention in adsorption, photocatalysis, separation, and sensing nowadays. Therefore the “Materials‐Properties‐by‐Design” is a meaningful concept to understand structure–property relationship and to achieve the optimized predictive functions for ubiquitous environmental applications.^[^ ^11^ ^]^

Controlled self‐assembly and synthesis via molecular design and controlled chemistry have been intensively investigated for the fabrication of micropores and mesopores with desirable shape, size, adaptability, and surface chemistry.^[^ ^7^, ^12^, ^13^, ^14^ ^]^ Notwithstanding, the pores are usually created via the use of templates (both hard and soft templates) or phase separation.^[^ ^6^, ^12^, ^15^, ^16^, ^17^ ^]^ Among various methods, foam, emulsion, and foamed templating have gained wider interest to prepare multifunctional porous materials. Each of these liquid templating routes offers access to different structures. For instance, the foam templating is an energy‐saving method because it uses air as the templating phase that is not required to be removed. Nonetheless, the difficulty to foam non‐polar liquids and the strong influence of gravity on the properties of foams impede the applicability of this method. Nevertheless, the provision of 3D visualization and a greater control over the influence of gravity during generation may be achieved by matching viscosities, densities, or optical indices using emulsion templating route. The emulsion templating approach provides access to wider ranges of pore sizes (100 nm–2 mm) and porosities (64–97%), whereby the liquids (hydrophilic vs hydrophobic) may be easily reversed by appropriately choosing an emulsifier.^[^ ^18^, ^19^, ^20^ ^]^

In emulsion templating, the removal of the dispersed phase is a cost‐inefficient practice notwithstanding; the presence of dispersed phase is now being demanded for many new emerging applications. Interestingly, the combination of the templating methods with various emulsification and foaming techniques develops a tool box to fabricate tailor‐made materials with characteristic property profiles for anticipated long‐term and practical applications. For instance, a foamed emulsion or foamulsion combines the merits of foam as well as emulsion and may be polymerized without losing template structure. This foamed emulsion templating may also be used to foam even hydrophobic monomers like pure styrene (St) by first generating a styrene‐in‐water (St/W) emulsion followed by foaming. Furthermore, this approach also favors the formation of second‐scale hierarchically porous structures.^[^ ^18^, ^20^, ^21^ ^]^

**Figure 1:**
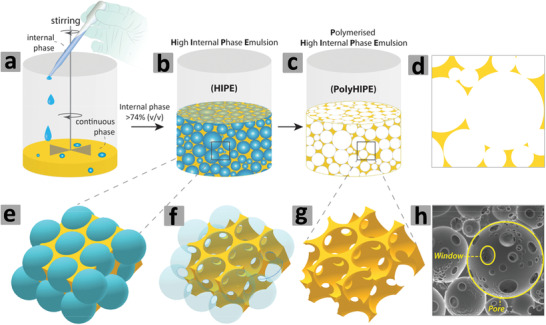
Steps involved in preparation of polymerized high internal phase emulsion (polyHIPE). a,b) The dropwise addition of the dispersed phase into the continuous phase to obtain HIPE, c) polymerization of the HIPE, d) 2D projection of polyHIPE, e–g) the pores and windows formation, and h) Scanning electron microscopy (SEM) image of the polyHIPE. Reproduced under the terms of the Creative Commons Attribution License (CC BY).^[^ ^30^ ^]^ Copyright 2020, Aldemir Dikici and Claeyssens, published by Frontiers.

Emulsion templating is one of the templating methods, which has been highly effective in fabricating a wide range of macroporous materials with highly interconnected porosity.^[^ ^16^, ^22^, ^23^, ^24^, ^25^, ^26^, ^27^, ^28^, ^29^ ^]^ The method employs small liquid droplets in an emulsion as templates that can be subsequently removed by washing or vacuum drying without the need to use harsh conditions such as chemical etching or high temperature calcination. The volume percentage of droplet phase in an emulsion can be adjusted to systematically vary the porosity of the prepared materials. Particularly, high internal phase emulsions (HIPEs, with the volume percentage of dispersed droplet phase >74.05%) have been widely used as templates to produce highly interconnected porous materials with ultralow density (**Figure** **1**).

The use of HIPEs as templates in combination with the other synthesis and templating methods is unique in preparing hierarchical porous materials, thereby providing both high number of active sites and enhanced mass transport.^[^ ^25^, ^26^, ^27^, ^28^, ^29^ ^]^ In the last two decades, most of the emulsion‐templated porous materials, mainly polymers, have been prepared by employing HIPEs as templates. Accordingly, excellent reviews have been published, but most of them focus on the preparation and applications of emulsion‐templated porous polymers.^[^ ^22^, ^23^, ^24^, ^25^, ^26^, ^27^, ^28^, ^29^ ^]^

This comprehensive review covers the fundamentals and basics of emulsion templating and the progress in the full spectrum of emulsion‐templated porous polymers, composites/hybrids, and derivatives for their applications in environmental remediation. This review summarizes the merits as well as demerits of various methods used to prepare advanced emulsion‐templated porous materials and also discusses the influence of their different designs or shapes (membranes, rods, fibers, microspheres, macrobeads, monoliths, etc.) on their performance in task‐specific environmental applications and industrial worth.

### 1.2 Conceptual Overview

#### 1.2.1 Emulsion and Emulsification

**Figure 2:**
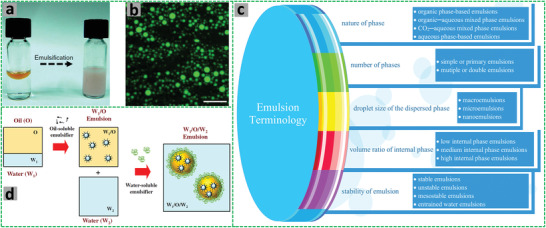
a) A digital photograph shows emulsification of the silicone oil (20%) and water (80%). b) A fluorescence micrograph of the O/W emulsion, scale bar 10 µm. Reproduced under the terms of the Creative Commons CC BY license.^[^ ^33^ ^]^ Copyright 2017, The Authors(s), published by Springer Nature. c) Schematic illustration shows the classification of emulsions. d) The scheme shows how a water‐in‐oil‐in‐water (W/O/W) emulsion is prepared. Reproduced with permission.^[^ ^35^ ^]^ Copyright 2018, Elsevier.

Emulsions are usually regarded as heterogeneous liquid colloidal dispersions formed by mixing one liquid phase (referred as dispersed or internal phase, in droplets form) into another immiscible continuous liquid phase (termed as continuous or external phase), usually in the presence of a surfactant. Most of the emulsions consist of an aqueous phase and an oil (hydrocarbon) phase, but emulsions composed of two non‐aqueous (organic) phases or two aqueous phases have also been introduced.^[^ ^23^, ^24^, ^31^, ^32^ ^]^ Emulsions are usually formed by the dropwise addition of one phase into another under stirring (or homogenization, vortex)^[^ ^32^ ^]^ but can also be simply prepared by shaking and mixing of two immiscible liquids (**Figure** **2**a,b).^[^ ^33^ ^]^ Another method to instantly form emulsions by shaking involves the use of water‐soluble porous polymer.^[^ ^34^ ^]^ This porous polymer is immersed in an oil (or organic solvent) and then taken out and placed in water. By simply shaking, the porous polymer scaffold is dissolved and the absorbed oil is released into water to form oil‐in‐water (O/W) emulsions.^[^ ^34^ ^]^

The process of emulsification can be retarded by several factors such as flocculation, coalescence, ripening, inversion, creaming, and sedimentation that may destabilize the emulsions, leading to phase separation.^[^ ^32^, ^35^, ^36^ ^]^ Flocculation is determined by the magnitude of attractive force versus repulsive force, wherein droplets get together to form loosely bound aggregates. The stability of liquid film existing between the aggregated droplets determines the occurrence of the coalescence process. Ripening is also a subtype of coalescence and arises when large droplets tend to grow at the cost of smaller ones. The process of phase inversion may appear by the exchange between the dispersed phase and the continuous phase. However, the effect of buoyancy force may lead to the migration of the dispersed phase to the top (when the density of the droplet phase is lower than that of the continuous phase) or its sinking to the bottom (when the droplet phase is denser than the continuous phase) of the emulsion and cause creaming and sedimentation, respectively.^[^ ^32^, ^35^, ^36^ ^]^ A surfactant is a substance containing hydrophobic and hydrophilic groups, also known as a stabilizer or an emulsifier, usually added to prepare an emulsion.^[^ ^31^, ^32^ ^]^ To further improve emulsion stability, additional surfactants (known as co‐surfactant or co‐stabilizer) can also be introduced into the emulsions. These surfactant molecules are located at the interface of the two liquid phases and form a protective film to minimize the chances of emulsion destabilization while impeding droplet coalescence pertaining to the reduced interfacial tension of small droplets.^[^ ^32^, ^35^, ^36^ ^]^

#### 1.2.2 Emulsion Terminology

Emulsions are classified in a number of different ways depending upon various factors such as the nature of the dispersed phase, droplet size of the dispersed phase, ratios of the phase volumes, and stability of the emulsion as shown in Figure 2c.

##### Nature of Phases

An emulsion can be classified by the nature of its phases. Most emulsions encompass immiscible phases of water and an organic phase while some emulsions consist of both organic phases (non‐aqueous) with a difference in their polarities, including amides (formamide or dimethylformamide), glycols (ethylene glycols), poly‐alcohols (glycerol), lower alcohols (methanol), alkylated sulfoxides [dimethyl sulfoxide (DMSO),] and acetonitrile or their solutions.^[^ ^31^ ^]^

When emulsions containing an organic phase are used in producing highly porous materials for a variety of applications, the complete removal of the organic phase requires the use of more organic solvents for washing or an energy‐consuming vacuum process. Both approaches are not environmentally friendly. Furthermore, the complete removal of such biologically incompatible solvents is a highly challenging task, which undermines their use in biomedical applications. In order to solve this problem, supercritical carbon dioxide has been used as a nontoxic, nonflammable, and naturally abundant solvent to prepare CO_2_‐in‐water (C/W) emulsions for the preparation of highly porous materials.^[^ ^37^, ^38^, ^39^ ^]^

The C/W emulsions have been employed as templates to produce highly porous hydrophilic polymers without using organic solvents, although involving the use of expensive and non‐biodegradable fluorinated surfactants and high pressures (>300 bar). Non‐fluorinated surfactants for C/W emulsions have since been developed,^[^ ^37^, ^38^, ^39^ ^]^ but the synthesis and scale‐up of such surfactants are still highly challenging. Furthermore, the use of high pressure requirement is still unavoidable, which requires high energy consumption and increases the capital cost of the equipment and associated operations.

Ionic liquids (ILs) are widely regarded as green solvents mainly due to their non‐volatile and non‐flammable properties. Their thermal stability is also favored for chemical processing and thermally initiated reactions and polymerizations.^[^ ^40^ ^]^ ILs have been used to form emulsions, replacing either water or oil. As such, IL‐in‐oil, IL‐in‐water, IL‐in‐IL, and CO_2_‐in‐IL emulsions have been formed depending on the diverse properties of ILs available.^[^ ^41^, ^42^ ^]^ Although ILs have been widely investigated for various applications as “green solvents”,^[^ ^40^, ^43^ ^]^ there have been concerns about their environmental and potential health and safety impacts.^[^ ^44^ ^]^ With a multi‐criteria decision analysis on greenness, ILs may be ranked between recommended polar solvents and problematic solvents.^[^ ^44^ ^]^

In order to form emulsions with totally benign solvents, water‐in‐water (W/W) emulsions have been introduced. They are prepared by mixing two incompatible aqueous phases with hydrophilic polymers dissolved in water above certain threshold concentrations. The equilibrium between the two co‐existing aqueous phases is achieved to maintain the relative emulsion stability.^[^ ^45^ ^]^ These W/W emulsions are highly demanded in cosmetics, pharmaceuticals, and foods industries but encounter stability problems. Moreover, their stabilization by using molecular surfactants is very challenging due to their ultralow water–water interfacial tensions (1 to 1000 µN m^−1^) and relatively large interfacial thickness. In early studies, the macroscopic phase separation was avoided by gelation of one or even both phases. However, recent studies have successfully used ultrathin plate‐like colloidal particles to prepare stable W/W emulsions.^[^ ^46^ ^]^

These particles form a layer to block much of the water–water interface that in turn reduces the free energy and thus stabilizes the resultant W/W emulsions. This concept of stabilizing interfaces with particles of different shapes and sizes is known as the “Pickering effect” or “Pickering stabilization”,^[^ ^47^ ^]^ which has also been used to stabilize O/W, water‐in‐oil (W/O), and non‐aqueous emulsions.^[^ ^48^, ^49^, ^50^, ^51^ ^]^ Different types of particles including metal–organic framework (MOF) nanoparticles (NPs) have been used as stabilizers for Pickering emulsions.^[^ ^51^ ^]^ For instance, the W/W emulsions have been stabilized with nanoplates and nanorods in the form of cellulose nanocrystals.^[^ ^48^, ^49^ ^]^ This approach is well‐suited for sustainable applications because the cellulose materials are nontoxic, biodegradable, and abundantly available.^[^ ^48^ ^]^ However, since hydrophilic polymers are dissolved in both aqueous phases, W/W emulsions are rarely used as templates to fabricate porous materials.

##### Number of Phases

Emulsions can be made up of two or more phases of similar or varying nature. The emulsions that are composed of two phases are known as simple or primary emulsions. W/W, O/W, W/O, and C/W emulsions are the examples of simple/primary emulsions, whereas the emulsion comprising more than two phases are known as multiple or double emulsions, or emulsion in another emulsion.^[^ ^35^, ^36^, ^52^ ^]^ The types of double emulsions include W/O/W and oil‐in‐water‐in‐oil (O/W/O) emulsions. The W/O/W double emulsions (Figure 2d) have substantial advantages over simple O/W emulsions for microencapsulation applications, as each dispersed water droplet is separated from the continuous aqueous phase in multiple emulsions by a layer of an oil phase. However, the typical double emulsions are thermodynamically unstable systems owing to their relatively large droplet size.^[^ ^35^, ^36^, ^52^, ^53^, ^54^ ^]^

Usually, the preparation of the double emulsions involves a two‐step emulsification process, wherein a primary emulsion is formed under high shear conditions followed by the subsequent dispersion of the primary emulsion into another incompatible phase under a relatively mild shear force in order to avoid rupture of the internal water droplets.^[^ ^35^, ^36^, ^52^ ^]^ An example of how a W/O/W emulsion can be prepared is illustrated in Figure 2d.^[^ ^35^ ^]^ Similarly, O/W/O emulsions can be formed by preparing an O/W emulsion first, which is then dispersed into a second oil phase. Double emulsions are generally polydisperse and poorly controlled in structure because each emulsification step leads to a wider distribution of droplets in size. However, monodisperse double emulsions with better control of droplet size may be produced by membrane emulsification,^[^ ^54^, ^55^ ^]^ microcapillary device,^[^ ^54^, ^55^, ^56^ ^]^ or microfluidics in general.^[^ ^57^ ^]^

##### Droplet Size of the Dispersed Phase

**Figure 3:**
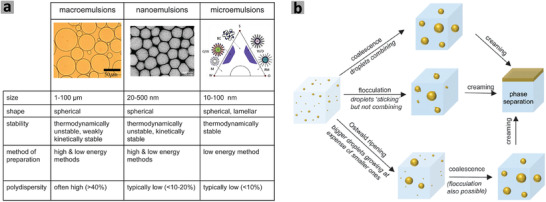
a) Classification of macroemulsion, nanoemulsion, and microemulsions based on size, shape, stability, preparation method, and polydispersity. b) A schematic representation of emulsion destabilization mechanisms. Reproduced under the terms of the Creative Commons CC BY‐NC 3.0 license.^[^ ^58^ ^]^ Copyright 2005, The Author(s), published by Royal Society of Chemistry.

On the basis of the droplet size of the dispersed phase, emulsions may be classified as macro, micro, and nanoemulsions (**Figure** **3**a).^[^ ^58^ ^]^ When one mentions “an emulsion”, it is normally referred to as a macroemulsion. Microemulsions are transparent and thermodynamically stable containing the micelles swollen with the internal phase.^[^ ^32^, ^59^ ^]^ Nanoemulsions, also called as miniemulsions or submicron emulsions, are formed with the droplet sizes in the nanosized regime (up to 500 nm), between the droplet sizes of microemulsions and macroemulsions. Both macroemulsions and nanoemulsions can be prepared using low and high energy input while microemulsions are usually formed by low energy methods (Figure 3a). High energy methods usually include high pressure homogenization and ultrasonication.^[^ ^32^, ^58^ ^]^ However, preparation of nanoemulsions or microemulsions usually requires larger amount of surfactants and lower volume percentage of the internal phase.^[^ ^31^, ^32^, ^58^, ^59^, ^60^ ^]^ Although multiple macroemulsions are common, studies about multiple nanoemulsions are emerging for the applications when the micronsize droplets in multiple emulsions are too large to be used.^[^ ^61^ ^]^

##### Volume Percentage of Internal Phase

On the basis of volume ratio or percentage between internal phase and the whole emulsion, emulsions can be classified as low internal phase emulsions (LIPEs), medium internal phase emulsions (MIPEs), and HIPEs. It is generally accepted that the LIPEs, MIPEs, and HIPEs contain internal phase volumes of less than 30%, 30–74%, and >74.05% (compared to the volume of the emulsions), respectively.^[^ ^23^, ^24^, ^25^, ^26^, ^27^, ^28^, ^29^ ^]^ Due to their high viscosity, HIPEs are also known as concentrated emulsions, gel‐like emulsions, or gel emulsions.^[^ ^28^, ^62^, ^63^ ^]^ HIPEs may contain more than 83% internal phase or even over 90% internal phase where the droplets are highly packed and monodisperse, showing polyhedral shapes.^[^ ^24^, ^26^, ^64^ ^]^

##### Stability of Emulsion

Due to the large droplet size (exceeding 1000 nm), macroemulsions are thermodynamically unstable and phase separation (as characterized by creaming, flocculation, coalescence, and Ostwald ripening) occurs readily if stored for a longer period of time.^[^ ^32^ ^]^ Emulsions are destabilized or phase separated via different mechanisms such as creaming, flocculation, coalescence, and Ostwald ripening (Figure 3b).^[^ ^58^, ^59^ ^]^ Big droplets grow bigger by consuming smaller droplets via Ostwald ripening, which leads to higher degree of coalescence or flocculation. In the subsequent process, large droplets either rise due to buoyancy (droplets lighter than the continuous phase) or settle down by sedimentation (the density of droplets is greater than that of the continuous phase), resulting in total phase separation.^[^ ^58^ ^]^

Emulsions are thermodynamically unstable but can be kinetically stable. The stability of emulsions can be improved by reducing the rate of Ostwald ripening, flocculation, and coalescence by adjusting/controlling emulsion formulation and preparation conditions. The emulsifiers (type and concentration), additives, solubility and partitioning, ionic strength, interfacial charge, volume fraction and size of droplets, and temperature are taken into account in this regard.^[^ ^35^, ^36^, ^58^ ^]^ The usage of mixing emulsifiers has been reviewed to improve emulsion stability and its impact on emulsion functionality has also been assessed.^[^ ^35^ ^]^

Half‐life, appearance, and rheological properties of an emulsion may indicate the stability of an emulsion. For instance, a study on the formation process of W/O emulsions classified the emulsions as stable emulsions, unstable emulsions, mesostable emulsions, and entrained‐water emulsions.^[^ ^65^ ^]^ In that case, the stable emulsions could be reddish to brown in color containing about 80% water for W/O emulsions from the first day to a week with significant elasticity. The viscosity of the emulsions at a shear rate of one reciprocal second was three orders of magnitude greater than that of the oil. Such emulsions maintained their stability for at least 4 weeks under ambient conditions.^[^ ^65^ ^]^

#### 1.2.3 Emulsion Templating

Emulsion templating is a technique that uses an emulsion as a template (porogen), more specifically, using droplets as templates while monomers (or other precursors) are dissolved in the continuous phase. After polymerization (or other curing/solidifying process) in the continuous phase, the emulsion‐templated structure is locked. The subsequent removal of solvent from the dispersed phase produces its porous replica i.e., an emulsion‐templated porous material.^[^ ^22^, ^24^, ^26^ ^]^

The synthesis of emulsion‐templated porous materials is a multistep process that involves formulation of the continuous (polymer) and dispersed (internal) phases, emulsification, structuring and solidification of the emulsions, and post‐processing (incorporation of co‐monomers, post‐functionalization, purification, etc.). The types and composition of emulsion ingredients (i.e., monomers, crosslinkers, solvents, stabilizers, initiators, etc.) together with experimental/operational parameters/conditions (e.g., temperature, mixing efficiency/method, etc.) significantly influence the properties of the resultant emulsion‐templated porous materials.^[^ ^29^, ^30^ ^]^

The choice of monomers is directly associated with the predetermined properties of the emulsion‐templated porous structures. For instance, the hydrophobic (degradable and non‐degradable) polymers tend to fabricate W/O polyHIPEs, whereas hydrophilic polymers are used to prepare O/W polyHIPEs. Similarly, crosslinkers with at least two reactive ends are used to connect primary polymer chains through intermolecular linkages. Furthermore, the external crosslinker may increase the extent of crosslinking that improves the stiffness of emulsion‐templated porous materials.^[^ ^30^, ^66^ ^]^

Though the high viscosity enhances the kinetic stability of emulsion, it is still preferred to use the lowest possible amount of the continuous phase enough to achieve stable emulsion. Alternatively, the continuous phase is heated and/or diluted by using diluting/porogenic solvents in order to generate highly porous scaffolds. Likewise, the addition of some salts like sodium sulfate, calcium chloride, sodium chloride, and potassium iodide also enhances the stability of emulsions. The stabilizing agents are believed to reduce the interfacial tension, thereby stabilizing the oil–water interface.^[^ ^30^, ^63^, ^67^, ^68^ ^]^

Since the surfactant (amphiphilic compound) is tension active molecule or mesogen having water‐soluble head and oil‐soluble tail, it acts as a barrier between two phases, reduces interfacial tension, and thus stabilizes the emulsion. The choice and concentration of surfactants have a greater impact on emulsion stabilization. For example, water‐soluble surfactants form O/W emulsions and oil‐soluble surfactants may stabilize W/O emulsion. On the other side, the higher surfactant concentration generates smaller pores with uniform size distribution. However, the surfactant is usually intended not to react with the monomer and is subsequently removed after polymerization.^[^ ^30^, ^68^, ^69^, ^70^ ^]^

Apart from using surfactants, solid particles/NPs are also used to achieve emulsion stability. The stabilization of such surfactant‐free (Pickering) emulsions basically depends upon wettability of the particles by the water and oil phases. In this connection, the water‐wetted particles tend to form O/W emulsions, whereas oil‐wetted particles are suitable for the preparation of W/O emulsions. Likewise, the concentration of particles also affects the pore size of the resultant emulsion‐templated porous materials.^[^ ^30^, ^71^ ^–^ ^73^, ^74^ ^]^ Furthermore, the initiators that react with the monomers to form intermediate compounds, which in turn link with the other monomer units to generate the polymer chains. The locus of initiation may also affect the interconnectivity and structure of pores in emulsion‐templated materials. The choice of initiator has an impact on the morphology of emulsion‐templated porous materials, whereas its concentration may influence the curing time and mechanical properties.^[^ ^29^, ^30^, ^71^, ^75^ ^]^

Temperature is inversely related to the viscosity of the oil phase as well as stability of the emulsion. In this regard, moderate temperatures are favorable to create relatively more stable emulsions.^[^ ^30^, ^76^, ^77^, ^78^ ^]^ Also the mode (i.e., over‐head stirrer, magnetic stirrer, mechanical shaking, speed mixer, vortexer, homogenizer, and manual shaking) and efficiency of mixing influence the internal phase volume. In this way, the higher speed of mixing results in smaller emulsions and thus the pore sizes. Furthermore, the way (syringe pump or dropping funnel) of adding inner phase into the continuous phase affects the emulsion stability and the pore size distribution.^[^ ^30^, ^69^, ^72^, ^76^, ^77^, ^78^, ^79^, ^80^ ^]^

Typically, the emulsion templating is considered to be different from emulsion polymerization/microencapsulation techniques. Normally, the emulsion templating method involves W/O systems, whereas emulsion polymerization/microencapsulation that uses significantly lower internal phase volumes generally occur in O/W emulsions. The reactions in emulsion templating generally take place within the external phase, whereas the reactions normally begins with monomers solubilized within surfactant micelles and polymerization continues within the monomer‐swollen polymer NPs in the case of emulsion polymerization and at the oil–water interface in the microencapsulation systems.^[^ ^29^, ^81^ ^]^

Emulsion templating is used to produce monoliths, whereas the emulsion polymerization and microencapsulation methods yield particles. However, the synthesis of a wide variety of emulsion‐templated porous polymers usually involves polymerization of emulsions. The free radical (e.g., thermal, photo‐initiated, and redox‐initiated) polymerization is a conventional and widely used mechanism, which is, however, limited to monomers with reactive double bonds such as styrenics and (meth)acrylates under specific reaction conditions and generally produces porous polymers with non‐homogeneous network. To circumvent network homogeneity problem, the controlled radical polymerizations such as atom‐transfer radical polymerization (e.g., reverse atom‐transfer radical polymerization and activators generated by electron transfer), and reversible addition–fragmentation chain‐transfer polymerization have been employed. Likewise, the step‐growth polymerization, Diels–Alder polymerization, ring‐opening polymerization, and the thiol–ene/thiol–yne click reactions have also been used for the same purpose.^[^ ^29^ ^]^

The emulsion structure may also be locked by freezing the emulsion, where a polymer is usually dissolved in the continuous phase. The subsequent freeze drying process removes the solvents from both the continuous phase and droplet phase and generates highly interconnected emulsion‐templated porous structures.^[^ ^82^, ^83^ ^]^ This method is often applied to O/W emulsions. The advantages include: 1) the emulsions do not have to be very stable because they can be frozen immediately after emulsion preparation; 2) the ice templating in the continuous phase introduces additional porosity in the material. Ice templating utilizes frozen solvent crystals as templates for a wide range of porous materials, organic/drug NPs, and nanofibers, where the solvents can be water or organic solvents such as cyclohexane, DMSO, chloroform, xylene, or compressed CO_2_.^[^ ^82^, ^83^, ^84^, ^85^, ^86^ ^]^

**Figure 4:**
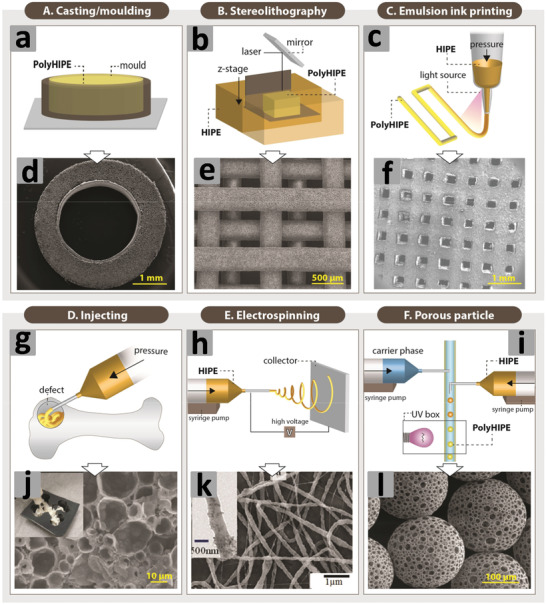
a–c,g–i) Setups of different fabrication routes. d–f),j–l) SEM images of emulsion templated scaffolds. Reproduced under the terms of the Creative Commons Attribution License (CC BY).^[^ ^30^ ^]^ Copyright 2020, Aldemir Dikici and Claeyssens, published by Frontiers.

Earlier emulsion templating is actually a casting technique that usually involves the preparation of emulsions and their subsequent placement in suitable molds or spreading on substrates followed by polymerization for design‐led synthesis of emulsion‐templated porous materials (**Figure** **4**a,d). This facile approach does not require any additional technical equipment for scaling‐up the process and is used to prepare scaffolds of different sizes and shapes. Recent work on emulsion templating encompasses polymer engineering and mechanics (i.e., stereolithography, emulsion ink printing, injecting, electrospinning, microfluidics, etc.) for the synthesis of porous beads, rods, fibers, monoliths, membranes, and complex shapes with high reproducibility and greater control on exterior architecture.^[^ ^29^, ^30^ ^]^

The stereolithography is a laser‐based approach used to selectively polymerize photo‐sensitive liquid resins in a layer‐by‐layer manner (Figure 4b,e). The emulsion ink printing method involves the preparation of emulsion ink followed by its filling into the printing head reservoir and printing in the designed 3D shape (Figure 4c,f). The stereolithography and emulsion ink printing routes combine emulsion templating and additive manufacturing techniques to enable the development of well‐defined multiscale porous scaffolds. However, unlike stereolithography, emulsion ink printing is useful in the formation of heterogeneous structures.^[^ ^30^, ^87^ ^]^

The injecting method involves injectable emulsions and polymerization at physiological temperatures without using toxic solvents. This route can be used to fabricate defect matching scaffolds, but is limited only to materials that can be used to prepare injectable emulsions (Figure 4g,j). On the other hand, electrospinning is a versatile approach used to fabricate fibers of different materials with varying diameters (Figure 4h,k). The controlled stirred‐tank reactor is simple and practically worthwhile system, though it produces polydisperse particles. However, the microfluidic systems are quite effective in producing monodisperse porous particles with high control over their size (Figure 4i,l).^[^ ^30^, ^88^, ^89^ ^]^

The porous polymers prepared by using HIPEs as templates are known as polyHIPEs. Recently, HIPEs have been increasingly used to prepare hierarchically porous functional polymers, with the ability to tune the shape and morphology of the pores in the polyHIPEs.^[^ ^22^, ^23^, ^24^, ^25^, ^26^, ^27^, ^28^, ^29^ ^]^ The pores in polyHIPEs are usually spherical and interconnected voids. The spherical pores are termed as “cells” in open‐cell (solid) foams and more preferably as “voids” for the substrates in cell culturing in order to avoid ambiguity among cells (pores) of substrate and the cells of microorganisms (see Figure 1h). The average void (pore) size of polyHIPEs is usually in the range of 1–100 µm.^[^ ^24^, ^25^, ^26^ ^]^ The interconnected holes between the voids in polyHIPEs are termed as “windows” (see Figure 1h). Due to their inherent macroporosity (pore volumes ≈10 cm^3^ g^−1^ or higher), the polyHIPEs exhibit low surface areas (10–50 m^2^ g^−1^), which may, however, be increased (300–700 m^2^ g^−1^) through generating secondary structures of hierarchical and interconnected pores by adjusting the ratios of crosslinkers and introducing inert porogens.^[^ ^24^, ^25^, ^26^, ^90^, ^91^ ^]^

To enhance suitability for various applications, the nature of polyHIPEs is modified by introducing functional components (usually monomers) into the HIPE and postsynthetic functionalization. The postsynthetic treatment that involves surface‐specific modifications is principally used for surface enrichment and producing changes in hydrophilicity–hydrophobicity, whereas the etching, hypercrosslinking, surface activation, and porogen removal mainly generates mesoporosity and/or microporosity while retaining their inherent macroporosity.^[^ ^29^, ^91^, ^92^, ^93^ ^]^ These hypercrosslinked polymers exhibit multiple pore size distributions (i.e., micropores, mesopores, and emulsion‐templated macropores) and high surface areas. However, such postsynthetic modifications may destroy the porous structure and deleteriously affect the mechanical properties of the original polyHIPEs. Furthermore, the practical adoption of such types of modification requires to minimize the number of synthesis steps.^[^ ^29^, ^91^, ^92^, ^93^ ^]^

Of the common methods such as the direct synthesis, block copolymer self‐assembly, and direct templating, freeze drying is the simplest way used for the preparation of aligned porous materials but this approach cannot easily tune the size and structure of the pores.^[^ ^94^ ^]^ Nonetheless, the direct templating approach—emulsion templating is a flexible, easily controlled, and versatile methodology for the design‐led production of a wide variety of tailor‐made porous materials including porous organic polymers,^[^ ^24^, ^25^, ^26^ ^]^ organic–inorganic composites/hybrids,^[^ ^25^, ^29^, ^95^ ^]^ inorganic structures,^[^ ^22^, ^96^ ^]^ and carbonaceous/graphene materials. Different formats of the emulsion‐templated materials including membranes, films, rods, fibers, beads, and monoliths have been prepared with highly interconnected porosity and high specific surface area.^[^ ^24^, ^25^, ^26^, ^29^, ^82^, ^97^ ^]^

## 2 Emulsion‐Templated Porous Materials

Emulsion‐templated porous materials are categorized into four main types in this review. The materials with size‐selective pores/apertures, favorable geometry, required surface chemistry, and good chemical and thermal stability can be designed and fabricated by controlling the chemistry of the monomers, the polymer matrix, and the nature of inorganic constituents, and postsynthetic modification.

### 2.1 Emulsion‐Templated Porous Polymers

Emulsion‐templated porous polymers are light‐weight materials having unique thermal, mechanical, and acoustic properties associated with their characteristic features. The dimensions, size distribution, shape (spherical or polyhedral), hierarchy, and interconnectivity of pores as well as surface areas, structural gradients, design (shape and morphology), and the size of the porous polymeric materials are of paramount importance for application point of view.^[^ ^20^ ^]^

The size and combination of pores govern transportation of liquid/gas throughout the bulk materials. The microporous materials show activated transport behavior with a sluggish mass transfer particularly for bulky molecules. The capillary transport in mesoporous materials is governed by Knudsen and surface diffusion mechanism, wherein the molecules usually show collision with the pore walls. The macroporous materials enable viscous flow and molecular diffusion, whereby the intermolecular collision regulates the momentum and energy exchange with minimum diffusion berries to enhance mass transport.^[^ ^98^ ^]^

The hierarchy of material on porosity ensures efficient (fast and broad) distribution of liquid/gas throughout the bulk material with minimum diffusion/transport resistance. The interconnectivity of pores is of greater interest because the closed‐cell structures are effective in encapsulation applications and open‐cell structures are useful in absorption, adsorption, tissue engineering, controlled release, reaction supports, and membranes. The higher surface area lead to an increased number of active sites, thereby increasing surface reactivity and thus improving application performance.^[^ ^4^, ^29^, ^98^, ^99^ ^]^

The structure of the porous polymer materials is related to the mechanical and physical properties of polymers. The presence of porosity gradient with lamination often leads to stress localization, thereby promoting crack propagation. However, the materials possessing a porosity gradient without laminated structures exhibit compact outer skin and porosity.^[^ ^20^, ^100^ ^]^ Nano/microscale materials offer impressive performance though; their practical applications are limited by the drawbacks associated with their processing and recovery, whereas the millimeter size eases the handling and subsequent separation of macrosized materials during the application tests, thereby making such materials practically worthwhile.^[^ ^98^, ^101^, ^102^, ^103^ ^]^

The precise control over all these synthetic parameters is tricky and can be successfully achieved by trial‐and‐error experiments and a lot of development is still in process for further advancement. In this connection, the emulsion templating produces materials of high porosities (64–97%) with a wide pore size range (100 nm–2 mm). The pore interconnectivity mainly depends on the concentration of surfactant, whereas the pore shape can be influenced by the locus of initiation of polymerization. However,the greater extent of porosity may be attained by initiating the polymerization within the continuous polymer phase.^[^ ^20^, ^104^ ^]^

Both hydrophobic (non‐degradable and degradable) and hydrophilic emulsion‐templated porous polymers can be prepared from W/O emulsions or O/W emulsions. Earlier, the St monomer has been used to prepare non‐degradable emulsion‐templated substrates. Later, St‐DVB (styrene‐divinylbenzene) polyHIPEs were patented as cell growth media, which has attracted great interest. Likewise, 2‐ethylhexyl acrylate (EHA)‐isobornyl acrylate (IBOA), a blend of acrylate‐based monomers, is another example of commonly reported non‐degradable hydrophobic material. The poly(*ε*‐caprolactone) (PCL) is known to be the earliest biodegradable polymer but the emulsification of its HIPE is a cumbersome process on account of its high viscosity. The PCL and its copolymer polylactic‐*co*‐glycolic acid have extensively been used in tissue engineering. Similarly, fumarate‐based polyHIPEs such as poly(propylene fumarate) is unsaturated linear polyester that has been used in orthopedic implants, whereas thiol(ene/yne)‐based degradable polyHIPEs have received attention in various applications.^[^ ^30^, ^68^, ^76^, ^79^, ^105^ ^]^

Naturally derived polymers have been used to prepare hydrophilic polyHIPEs from O/W emulsions. For instance, gelatin derived from collagen of skin, bones, or tendon of animals is a natural biopolymer commonly used in tissue engineering applications. Likewise, an amphiphilic gelatin‐graft‐poly(*N*‐isopropylacrylamide) (PNIPAM) has been developed by grafting gelatin with PNIPAM. Another biopolymer alginate derived from seaweed has also been used to prepare polyHIPEs. Furthermore, dextran and pullulan methacrylate hydrophilic polymers have also been reported.^[^ ^30^, ^106^ ^]^

The following section describes the preparation methods in more detail to discuss the synthetic conditions and the porosity of non‐aqueous, W/O, O/W, C/W, W/W, O/W/O, and W/O/W emulsion‐templated hydrophobic (non‐degradable and degradable) and hydrophilic porous polymer.

#### 2.1.1 St‐Based Hydrophobic Porous Polymers

A high percentage of hydrophobic emulsion‐templated polymers have been prepared from W/O HIPEs using St monomer, divinylbenzene (DVB) crosslinker, and other comonomers, particularly at the earlier stage of polyHIPE researches.^[^ ^24^, ^25^, ^26^, ^27^, ^28^, ^29^ ^]^ The W/O emulsions are usually formed by dropwise addition of water phase into the continuous organic phase made up of monomers, crosslinkers, initiators [(e.g., 2,2′‐azobis(2‐methylpropionitrile (AIBN)], surfactants [e.g., sorbitan monooleate (Span 80)], and/or organic solvents.

Porous poly(styrene‐*co*‐divinylbenzene) [P(St‐*co*‐DVB)] monoliths with open cellular structure were prepared by using 1,2‐dichloroethane (DCE) as a solvent, AIBN as an initiator, and Span 80 as an emulsifier through W/O emulsion polymerization.^[^ ^107^ ^]^ Cation exchange groups were subsequently quantitatively introduced onto the monoliths by swelling them into the solution of dichloromethane (DCM). Chlorosulfonic acid was then added dropwise to the swollen monoliths followed by the addition of glacial acetic acid. Such monolithic resins showed advantages over the conventional ion‐exchange resins because of their easy column packing, high exchange rates, and smaller ion exchange band length.^[^ ^107^ ^]^ In another study, P(St‐*co*‐DVB) polyHIPEs were synthesized by first preparing and slowly adding the ferric hydroxide hydrosol to the oil phase containing St, DVB, Span 80, Poloxamer 188, AIBN, and toluene under constant stirring followed by polymerization at 65 °C. The resulting polymers were soaked in HCl solution at 65 °C to remove Fe(OH)_3_, washed with water and ethanol, and subsequently dried. Afterward, polyHIPEs were embedded with polyethylenimine (PEI) to obtain PEI‐impregnated polyHIPEs, which showed superior performance for CO_2_ capture from flue gas and selective uptake of CO_2_ over N_2_.^[^ ^108^ ^]^

**Figure 5:**
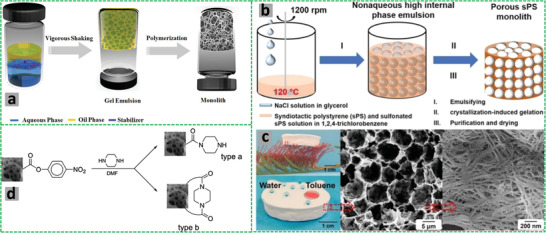
a) Schematic illustration of the preparation of porous polymeric monoliths via gel‐emulsion templating. Reproduced with permission.^[^ ^110^ ^]^ Copyright 2019, Wiley‐VCH. b) Schematic representation of preparing hierarchically porous emulsion‐templated syndiotactic polystyrene (sPS) monoliths via crystallization‐induced gelation; c) the sPS monolith exhibits low density, superhydrophobicity, and nanofibrous porous structure. Adapted with permission.^[^ ^112^ ^]^ Copyright 2019, American Chemical Society. d) Modification of poly(styrene‐*co*‐4‐nitrophenylacrylate) [P(St‐*co*‐NPA)] PolyHIPEs with piperazine. Reproduced with permission.^[^ ^117^ ^]^ Copyright 2007, Elsevier.

The W/O HIPEs formed can be highly viscous, milky, and gel‐like. These emulsions are stable and remain attached to the vial wall when the vial containing the emulsions is placed upside down (**Figure** **5**a).^[^ ^109^, ^110^ ^]^ A gel‐like emulsion containing St/AIBN/DVB was polymerized and the resulting monolith was extracted and washed with alcohol and dried in air, leading to the formation of porous polystyrene (PS) with ultralow density.^[^ ^109^ ^]^ In a further study, additional crosslinkers such as ethylene glycol dimethacrylate (EGDMA) and trihydroxymethylpropyl trimethylacrylate were introduced to enhance mechanical stability of the formed porous monoliths with an average pore diameter of 14.7 µm. These hydrophobic materials exhibited a low density of 0.035 g cm^−3^ and thermal conductivity of 0.032 W m^−1^ k^−1^.^[^ ^110^ ^]^

Crosslinked PS‐based stationary phase in capillary has also been prepared. The organic phase was made up of St, DVB, and Span 80. The aqueous solution was formed by dissolving potassium persulfate (KPS) (0.2 wt%) and calcium chloride (1 wt%) and then added to the organic phase under stirring. The resulting cream‐like emulsion was imbibed into a capillary with applied external pressure of 2.5 bars. The open ends of the emulsion‐loaded capillary were sealed and the capillary was immersed into a hot water bath (60 °C) for 48 h followed by washing with water and acetonitrile using a liquid chromatographic pump.^[^ ^111^ ^]^

A series of emulsion‐templated porous sPS monoliths with the pore wall consisting of nanofibers (diameters 20–100 nm) were prepared from non‐aqueous HIPEs via a crystallization‐induced gelation approach.^[^ ^112^ ^]^ Sulfonated sPS (2 w/v%, acting as stabilizer) and sPS (8 w/v%) were dissolved in 1,2,4‐trichlorobenzene (TCB) at 160 °C under N_2_. Glycerol solution containing 1 w/v% NaCl was emulsified in TCB solution to form non‐aqueous HIPEs at 120 °C. The non‐aqueous HIPEs were cooled to room temperature (RT) and solidified. Porous sPS monoliths were generated after removing TCB and glycerol by exchange with water followed by freeze drying and then vacuum drying at 80 °C. The sPS monoliths exhibited very low density and hierarchical porosity with voids of around 10 µm, throats of 1–2 µm, and nanofibrous walls (Figure 5b,c). The macropores and mesopores between nanofibers rendered the monoliths with high specific surface areas of up to 420 m^2^ g^−1^. These superhydrophobic and oleophilic porous monoliths (with water contact angles over 150°) were robust with a compressive strain of 70% and Young's moduli ranging from 157.7–2638.0 kPa.^[^ ^112^ ^]^

poly(styrene‐*co*‐vinylbenzyl chloride‐*co*‐divinylbenzene) [P(St‐*co*‐VBC‐*co*‐DVB)] lightly crosslinked polyHIPEs were prepared from the HIPEs containing St, DVB, 4‐vinylbenzyl chloride (VBC), AIBN, and Span 80 in the oil phase and aqueous phase containing dissolved potassium sulfate.^[^ ^113^ ^]^ The HIPEs were formed by the dropwise addition of the aqueous phase into the oil phase via a simple PTFE overhead paddle‐assisted stirring. The high pore volume, interconnected macropores, and adjustable content of VBC in P(St‐*co*‐VBC‐*co*‐DVB) polyHIPEs facilitated rapid swelling, uptake of liquids, and fast immobilization of chemical warfare agents.^[^ ^113^ ^]^

P(St‐*co*‐VBC‐*co*‐DVB) polyHIPEs were functionalized by surface graft polymerization of 4‐vinylpyridine (4VP), which were subsequently employed for the separation of heavy metal ions (iron and plutonium ions) with improved kinetics.^[^ ^114^ ^]^ Recently, poly(4‐vinylpyridine) (P4VP)‐grafted polyHIPE foams were prepared by incorporating dormant nitroxide as a co‐monomer into the foam backbone followed by the surface decoration with a brush of P4VP. The impregnated co‐monomers acted as the nitroxide‐mediated polymerization sites to allow the growth of P4VP chains.^[^ ^115^ ^]^ Due to its high permeability and connective mass transfer, P(St‐*co*‐VBC‐*co*‐DVB) polyHIPE monolithic columns were easily functionalized with tris(2‐amino‐ethyl)amine, diethanolamine, and 4‐bromophenylboronic acid via a simple flow‐through method. The polymerization of such reactive monomers was highly beneficial to support a scavenger or a reagent.^[^ ^116^ ^]^

HIPE‐templated amino‐functionalized poly(styrene‐*co*‐methyl methacrylate) P(St‐*co*‐MMA) monolith have been prepared by copolymerization of St and MMA (methyl methacrylate) with DVB as crosslinker followed by functionalization with ethylenediamine (EDA). The resultant porous functionalized monolith possessed voids and windows of 3.0–7.4 and 1.1–2.4 µm, respectively. Such porous monoliths also exhibited excellent thermal stability up to 323 °C.^[^ ^118^ ^]^

Piperazine‐functionalized P(St‐*co*‐NPA)‐based polyHIPEs were also fabricated. The continuous organic phase of W/O HIPEs comprised 4‐nitrophenylacrylate, DVB, Span 80, Span 85, and chlorobenzene (CB). The aqueous phase containing potassium peroxydisulfate and calcium chloride was added to the oil phase dropwise under constant stirring (350 rpm for 1.5 h) with an overhead stirrer. The polyHIPEs were formed by curing the HIPEs in a mold at 70 °C for 2 days followed by functionalization with piperazine, which was indeed necessary for the removal of atrazine from water (Figure 5d).^[^ ^117^ ^]^

**Figure 6:**
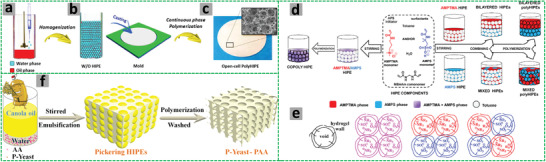
a–c) Schematic demonstration of the P(St‐*co*‐DEAEMA) membrane preparation. Reproduced with permission.^[^ ^119^ ^]^ Copyright 2017, American Chemical Society. Schematic representation for the preparation of polyampholyte polyHIPEs from oppositely charged monomers AMPTMA and 2‐acrylamido‐2‐methyl‐1‐propanesulfonic acid (AMPS): d) Three ways of mixing the monomers; e) surface groups in the emulsion‐templated pores. Reproduced with permission.^[^ ^147^ ^]^ Copyright 2020, Elsevier. f) Schematic illustration for the preparation of superporous pretreated yeast poly(acrylic acid) (PAA) monoliths. Adapted with permission.^[^ ^155^ ^]^ Copyright 2019, Elsevier.

Highly porous poly[styrene‐2‐(diethylamino)ethyl methacrylate] P(St‐DEAEMA) membranes were prepared through a facile and inexpensive W/O emulsion polymerization approach.^[^ ^119^ ^]^ The oil phase was prepared by adding St, 2‐(diethylamino)ethyl methacrylate (DEAEMA), DVB, and Span 80 into a plastic (polypropylene) container. The degassed aqueous phase containing KPS and calcium chloride was gradually added to the oil phase under stirring to form the HIPE, which was cast in a homemade Teflon membrane model and cured at 70 °C for 48 h for complete polymerization. The white solid polyHIPE membrane was washed in Soxhlet apparatus with ethanol and subsequently dried (**Figure** **6**a–c). The presence of DEAEMA group ensured switching wettability between hydrophobicity and hydrophilicity after CO_2_ treatment by bubbling into water with the immersed P(St‐DEAEMA) membranes.^[^ ^119^ ^]^

A reversible addition–fragmentation chain transfer process by reversible deactivation radical polymerization was adopted to synthesize a block copolymer poly(oligo(ethylene glycol) methyl ether methacrylate)‐*block*‐polystyrene (POEGMA‐*b*‐PS). This amphiphilic block copolymer was further used as a stabilizer for W/O gel emulsion (with 93 v/v% water and St, EGDMA, and AIBN as the oil phase) without using any co‐stabilizer. After polymerization, washing and drying, the resulting white solid showed pore sizes in the range of 5–50 µm, a minimum density of 0.08 g cm^−3^, very high resistance to water, and a water contact angle of ≈120°.^[^ ^120^ ^]^

St/EHA/DVB‐based emulsion‐templated tubular membranes were prepared by molding a very viscous white HIPE into thin layers between flat plates, separated by poly(ethylene terephthalate) films, and subsequently polymerized. Afterward, the resulting membranes were impregnated with ionophores, graphite particles, electron mediators, and enzymes. This made the functionalized membrane highly useful for sensor applications.^[^ ^121^ ^]^

Emulsion‐templated porous fluorinated poly[2‐(perfluorohexyl)ethyl methacrylate‐styrene‐divinylbenzene] [P(PEM‐St‐DVB)] material was prepared by the dropwise addition of aqueous phase (containing KPS and NaCl) to the oil phase (mixture of St, DVB, PEM (2‐(perfluorohexyl)ethyl methacrylate), and Span 80 under continuous stirring. Due to the low interface energy of perfluorinated compounds, PEM was introduced in order to enhance surface hydrophobicity of the final material. The resulting emulsion was placed into a sealed polypropylene tube and polymerized at 60 °C for 24 h. The as‐prepared polyHIPE was washed with ethanol by Soxhlet extraction for 48 h and dried. This fluorinated polyHIPE exhibited a highly open porous structure with superhydrophobicity (a water contact angle of 151°) and oleophilicity.^[^ ^122^ ^]^

#### 2.1.2 Other Hydrophobic Porous Polymers

Acrylic‐based poly(isodecyl acrylate‐*co*‐divinylbenzene) [P(IDA‐*co*‐DVB)] monoliths have been fabricated via an in situ polymerization of the continuous phase consisting of isodecyl acrylate (IDA) and DVB. The polyHIPE monoliths exhibited well‐defined and open‐cell structure with interconnected spherical voids (8.9 ± 2.7 µm) and windows (2.0 ± 0.9 µm) and a surface area of 5.44 m^2^ g^−1^. Notably, this method offered a flexible and easily controlled alternative for preparing monolithic columns whose availability previously remained limited in the market because of the complications in their preparation.^[^ ^123^ ^]^

P(VBC‐DVB) adsorbents were prepared using the HIPEs composed of an oil phase containing VBC‐DVB (35/65) monomers, chloroethylbenzene as a porogen, and surfactant mixture [(cetyltrimethylammonium bromide (CTAB)/SPAN80/sodium dodecylbenzenesulfonate (SDBS)] as emulsifier, and 90 v/v % water.^[^ ^124^ ^]^ The resulting emulsion‐templated porous polymers were then functionalized with different diamines including EDA, piperazine, aminopiperidine, and imidazole in order to improve CO_2_ adsorption capacity. The surface areas of as‐prepared polyHIPEs, EDA‐functionalized polyHIPEs, piperazine‐functionalized polyHIPEs, aminopiperidine‐functionalized polyHIPEs, and imidazole‐functionalized polyHIPEs came out to be 150, 48, 32, 36, and 133 m^2^ g^−1^.^[^ ^124^ ^]^

A gelation method via ionic interaction has been reported to prepare macroporous hydrophobic polymer sponges. The porous monoliths were fabricated from sulfonated polystyrene‐*block*‐poly(ethylene‐*ran*‐butylene)‐*block*‐polystyrene (SPS‐*b*‐PE‐*r*‐Bt‐*b*‐PS) and tetra‐functional poly(amidoamine) (PAMAM) dendrimers based on the ionic interaction within W/O HIPEs. The prepared sponges exhibited typical emulsion‐templated interconnected macroporous structures posturing voids of 1–20 µm with densities of 0.08–0.1 g cm^−3^.^[^ ^125^ ^]^ Emulsion‐templated xerogels were prepared by freeze‐drying of the HIPE organogels obtained from the polypropylenimine (PPI) dendrimer and the block ionomer SPS‐*b*‐PE‐*r*‐Bt‐*b*‐PS. These xerogels displayed high porosity and low density (0.108 g cm^−3^) due to the high volume fraction of the dispersed phase in the HIPE organogels.^[^ ^126^ ^]^

Highly porous emulsion‐templated poly(arylene ethynylene) (PAE)‐based foams were prepared from the HIPEs composed of the continuous toluene phase containing dissolved monomers (1,3,5‐triethynylbenzene and 1,4‐diiodobenzene, 2,5‐dimethoxy‐1,4‐diiodobenzene or 2,6‐diiodo‐4‐nitrophenol), surfactant Span 80, tetrakis(triphenylphosphine) palladium, and CuI and the dispersed aqueous phase containing K_2_CO_3_. A Sonogashira cross‐coupling polymerization in the continuous phase led to the formation of *π*‐conjugated PAE‐based polyHIPEs with fine tuning of optical bandgaps (1.70–2.35 eV). Such PAE‐ based polyHIPE networks showed typical 3D‐interconnected porous architecture with voids of 5–15 µm, windows of ≈1 µm, nanopores (70–100 nm) within the void walls, and high Brunauer–Emmett–Teller (BET) surface areas up to 750 m^2^ g^−1^.^[^ ^127^ ^]^

Non‐aqueous HIPEs have also been used to produce hydrophobic polyHIPEs. For example, macroporous hydrophobic polyurethane (PU) monoliths were prepared from water‐soluble mannitol using non‐aqueous HIPEs as templates.^[^ ^128^ ^]^ In this case, the HIPEs were formed by dropwise addition of paraffin oil into DMSO solution containing mannitol, poly(hexamethylene diisocyanate) (PHDI), and the triblock copolymer Pluronic P‐123 (Pluronic is a registered trademark of BASF). The polymerization of the HIPE was performed in a convection oven at 60 °C for 24 h. These highly macroporous monolithic polyHIPEs were hydrophobic with water contact angles between 102° and 140°.^[^ ^128^ ^]^ Instead of mannitol and Pluronic P‐123, the unmodified cellulose microparticles (50 µm) and triblock copolymer Pluronic F‐127 were used to generate robust cellulose‐based PU polyHIPEs with tunable wettability.^[^ ^129^ ^]^ The continuous phase of non‐aqueous paraffin oil‐in‐DMSO HIPE was used to prepare hierarchically macroporous PU from X‐shape block copolymer ethylenediamine tetrakis(ethoxylate‐*block*‐propoxylate) tetrol (T1107, monomer and stabilizer) and isocyanate via step‐growth polymerization.^[^ ^130^ ^]^ Similarly, other polycondensation chemistry using non‐aqueous HIPEs, such as 2,5‐dihydroxy‐1,4‐benzoquinone (DHBQ) with urea^[^ ^131^ ^]^ and melamine with formaldehyde,^[^ ^132^ ^]^ has been employed to prepare polyHIPEs.

Non‐aqueous HIPEs and ring‐opening polymerization were employed to prepare macroporous crosslinked PCL scaffolds in one step synthesis.^[^ ^133^ ^]^ The HIPEs were formed by dispersing hexadecane in the continuous phase containing monomer caprolactone, crosslinker bis(*ε*‐caprolactone‐4‐yl), and polymeric surfactant Pluronic F‐127. Interestingly, the porous scaffold showed better compressive loading (compared to the non‐porous crosslinked PCL) and very good osteoblast attachment, growth, and proliferation.^[^ ^133^ ^]^

#### 2.1.3 Hydrophilic Porous Polymers

Hydrophilic porous polymers can be similarly prepared from O/W HIPEs where monomers, crosslinkers, and surfactants are dissolved in the continuous aqueous phase. The most reported hydrophilic polyHIPEs have been polyacrylamide (PAM) with acrylamide (AM) as monomer and *N*,*N*′‐methylenebisacrylamide (MBAM) as crosslinker. The monomer and crosslinker are dissolved in the continuous aqueous phase along with an initiator [usually ammonium persulfate (APS) or KPS] and polymeric surfactant (e.g., Triton X‐405, Pluronic F‐127, and Pluronic P‐123) and/or particle stabilizers (e.g., SiO_2_ and TiO_2_ particles).^[^ ^97^, ^130^, ^134^, ^135^, ^136^, ^137^ ^]^ Often, *N*,*N*,*N*′,*N*′‐tetramethylethylenediamine (TMEDA) is added as a catalyst via a redox initiation mechanism to enhance the polymerization.^[^ ^97^, ^137^, ^138^, ^139^ ^]^ Non‐volatile oils such as mineral oil and paraffin oil are often used as the internal oil phase,^[^ ^97^, ^128^, ^129^, ^130^, ^131^, ^132^ ^]^ although there is also reported use of volatile organic solvents such as toluene and cyclohexane.^[^ ^116^ ^]^ AM can be replaced or copolymerized with other monomers to induce new functionality, for example, *N*‐isopropylacrylamide (NIPAM) for thermoresponsive property or AMPS for acidic group/ion exchange capacity.^[^ ^134^, ^137^, ^140^ ^]^

When exploring further grounds for hydrophilic polyHIPEs, AM has been the commonly used monomer as well. Pickering HIPEs, where particles are used as stabilizers and often assisted with small amounts of polymeric surfactants, have been used to prepare hydrophilic porous polymers. For example, poly(urethane urea) (PUU) NPs (52 nm),^[^ ^136^ ^]^ TiO_2_ NPs (P25, 20 nm, with small amount of Tween 85),^[^ ^135^ ^]^ HKUST‐1 (also known as MOF‐199 or Cu_3_(BTC)_2_, where HKUST stands for Hong Kong University of Science and Technology and BTC is 1,3,5‐benzenetricarboxylate) MOF NPs (≈200–500 nm, with poly(vinyl alcohol) (PVA) as co‐stabilizer),^[^ ^141^ ^]^ and UiO‐66 (UiO is the acronym for University of Oslo) MOF NPs (300–500 nm, with PVA as co‐stabilizer)^[^ ^142^ ^]^ are used to prepare PAM polyHIPEs. Greener solvents such as ILs and compressed CO_2_ are used to form emulsions, as a replacement to oils or organic solvents, which are subsequently used as templates for preparation of porous polymers. For example, an IL‐in‐water HIPE was employed to produce porous PAM.^[^ ^42^ ^]^ Macroporous polyacrylates were prepared from non‐aqueous HIPEs where an IL was added as the dispersed phase.^[^ ^143^ ^]^ CO_2_‐in‐IL emulsions were formed and employed as templates to fabricate high porous PAM and poly(trimethylolpropane trimethacrylate) with hierarchical macropores and mesopores.^[^ ^144^ ^]^ C/W HIPEs have also been used as templates to fabricate highly interconnected macroporous polymers. The challenge in forming stable C/W emulsions has been to develop effective and inexpensive polymeric stabilizer with the initial use of perfluoropolyether (PFPE) carboxylate and the later development of poly(vinyl acetate) (PVAc)‐based block copolymers as surfactants.^[^ ^37^ ^]^ These C/W emulsions have proven to be very efficient in producing highly porous PAM, PVA, and PVA/CS blends.^[^ ^37^, ^38^, ^39^ ^]^ In recent years, Pickering C/W emulsions, for example, via the use of TiO_2_ NPs^[^ ^134^, ^141^ ^]^ and MOF NPs,^[^ ^142^ ^]^ have also been employed to prepare porous PAM materials. For C/W HIPEs as templates, the main advantages include no use of organic solvent and facile removal of CO_2_ simply by depressurization while the key drawbacks still lie with the use of high pressure equipment (and associated hazards and costs) and relatively expensive surfactants.

Copolymerization of monomers or the incorporation of other polymers in O/W, HIPEs can produce porous polymers with additional properties. For example, copolymerization of AM and AMPS resulted in a porous monolith with –SO_3_H groups, which enhanced adsorption of methylene blue (MB) and tetracycline (TC) in wastewater.^[^ ^134^ ^]^ In another study, a gemini surfactant (sodium dilauramino cysteine) was synthesized and employed to prepare O/W HIPEs with AM, MBAM, and CS as doping macromolecules in the continuous phase. The prepared porous monoliths exhibited surface areas of 17.94 and 20.32 m^2^ g^−1^ calculated by BET and mercury (Hg) intrusion porosimetry methods, respectively.^[^ ^139^ ^]^ A similar procedure was used to synthesize CS‐PAA monolith by thermal polymerization of the HIPEs containing acrylic acid (AA) and CS (and MBAM and Tween 20 as surfactant) in the continuous aqueous phase. The reactions between the acid groups of PAA and then –NH_2_ groups of CS were proposed.^[^ ^94^ ^]^

The preparation of ionic macroporous polymers or hydrogels via O/W HIPE templating has been highly efficient. Silverstein and co‐authors prepared highly porous zwitterionic hydrogel polyHIPEs by using a commercially available zwitterionic monomer *N*‐(3‐sulfopropyl)‐*N*‐(methacryloxyethyl)‐*N,N*‐dimethylammonium betaine and MBAM as crosslinker. The macroporous structure of the zwitterionic hydrogels could be tuned by varying the fraction of the dispersed phase and the mole fraction of MBAM to the monomer. This macroporous structure enhanced water uptake, the anti‐polyelectrolyte effect, and the dual‐pH sensitivity.^[^ ^145^ ^]^ Kovacic et al. reported the preparation of highly porous AMPTMA‐based cationic polyelectrolytes.^[^ ^146^ ^]^ High concentration and accessibility of the cationic *N*‐quaternized groups in these polyelectrolyte polyHIPEs were demonstrated by high ion exchange capacity (3.53 mmol AgNO_3_/g dry polyHIPEs) and water uptake (95 g g^−1^).^[^ ^146^ ^]^ In a further study, macroporous polyampholyte hydrogels were prepared by mixing two HIPEs containing oppositely charged monomers AMPTMA and AMPS.^[^ ^147^ ^]^ Different ways of mixing AMPTMA and AMPS were investigated (Figure 6d,e): i) dissolving AMPTMA and AMPS together in the continuous aqueous phase for co‐polyHIPEs;^[^ ^148^ ^]^ ii) two pre‐formed HIPEs were placed one on top of the other for the preparation of bilayered polyHIPEs; and iii) two pre‐formed HIPEs were mixed and stirred to prepare mixed polyHIPEs. Anti‐polyelectrolyte behavior was shown by both the co‐polyHIPEs and mixed polyHIPEs, whereas the bilayered polyHIPEs performed like polyelectrolytes.^[^ ^147^ ^]^

Preparation of ionic polyHIPEs has been extended to porous poly(ionic liquids) (PILs). Porous PILs are solid and strong polyelectrolytes and have found applications in many areas such as catalysis, separation, energy harvesting, and bio‐related applications.^[^ ^149^ ^]^ Debuigne and co‐authors first used sugar‐based fluorinated surfactants to create C/W emulsions.^[^ ^150^ ^]^ PIL polyHIPEs were fabricated by using the HIPEs as templates, which contained 1‐vinyl‐3‐ethylimidazolium bromide (monomer) and divinylimidazolium (crosslinker) in the water phase.^[^ ^150^ ^]^ The same group then used CO_2_‐in‐IL emulsions to fabricate PIL/IL composites.^[^ ^151^ ^]^ In a recent study, cyclohexane‐in‐water HIPE‐templated interconnected macroporous imidazolium‐based PIL monoliths were produced through a one‐pot modified Radziszewski multicomponent polymerization. The resulting materials possessed connected cavities and were demonstrated as a high‐performance heterogeneous catalyst for decarboxylation of caffeic acid and transesterification reactions.^[^ ^152^ ^]^

Considering their uses in biomedical applications,^[^ ^26^, ^29^ ^]^ Pickering HIPEs are also good platforms for the preparation of biocompatible polyHIPEs.^[^ ^153^ ^]^ This is because a wide variety of particles with natural origin are available to form Pickering emulsions. For example, the natural, food‐grade, and edible denatured casein (a phosphoprotein) NPs were used as a Pickering‐type interfacial emulsifier to fabricate hierarchically porous molecularly imprinted polymers.^[^ ^154^ ^]^ The Pickering emulsions were prepared by mixing the aqueous phase mixture (composed of denatured casein NPs, dopamine, and bovine hemoglobin (BHb) with the corn oil phase followed by the addition of APS as initiator. This method demonstrated a step forward to design and fabricate natural‐protein‐based structured emulsions to prepare polyHIPEs.^[^ ^154^ ^]^ Pretreated yeast particles (treating original baker's yeast with a NaOH solution, particle sizes of about 5 µm) were used to stabilize O/W emulsions with AA as monomer and MBAM as crosslinker (Figure 6f). The prepared superporous polymeric materials displayed interconnected pores, abundant –COO^—^ groups, and superior stability owing to the affinity between the yeast and PAA.^[^ ^155^ ^]^

In addition to the commonly used AM and AA monomers, some acrylate monomers have also been used to fabricate hydrophilic polyHIPEs. Porous polyester‐glycidyl methacrylate (PE‐GMA) monoliths were prepared from W/O HIPEs, which were formed by dropwise addition of water into the mixture of unsaturated polyester resin, crosslinkers glycidyl methacrylate‐divinylbenzene or glycidyl methacrylate‐styrene, triethanolamine TEA as emulsifier, AIBN, and a porogen [toluene, tetrahydrofuran (THF) or CB].^[^ ^156^ ^]^ The cream‐like emulsions were cured at 80 °C in a polyethylene mold and subsequently washed further by Soxhlet extraction in methanol. The PE‐GMA‐based polyHIPE monoliths with epoxy groups were then functionalized with different types of amines including EDA, hexamethylenediamine, 4‐aminosalicylic acid, 2‐aminothiazole, 4‐aminobenzothiazole, and 2‐phenylimidazole for heavy metal removal. The surface areas of these porous monoliths varied in the range of 11.32–76.14 m^2^ g^−1^ depending on the types of monomers and porogens used.^[^ ^156^ ^]^

Macroporous poly(2‐dimethylaminoethyl methacrylate) (PDMAEMA) was prepared from a W/O HIPE. The HIPEs used for PDMAEMA included monomer DMAEMA, crosslinker EGDMA, surfactant Pluronic L‐121, and initiator AIBN in the continuous oil phase.^[^ ^157^ ^]^ In the same study, poly(2‐hydroxyethyl methacrylate) (PHEMA) was produced using O/W HIPEs where cyclohexane was the oil phase and 2‐hydroxyethyl methacrylate (HEMA), MBAM, APS, and Pluronic F‐68 were dissolved in the continuous aqueous phase. The resulting monoliths exhibited cellular porous structures with relatively low BET surface areas of 23.4 m^2^ g^−1^ (PDMAEMA) and 10.1 m^2^ g^−1^ (PHEMA) owing to the presence of macropores.^[^ ^157^ ^]^

In addition to polymerizing the monomers/crosslinkers in the continuous phase of the HIPEs, emulsions can be frozen rapidly to lock in the emulsion structure. A subsequent freeze drying process produces emulsion‐templated pores with additional ice‐templated pores, enhancing porosity and pore connectivity.^[^ ^84^ ^]^ The combination of emulsion templating and ice templating gives rise to a new approach to fabricating porous materials. For example, by varying the volume percentage of droplet phase (0–75 v/v%) and the concentration of the continuous phase in O/W emulsions, it was possible to systematically tune the porosity and pore size of the resulting porous materials. Moreover, the freeze‐dried materials could also be post‐treated by crosslinking in order to improve their chemical and mechanical stability.^[^ ^158^ ^]^

Another advantage of combining emulsion templating and ice templating is that the composite materials with emulsion‐templated structures can be readily produced. For example, by dissolving hydrophobic compounds (such as organic dyes, poorly water‐soluble drugs, etc.) in the droplet phase of O/W emulsions, organic NPs within porous polymers are generated in situ after freeze drying the emulsions.^[^ ^82^, ^83^, ^159^ ^]^ More importantly, the porous polymer scaffolds can be instantly dissolved in water, thereby generating aqueous NPs dispersions having great potential for nanomedicine.^[^ ^82^, ^160^ ^]^

#### 2.1.4 PolyHIPE Beads and Porous Microspheres

PolyHIPEs can be easily prepared as monoliths, conforming to the molds or vessels used. However, the fabrication of polyHIPE beads or microspheres can offer additional advantages such as easy handling, efficient packing, or being included in injection formulations for biomedical applications.^[^ ^78^, ^97^, ^161^, ^162^, ^163^ ^]^ Suspension polymerization is commonly used for preparation of polymer beads and porous particles where the use of porogens (e.g., solvent, polymer, etc.) or other solid templates is often essential for the production of porous microspheres.^[^ ^89^, ^164^, ^165^ ^]^ However, to use the emulsion templating method to prepare porous or polyHIPE beads/spheres, a pre‐formed O/W (or W/O) emulsion is required to be dispersed in another oil (or water) phase. The polymerization or solidification of the continuous phase of the initial/primary emulsion results in the formation of emulsion‐templated porous spheres. When the primary emulsion is not the HIPE, microspheres with hollow compartments or isolated pores (instead of highly interconnected pore structure) are produced.^[^ ^166^, ^167^ ^]^ The double emulsions are usually formed by a two‐step approach, that is, dispersing a pre‐formed primary emulsion into another continuous phase.^[^ ^54^, ^56^, ^166^, ^167^ ^]^

**Figure 7:**
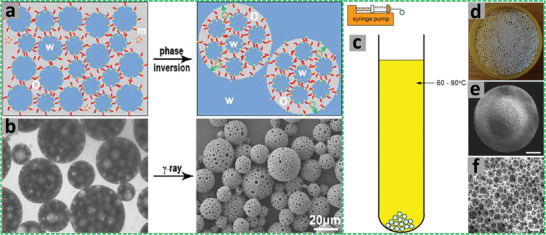
a) The scheme shows phase inversion of W/O HIPE used to prepare HIPE W/O/W double emulsions. b) Polymerization of the monomer (oil) phase to generate polyHIPE microspheres. Reproduced with permission.^[^ ^168^ ^]^ Copyright 2014, American Chemical Society. c) Preparation of polyHIPE PAM beads by O/W/O sedimentation polymerization: The scheme shows a pre‐formed O/W HIPE that is injected into a glass column containing hot oil. Adapted with permission.^[^ ^23^ ^]^ Copyright 2005, Royal Society of Chemistry. d) Uniform PAM beads with diameters around 1.5 mm; e) whole bead (scale bar = 500 *µ*m); f) highly porous surface and internal structure of the sectioned bead. Reproduced with permission.^[^ ^97^ ^]^ Copyright 2002, American Chemical Society.

A one‐step preparation method for high internal water‐phase double emulsions was reported via phase inversion of W/O HIPEs, which were formed by the dropwise addition of water into the oil phase containing St as monomer (and other acrylate co‐monomers), EGDMA as crosslinker, and ammonia‐neutralized 12‐acryloxy‐9‐octadecenoic acid (AOA) as surfactant. The phase inversion occurred with the addition of water in multiple steps until a sudden change of emulsion conductivity was observed.^[^ ^168^ ^]^ This process only employed one surfactant, indicating the neutralized‐AOA could stabilize both O/W and W/O emulsions. The polymerization of the monomer phase by *γ*‐ray irradiation produced polyHIPE microspheres in the diameter range of 10–20 µm (**Figure** **7**a,b).^[^ ^168^ ^]^ Recently, another example of employing phase inversion and pine pollen as the only stabilizer for the preparation of porous microspheres was reported.^[^ ^169^ ^]^

However, the formation of double emulsions with either one‐step or two‐step methods while maintaining the emulsion stability during polymerization is still quite challenging. Careful choice of the surfactants and optimized preparation conditions are usually required. This problem can be addressed by employing an O/W/O (or W/O/W) sedimentation polymerization approach.^[^ ^97^, ^162^ ^]^ Figure 7c shows how an O/W/O process can be used to prepare polyHIPE PAM beads. In this process, an O/W HIPE is prepared and injected into a hot oil medium. During the sedimentation process, the HIPE drops are partially polymerized and discrete beads are formed at the bottom of the oil medium. These beads are kept in the hot oil medium for longer period to allow for complete polymerization of monomers and crosslinkers before collecting and washing.^[^ ^23^ ^]^ Uniform porous PAM beads with diameters of around 1.5 mm are generated showing highly porous surface and internal structure (Figure 7d–f).^[^ ^97^ ^]^ Similarly, Wang et al. employed a W/O/W sedimentation process to prepare enzyme‐immobilized poly(glycidyl methacrylate) (PGMA) polyHIPE beads, which were packed into a reactor for the esterification reaction between hexanoic acid and 1‐hexanol. The prepared W/O HIPE was injected into an aqueous PVA (3 wt%) solution drop by drop and the polymerization was achieved by ultraviolet (UV) irradiation for 5 h at RT.^[^ ^162^ ^]^

**Figure 8:**
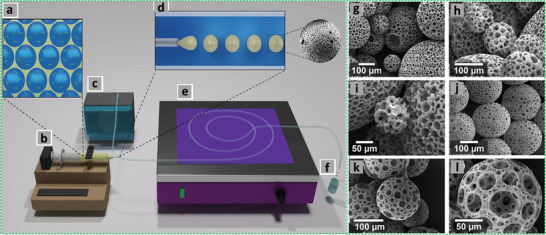
Schematic representation for the preparation of polyHIPE microspheres by a fluidics approach: a) HIPE is injected using b) a syringe pump into a flowing fluid in d) a tubing controlled by c) a peristaltic pump; e) the HIPE drops are polymerized under UV irradiation and subsequently f) collected in a container. Reproduced with permission.^[^ ^161^ ^]^ Copyright 2019, Elsevier. g–i) SEM images of the microspheres prepared by the controlled stirred‐tank reactor (CSTR) method; j–l) SEM images of the microspheres fabricated by the microfluidics approach. Reproduced under a Creative Commons Attribution (CC BY) license.^[^ ^78^ ^]^ Copyright 2018, The Author(s). Published by AIP Publishing.

In order to improve productivity of polyHIPE microspheres and the ability to vary the size of these microspheres, a fluidics (or microfluidics) approach has been developed.^[^ ^78^, ^161^, ^163^, ^170^ ^]^ **Figure** **8**a–f illustrates how the preparation of polyHIPE microspheres can be achieved by a fluidics approach.^[^ ^161^ ^]^ Usually, the HIPE is first prepared and then injected into plastic tubing containing a flowing fluid with controlled flow rate. Due to the thin tubing used, the HIPE drops can be efficiently polymerized under light irradiation. This process has been mostly used for W/O emulsions where the flowing fluid is aqueous solution with a stabilizer. PVA has shown to be the most effective stabilizer for the preparation of uniform microspheres. Gokmen et al. prepared “clickable” PGMA polyHIPE microspheres with diameters down to 400 µm.^[^ ^163^ ^]^ Cosgriff‐Hernandez and co‐authors reported the preparation of PEGDMA polyHIPE microspheres with encapsulated bone morphogenetic protein 2 for controlled release. The particle size (200–800 µm) and pore size (10–30 µm) of the PEGDMA microspheres were tunable.^[^ ^170^ ^]^ This group further investigated the effect of particle size (400 vs 800 µm) and pore size (15 vs 30 µm) on loading efficiency of growth factors and the delivery of such growth factors as an injectable bone graft.^[^ ^161^ ^]^ A primary W/O HIPE with macromer EGDMA and photoinitiator DMPA in the continuous phase was injected dropwise into a flowing aqueous solution containing 3 wt% PVA. The passing emulsion drops were polymerized by UV irradiation. Parameters such as needle gauge, flow rate, surfactant concentration, and emulsion formulation could be varied to produce particles with adjustable particle diameters and pore sizes.^[^ ^161^ ^]^

Paterson et al. compared the Poly(2‐ethylhexyl acrylate‐isobornyl acrylate) P(EHA‐IBOA) microspheres (from a blend of EHA and IBOA) prepared by the T‐junction microfluidics approach and the CSTR method.^[^ ^78^ ^]^ The formed W/O HIPE was added into a 100 mL beaker containing deionized water with an overhead stirrer (CSTR method) or injected into a 6 mm diameter silicon tube with flowing deionized water. A Hg lamp with the UV output was used to cure the HIPE drops. It showed that the microfluidics approach could generate microspheres with narrower particle size distribution. For the microfluidics approach, the microsphere diameters could be tuned in the range of about 200–350 µm by varying water flow rate (125–745 cm^3^ min^−1^). In this regard, both methods were used to produce highly porous microspheres (Figure 8g–l). The surface pore morphology of the microspheres achieved by both these methods was clearly different from each other. In the microfluidics approach, the contact of the HIPE with the syringe surface and the metal needle could lead to certain surface destabilization and hence a thin film of monomers around HIPE drops. The internal porosity and pore size of these polyHIPE microspheres were not significantly different as they were determined by the emulsion formulation.^[^ ^78^ ^]^

#### 2.1.5 Summary

Section 2.1 provides review of the existing literature on the preparation methods, conditions, and porosity results of non‐aqueous, W/O, O/W, C/W, W/W, O/W/O, and W/O/W emulsion‐templated hydrophobic (non‐degradable and degradable) and hydrophilic porous polymers. Section 2.1 reveals that the St monomer‐based W/O type emulsions have extensively been studied to produce hydrophobic emulsion‐templated polymers. Most of the reported emulsion‐templated polymers such as microspheres, beads, and monoliths are polyHIPEs. The pore (including voids and windows) size of the reported polyHIPEs extends between 0.9–50 µm with average diameter of 25.45 µm, whereas some materials also exhibited nanopores of 5–13 and 70–100 nm. The pore volume of some materials came out to be 5.9–29.67 cm^3^ g^−1^. Although polyHIPEs usually show surface areas in the 3–20 m^2^ g^−1^, but some of the emulsion‐templated porous polymers displayed a high surface area of 150–750 m^2^ g^−1^ mainly due to the presence of nanopores. The reported density of many materials falls between 0.03 and 0.29 g cm^−3^, but it reached the value of 0.66 g cm^−3^ in one material. A summary of the preparation conditions and porosity results of the emulsion‐templated porous polymers are presented in **Table** **1**.

**Table 1:** Summary of preparation methods and main properties of polymer polyHIPEs

Materials	Preparation	Porosity/Property^a)^	Ref.
P(St‐*co*‐DVB)	W/O HIPEs, pure water as internal phase, AIBN initiator, Span 80 as surfactant Other crosslinkers used	Pore diameter: 20–22 µm, pore volume: up to 8.7 cm^3^ g^−1^ Pore size and porosity varied depending on conditions	[107, 108, 109, 110, 111]
sPS‐based nanofibrous monoliths	Glycerol‐in‐TCB, sulfonated sPS as stabilizer Emulsions prepared at 120 °C HIPEs solidified at RT	Pore wall consisting of nanofibers Voids: 10 µm), throats: 1–2 µm Surface area: 420 m^2^ g^−1^	[112]
P(St‐*co*‐VBC‐*co*‐DVB)	W/O emulsions, Span 80 surfactant Further modification with (4VP), P4VP, and amines	Pore size: = 6.8–8.6 µm	[113, 114, 115, 116]
P(St‐*co*‐MMA)	W/O emulsion, Span 20 as surfactant Polymerized at 60 °C, 48 h.	Voids: 3.0–7.4 µm Windows: 1.1–2.4 µm Monolith filter	[118]
P(St‐*co*‐NPA) beads	W/O emulsions, Span 80/85 as surfactant, CB also in the oil phase Further modification with piperazine	Voids and windows: 1.5 µm	[117]
P(St‐*co*‐DEAEMA) membrane	W/O emulsions, Span 80 Emulsions cast into a mold for polyHIPE membrane	N content: up to 2.13% by X‐ray photoelectron spectroscopy (XPS) Void diameter: 3.5–4.8 µm Window size: 0.9–1.4 µm CO_2_‐switchable properties	[119]
P(St‐EGDMA)	W/O emulsion with 93% water phase POEGMA‐*b*‐PS as stabilizer	Pore sizes: 5–50 µm Minimum density of 0.08 g cm^−3^ Water contact angle of ≈120°	[120]
P(St‐DVB‐EHA) [poly(styrene‐divinylbenzene‐2‐ethylhexyl acrylate)] membrane	W/O emulsions, Span 80 Emulsions cast into the space between two flat plates for membrane preparation	Further modified as electrodes for ion and molecular recognition	[121]
P(PEM‐St‐DVB)	W/O emulsions, Span 80 Fluorinated monomer to provide low surface energy	Superhydrophobicity (water contact angle: 151°) and oleophilicity Void size: = 6.2–27 µm	[122]
P(IDA‐*co*‐DVB) monolithic column	W/O emulsion, Span 80, 90% water phase; HIPEs imbibed into silica capillary under 2.5 bar	Interconnected voids: 8.9 ± 2.7 µm, windows: 2.0 ± 0.9 µm Surface area: 5.44 m^2^ g^−1^	[123]
Poly(vinylbenzyl chloride‐divinylbenzene) [P(VBC‐DVB)]	W/O emulsions, Span 80/SDBS/CTAB mixture as stabilizer, 90% water phase, chloroethylbenzene as porogen Further modification by amination	Surface area: 150 m^2^ g^−1^	[124]
PAMAM dendrimer‐SPS‐*b*‐PE‐*r*‐Bt‐*b*‐PS	W/O HIPEs, no other components Gelation by ionic interaction Freeze‐dried porous materials Similar approach applied to PPI dendrimer and SPS‐*b*‐PE‐*r*‐Bt‐*b*‐PS	Interconnected macropores: 1–20 µm Density: 0.08–0.1 g cm^−3^	[126, 171]
PAE	W/O emulsions, Span 80 Sonogashira cross‐coupling in continuous phase	Macropores: 70–100 nm, voids: 5–15 µm, and windows: ≈1 µm Surface area: 750 m^2^ g^−1^	[127]
Mannitol‐based PU	Non‐aqueous emulsion (paraffin oil in DMSO solution), mannitol, PHDI, and Pluronic P‐123 in DMSO, internal phase 75–85% Polymerized at 60 °C, 24 h	Void diameters: 1.3–1.7 µm, density: 0.13–0.20 g cm^−3^, window size: 0.3–0.4 µm	[29]
Cellulose‐based PU	Non‐aqueous emulsion (paraffin oil in DMSO solution), cellulose and Pluronic F‐127 in DMSO, internal phase 75%, crosslinking between isocyanates and cellulose	Density: 0.10–0.13 g cm^−3^	[129]
PU	Non‐aqueous emulsion (paraffin oil in DMSO solution), PPIF and reactive stabilizer T1107 in DMSO, internal phase volume 75–80% Polymerized at 60 °C, 24 h	Surface areas: 17–31 m^2^ g^−1^ Density: 0.103–0.187 g cm^−3^	[130]
P(DHBQ‐urea)	O/W emulsions, Triton X‐405, 80.5% internal phase Deep‐eutectic polymer formed and used Chain extension improving stability	Surface area: 216 m^2^ g^−1^ Carbon precursor	[131]
PMF [poly(melamine–formaldehyde)]	Isopar M‐in‐DMSO emulsion, Pluronic F‐127 as surfactant, internal phase up to 85% Polycondensation between melamine and formaldehyde Polymerized at 170 °C, 3 days Further co‐ordination with Zn^2+^	Surface area: 497 m^2^ g^−1^ Adsorb CO_2_ and then react with epoxides	[132]
Poly(caprolactone)	O(hexadecane)/O HIPEs, caprolactone as monomer, Sn(Oct)_2_ as catalyst, Pluronic F‐127, bis(*ε*‐caprolactone‐4‐yl)	Density: 0.29–0.66 g cm^−3^, pore size: 13–49 µm, Young's modulus: 1.2–12 MPa	[133]
PAM and PVA	C/W emulsions, CO_2_ volume fraction 70–80%, PFPE ammonium carboxylate	Pore volume up to 5.9 cm^3^ g^−1^	[37]
PVA	C/W emulsions with PVAc‐based block copolymer, internal phase up to 79%, crosslinked with glutaraldehyde	Pore volume: 17.0 cm^3^ g^−1^, bulk density: 0.043 g cm^−3^, pore size: 12 µm	[39]
PAM	O(liquid paraffin)/W emulsion, internal phase up to 92%, PUU NPs‐stabilized Pickering emulsion	Void diameter: 11–83 µm by SEM imaging, interconnecting pore diameter: 1.5–14 µm by Hg intrusion porosimetry	[136]
PAM	CO_2_‐in‐IL emulsion	Surface area: up to 152 m^2^ g^−1^, porosity: up to 88%, macropore diameter: 2–14 µm, mesopore diameter ≈5–13 nm	[144]
PAM	IL‐in‐water emulsion, internal phase 90%		[42]
Poly(2‐acrylamide‐2‐methylpropanesulfonic acid) (PAMPS)	O(toluene)/W emulsion, internal phase up 67–86%, AMPS and MBAM, Pluronic F‐127	Swollen gels compressive strains up to 60%, dry gels high stresses at 70% strain	[137]
CS‐PAM monoliths	CS added into the aqueous phase of O/W emulsions Gemini surfactant used	Void sizes: 3.4–6.6 µm Foam density: 0.10–0.28 g cm^−3^ Surface area: 10.06 to 17.94 m^2^ g^−1^	[139]
Polyelectrolyte	O/W emulsions with AMPTMA as monomer, MBAM, F‐108 as surfactant, toluene as oil phase	Density: 0.23–0.27 g cm^−3^ Void sizes: 11–13 µm Measured accessible –NR_3_^+^ groups: 3.53 mmol g^−1^	[146]
Polyampholytes	O(toluene)/W emulsion, AMPS and AMPTMA as monomers, MBAM, F‐108, Co‐polymer, mix, and bilayered structures	Density: 0.18 to 0.23 g cm^−3^, carrying both negative and positive charges	[147]
PILs	CO_2_‐in‐IL emulsion, surfactant free and surfactant assisted	Macroporous monoliths with anion exchange	[151]
Molecularly imprinted polymers	Pickering corn O/W 50:50 emulsion Casein NPs as stabilizer Prepared from dopamine and BHb Polymerization initiated by APS, RT Template BHb removed by acetic acid/sodium dodecyl sulfate (SDS) solution	Interconnected macroporous structure Selective protein recognition	[154]
Yeast‐PAA	O/W HIPEs Canola oil as internal phase Pretreated yeast as stabilizer	Void size decreases from 50 µm to 9 µm with increasing yeast concentration from 0.5 to 4%, thereby increasing pore interconnectivity Abundant –COO^−^/‐COOH groups	[155]
Polymethacrylate	IL‐in‐O emulsion, lauryl methacrylate as monomer, Arlacel P135 as surfactant; IL phase extracted with extensive washes	Surface area: up to 10 m^2^ g^−1^, pore size ≈2 µm, porosity: 84%	[143]
PDMAEMA and PHEMA	W/O (surfactant Pluronic F‐121, crosslinker EGDMA) and O/W (surfactant Pluronic F‐68, crosslinker MBAM) HIPEs respectively	Up to 6 times w/w water adsorption	[157]
Sodium carboxymethylcellulose (SCMC)	O/W emulsions with varying oil percentages 0 75 v/v% Volatile cyclohexane as internal phase Emulsion freeze‐drying approach	Emulsion‐templated and ice‐templated pores; Control pore morphology and interconnectivity Pore volumes: 7.75–29.67 cm^3^ g^−1^ Density: 0.031–0.11 g cm^−3^	[158]
P(St‐EGDMA) microspheres	Phase inversion of W/O HIPEs to (W/O HIPEs)/W double emulsion, AOA as surfactant. Other monomers used as well	Particle size ≈19 µm	[168]
PAM microspheres	Phase inversion of O/W emulsion to O/W/O double emulsion, pine pollen as stabilizer	Particle sizes: 1–8 µm (average 2.6 µm) Porous microspheres	[172]
PAM beads	O/W/O sedimentation polymerization	Highly interconnected porous beads of about 2 mm. Pore volume: up to 8 cm^3^ g^−1^ Lowest density: 0.09 g cm^−3^	[161]
PGMA beads	W/O/W sedimentation polymerization Enzyme immobilized	Bead size: around 3.5 mm	[162]
PEGDMA microspheres	Microfluidics approach, W/O HIPE with EDGMA macromere and polyglycerol polyricinoleate as surfactant, injected into aqueous medium with 3 wt% PVA, UV irradiation	Particle size: 400 and 800 µm, pore size: 15 and 30 µm Osteoinductive factor encapsulation	[161]
P(EHA‐IBOA) microspheres	Monomers EHA and IBOA, crosslinker trimethylolpropane triacrylate microfluidics approach with deionized water Compared with the CSTR method	Particle size: 200–355 µm for flow rates 700 down to 125 cm^3^ min^−1^ Highly porous internally and externally Pore diameters: 2–35 µm, mostly of 6–10 µm	[78]
Polyacrylates microspheres and rods	Microfluidic approach by injecting W/O emulsion into aqueous medium Shape control by emulsion viscosity and stabilizers in aqueous medium Post‐functionalization by click‐click approach	Microspheres: around 400 µm Surface area: 49 m^2^ g^−1^	[163]

^a)^ Surface area values are for BET surface areas and pore volumes are measured by Hg intrusion porosimetry. Bulk density data are always quoted here for “density”.

### 2.2 Organic–Inorganic Hybrids/Composites

Polymer materials are of paramount importance in industry owing to their lightweight, often ductile nature, and synthetic ease. Since the polymers possess relatively low modulus and strength than those of the ceramics and metals, the fibers, whiskers, platelets, or particles are added to the polymer matrix for improving their mechanical strength. In this perspective, polymers are filled with different types of inorganic compounds (synthetic or natural) to enhance heat and impact resistance, flame retardancy, and mechanical strength. Moreover, the inclusion of inorganic components imparts intriguing properties (optical, electronic, catalytic, magnetic, etc.) to the resultant hybrids and composite materials.^[^ ^173^ ^]^

Both hybrids and composites refer to the materials that contain multiple components of different properties and functionalities.^[^ ^174^, ^175^, ^176^ ^]^ Generally, the hybrids are defined as the materials with the components blended at molecular or nanosized levels while composite materials cover the blending of components at both microscopic and macroscopic levels. However, the terms “hybrid” and “composite” are interchangeably used by researchers regardless of microscopic and macroscopic nature of the constituents.^[^ ^175^ ^]^ To the best of our knowledge, most review articles have been focusing on synthesis of emulsion‐templated porous polymer systems. However, in recent years, a great deal of work has been devoted to the design‐led synthesis of emulsion‐templated porous organic–inorganic hybrid/composite materials.

Such hybrids and composites represent two worlds (organic and inorganic) of chemistry each with distinctive properties. The key motivation behind the design and synthesis of organic–inorganic hybrids and composites is to integrate the finest properties and to alleviate the drawbacks of each counterpart because the organic materials bear a wide variety of different functionalities and can be easily molded into different shapes of varying sizes but lack thermally stability, whereas the inorganic materials exhibit greater thermal and mechanical stability with an enriched chemistry but their shaping in the macroscale size regimes to realize practical environmental remediation is a challenging task.^[^ ^25^ ^]^

The chemistry, dimensions, physicochemical and thermomechanical properties, porous structure, shape, morphology, and size of the porous emulsion‐templated hybrids/composites can be adjusted by altering the nature and chemical composition of inorganic and organic counterparts, type of interfacial interaction, and size and distribution of pores and particles using different preparation methods under certain reaction conditions, etc.^[^ ^174^, ^175^, ^176^, ^177^ ^]^ The preparation of such organic–inorganic hybrids/composites involves different techniques including filling, grafting, and copolymerization. Among these strategies, the filling of polymers with the inorganic constituents (fillers) involves no chemical bond, whereas the grafting approach develops chemical bonds between the inorganic and organic components. The inorganic moieties can also act as the crosslinking centers to generate multiple chemical bonds with organic polymers via a copolymerization method.^[^ ^25^, ^29^, ^174^, ^175^, ^176^ ^]^

The emulsion‐templated porous hybrids and composites usually encompass organic polymers with metallic or non‐metallic oxide networks, clusters, metal complexes, or NPs or MOFs or clays. Such hybrid/composite porous materials are composed of the organic polymeric network plus inorganic structure or inorganic network with organic constituents. This new class of hybrids/composites materials exhibit high thermal/chemical stability, mechanical strength, surface area, reactivity, reaction rates, and selectivity for multi‐purpose applications owing to the interfacial characteristics, combined host–guest chemistry, and synergetic/complementary effects.^[^ ^23^, ^25^, ^174^ ^]^ The strong entanglement of inorganic particles into the millimeter size porous organic polymer scaffolds overcomes the problem of trade‐off between effective remediation and secondary contamination and eases their complicated processability during and after the application tests, thereby making such materials practically valuable especially to develop decentralized water treatment systems.^[^ ^103^ ^]^

The following subsections provide a detailed discussion on the preparation methods of the emulsion‐templated porous hybrid and composite materials.

#### 2.2.1 Silica‐Organic Hybrids

Silica‐organic (polymer) hybrids are probably the most widely prepared hybrid materials.^[^ ^29^, ^174^ ^]^ The one‐step method is often employed because of the enhanced interaction between silica and organic polymer or the highly efficient interpenetration network. For the one‐step preparation of emulsion‐templated silica‐polymer hybrids, organo‐silanes are usually dissolved in the oil phase (due to their hydrophobicity) of W/O emulsions or the hydrolyzed silica sol is mixed into the aqueous phase of O/W emulsions. Alternatively, silica NPs are used as stabilizers (sometimes in combination with a polymeric surfactant) for Pickering HIPEs and polymerization of such HIPEs produces silica‐polymer polyHIPEs.

Silsesquioxanes are Si‐containing organic compounds with the generic formula of (RSiO_3/2_)n, where R can be hydrogen or other groups.^[^ ^178^ ^]^ There are different types of structures for silsesquioxanes including random, ladder, and cage structures. Polyhedral oligomeric silsesquioxane (POSS), usually regarded as the smallest silica colloid (1–3 nm), has been widely investigated in order to improve thermal and mechanical stability of materials.^[^ ^179^ ^]^ Vinyl silsesquioxane (VSQ) and POSS were combined with monomer EHA to prepare porous polyHIPE composites. The polyHIPEs containing POSS showed higher tan *δ* peak temperature and moduli than those of the EHA‐containing composites.^[^ ^180^ ^]^ Similarly, polyHIPE nanocomposites (NCs) with VSQ and methylsilsesquioxane (MSQ) were prepared. Since VSQ was copolymerized with EHA/DVB, better properties were observed for VSQ‐containing polyHIPEs.^[^ ^177^ ^]^ Chen et al. synthesized and then used a ferrocene derivative of cholesterol as the stabilizer (also acting as a low‐molecular mass gelator, LMMG) for the preparation of W/O HIPEs. Tetraethyl orthosilicate (TEOS) was introduced into the continuous monomer *tert*‐butyl methacrylate phase. As a result, the mechanical stability of the resulting hybrid polyHIPEs was significantly improved.^[^ ^181^ ^]^

Alternatively, hydrophobic organo‐silanes can be hydrolyzed under acidic conditions and the obtained silica sol can then be introduced into the continuous phase of O/W HIPEs. For example, polyHIPE silica hybrid beads were prepared by adding TEOS sol into the aqueous phase of an O/W HIPE containing AM monomer and MBAM crosslinker followed by an O/W/O sedimentation polymerization.^[^ ^182^ ^]^ Backov and co‐authors prepared the hybrid polyHIPE, i.e., organo‐Si(HIPE), monoliths with a trimodal (micro, meso, and macro) porous structure.^[^ ^183^ ^]^ Different organosilanes containing various functional groups (methyl, dinitrophenylamino, benzyl, pyrrol, and mercaptopropyl) were hydrolyzed under acidic conditions until a clear aqueous sol was formed. Dodecane was then added dropwise into the aqueous phase. The resulting emulsions were allowed to condense at RT. The dry porous hybrid materials exhibited greater integrity, good mechanical stability, and tunable functionalities.^[^ ^183^ ^]^

Another one‐step approach is to employ Pickering HIPEs with silica NPs as stabilizers. Bismarck and co‐authors modified hydrophilic silica NPs (20–100 nm) by treatment with chloroform/oleic acid (OA). The modified silica NPs could stabilize W/O HIPEs (with St and poly(ethylene glycol) dimethacrylate as the continuous phase) with internal phase volume percentage up to 92 v/v%. Porous hybrid materials could thus be prepared by polymerizing the continuous phase.^[^ ^184^ ^]^ By employing the MIPEs (70 v/v% internal phase) and HIPEs as templates, the same group was able to produce strong porous hybrid materials with high and tunable gas permeability. The use of silica NPs as stabilizer was responsible for the high mechanical strength while the addition of a small amount of polymeric surfactant (Hypermer 2296) facilitated the formation of highly connected macroporous structure.^[^ ^185^ ^]^ Matyjaszewski and co‐authors fabricated highly porous materials for CO_2_ capture via HIPE polymerization, wherein an internal aqueous phase was mixed with silica particles and Span 80 followed by the copolymerization of *p*‐chloromethylstyrene with DVB.^[^ ^186^ ^]^ Instead of directly being used as stabilizer, hydrophobic silica NPs were dispersed into the continuous oil phase containing AIBN, ST, DVB, and *n*‐octyltriethoxysilane (*n*‐OTES) of a W/O HIPE with acidified cotton (treated by concentrated sulfuric acid) used as a stabilizer. This enhanced the performance and regeneration of the resulting porous composites as adsorbents for the removal of particulate matters.^[^ ^187^ ^]^ In addition to using silica particles as stabilizers, it is also possible to use Si‐containing polymers as surfactant to form HIPEs for the direct formation of porous hybrid materials. Ye et al. employed polyethoxysiloxane (PEOS) as sole stabilizer when preparing the W/O HIPEs. They also added silica precursors N‐[3‐(trimethoxysilyl)propyl]ethylenediamine and (3‐glycidyloxypropyl)trimethoxysilane (GPTMS) into the aqueous phase (containing AA, APS, and 3‐methacryloxypropytrimethoxysilane (MPS) to facilitate the subsequent modifications. The prepared porous materials were found to contain micro‐, meso‐, and macropores. The surface areas of amino‐, epoxy‐, and carboxyl‐functionalized silica foams came out to be 450, 481, and 500 m^2^ g^−1^, respectively. The porous silica foams showed good mechanical integrity at low aqueous pH values (1–5) of HIPEs.^[^ ^172^ ^]^

**Figure 9:**
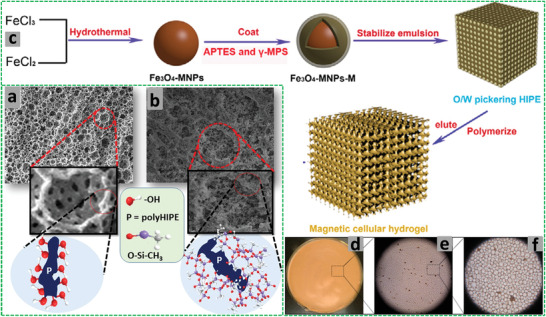
Schematic representation of the a) hydroxyl terminated polyHIPE and b) covalently bonded silica−polyHIPE composite networks. Reproduced under a Creative Commons Attribution 4.0.^[^ ^190^ ^]^ Copyright 2017, The Authors(s), published by Springer Nature. c) Schematic illustration for the preparation of monolithic magnetic macroporous CS‐PAA hydrogel. d) Digital photograph and optical microscope images of Fe_3_O_4_‐stabilized Pickering emulsion at a e) magnification of 100 and a f) magnification of 1000. Adapted with permission.^[^ ^192^ ^]^ Copyright 2016, Elsevier.

The two‐step approach to the preparation of polyHIPE silica‐polymer composites usually involves first the preparation of a polyHIPE porous polymer and subsequently the impregnation of a silica precursor into the polymer polyHIPE. For example, porous PAM was immersed in silica sol or silica colloidal suspensions. After gelation and drying, porous PAM/silica composites were formed.^[^ ^188^, ^189^ ^]^ It was important to control the impregnation of the silica sol or silica colloids so that the silica layer just covered the pore walls in the polymer polyHIPE, instead of filling the emulsion‐templated pores. Only weak physical interaction between the silica component and the polymer was present. Mahadik et al. prepared the polyHIPE from the oil monomer phase consisting of St, EHA, DVB, and 3‐(trimethoxysilyl)propyl methacrylate (TPM). A silica sol (prepared by hydrolysis of methyltrimethoxysilane) was then impregnated into the polyHIPE and covalently bonded to the polyHIPE via a condensation reaction. This silica aerogels‐laden polyHIPE network exhibited macroporous and mesoporous structures with high surface area of 468 m^2^ g^−1^, excellent bendability, high elasticity, superhydrophobicity (≈160°), low density (≈0.128 g cm^−3^), low thermal conductivity (≈0.045 W m^−1^·K^−1^), and good thermal stability (≈300 °C) in air (**Figure** **9**a,b).^[^ ^190^ ^]^

#### 2.2.2 Metal Oxide‐Polymer Hybrids

Due to high reactivity of metal alkoxides or other metal‐containing organic compounds, direct preparation of porous metal oxide‐polymer hybrids from metal organic precursors or co‐reaction with monomers has been rarely reported. The preparations have often relied on the use of metal oxide NPs as stabilizers or building blocks or the two‐step synthesis methodology. Different types of metal oxide‐polymer polyHIPE hybrids can be produced using similar methodologies, as discussed below. The choice of metal oxides depends on the desired properties of the target metal oxide‐polymer hybrids that can be exploited for intended applications.

The preparation of Fe_3_O_4_‐polymer polyHIPEs has been widely investigated because the composites can be readily separated or recycled by magnetic force among other useful properties. A hydrothermal process was used to prepare Fe_3_O_4_ NPs, which were subsequently modified with (3‐aminopropyl)triethoxysilane and *γ*‐MPS. Fe_3_O_4_ NPs‐stabilized Pickering O/W HIPEs were then prepared by the dropwise addition of *para*‐xylene into the Fe_3_O_4_ dispersed aqueous phase composed of CS, MBAM, AA, and co‐surfactant (Pluronic F‐68) (Figure 9c–f).^[^ ^192^ ^]^ The polymerization was completed by further addition of APS and TMEDA. Magnetic macroporous CS‐PAA hybrid polyHIPEs were obtained after washing by Soxhlet extraction, neutralization with aqueous NaOH solution, and drying.^[^ ^192^ ^]^ This method was further used to produce magnetic CS‐PAMPS porous composites^[^ ^193^ ^]^ and magnetic cellulose‐PAA porous beads via an O/W/O sedimentation polymerization approach.^[^ ^194^ ^]^ In a similar way, Fe_3_O_4_ NPs‐stabilized HIPEs were used to prepare magnetic porous PAM beads by O/W/O sedimentation polymerization.^[^ ^195^ ^]^

**Figure 10:**
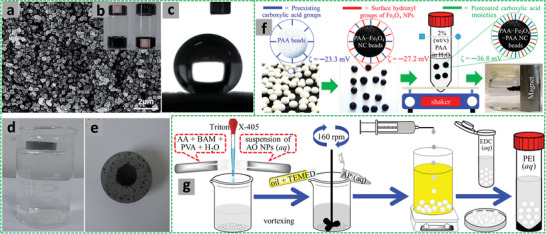
a) SEM image of Fe_3_O_4_ NPs and b) photograph of emulsions in the inset image; c) water contact angle of the PS foam; d) photograph of the PS foam on the water surface; and e) image showing adsorption of diesel by the PS foam. Adapted under a Creative Commons Attribution‐NonCommercial 3.0 Unported Licence.^[^ ^196^ ^]^ Copyright 2017, The author(s), published by Royal Society of Chemistry. f) Scheme for the preparation of PAA‐Fe_3_O_4_‐PAA composite beads. Reproduced with permission.^[^ ^103^ ^]^ Copyright 2019, American Chemical Society. g) Schematic view of the preparation of the PAA‐AO‐PEI (AO = alumina) NC beads. Reproduced with.^[^ ^201^ ^]^ Copyright 2021, American Chemical Society.

Fe_3_O_4_ NPs have also been dispersed into the oil phase of W/O HIPEs, which are then used as templates to fabricate porous hybrid materials. Jiang and co‐authors reported the preparation of magnetic hierarchically porous PS.^[^ ^196^ ^]^ Fe_3_O_4_ NPs were dispersed in the St/DVB phase (also containing Span 20 and AIBN) of the W/O HIPEs. The prepared composite foams exhibited a nano‐/microsized pore structure and good thermal stability, which was associated with the strong bonding between the Fe_3_O_4_ particles and the polymeric chains. Furthermore, the composite materials simultaneously showed hydrophobic, oleophilic, and magnetic properties (**Figure** **10**a–e).^[^ ^196^ ^]^ In the study reported by Zhu et al., magnetic porous polymers were prepared by dissolving/dispersing Span 80, AIBN, and amine‐functionalized magnetite (Fe_3_O_4_) NPs (50 nm) into the oil phase of W/O HIPEs in order to prepare magnetic porous P(St‐*co*‐DVB) polyHIPEs. The resulting magnetic porous polymers displayed hierarchical pores and a surface area of 5.53 m^2^ g^−1^.^[^ ^197^ ^]^

Mudassir et al. employed a two‐step (or post‐modification) method to fabricate hierarchically macroporous PAA−Fe_3_O_4_ beads.^[^ ^103^ ^]^ PAA polyHIPE beads were first prepared by O/W/O sedimentation polymerization and then used as the porous matrix for the in situ synthesis of Fe_3_O_4_ NPs by co‐precipitation. In turn, the Fe_3_O_4_ NPs offered diverse anchoring sites to immobilize another PAA layer, thereby providing higher overall negative charges. The PAA–Fe_3_O_4_–PAA beads exhibited hierarchical macropores in the size range of 0.38–38.93 µm and a BET surface area of 2.77 m^2^ g^−1^ (Figure 10f).^[^ ^103^ ^]^ Zowada and Foudazi soaked a PAMPS polyHIPE in aqueous FeCl_3_ solution and the swollen polyHIPE was then immersed in NaOH/NaCl solution, leading to the precipitation of Fe(OH)_3_. Further thermal treatment resulted in the formation of porous hydrated ferric oxide (FeOOH)‐PAMPS composites, which showed good performance in arsenate removal.^[^ ^198^ ^]^

Other metal oxide‐polymer polyHIPE composites can be prepare in a similar way, either via the one‐step Pickering emulsion or the two‐step post‐modification methods. For example, PEI‐bounded TiO_2_ NPs (together with Span 80) were used to stabilize W/O HIPEs, which were then employed as templates for the preparation of porous P(St‐*co*‐DVB)‐TiO_2_ NCs.^[^ ^199^ ^]^ Titania‐PS‐EGDMA polyHIPEs from W/O Pickering emulsion (TiO_2_ modified with OA),^[^ ^200^ ^]^ titania‐P(AM‐co‐AMPS) polyHIPEs from C/W HIPEs (TiO_2_ 5–10 nm, modified with silanes coupling agent),^[^ ^134^ ^]^ and titania‐PAM polyHIPEs from O/W Pickering emulsions (P25 TiO_2_, 20 nm, also with Tween 85),^[^ ^135^ ^]^ were also fabricated.

Mudassir et al. employed a one‐step method to prepare emulsion‐templated hierarchically macroporous PAA−AO NC beads by polymerizing the emulsion costabilized by the surfactant and AO NPs followed by their functionalization with branched PEI via a grafting‐to approach to synergistically improve the mechanical robustness and overall higher density of positive charge (over a certain pH range). The resultant PAA−AO−PEI beads exhibited hierarchical pores in the size range of 0.09–4.24 µm and a BET surface area of 5.42 m^2^ g^−1^ (Figure 10g).^[^ ^201^ ^]^

#### 2.2.3 Metal–Polymer PolyHIPEs

Porous polymer‐supported metal NPs are highly useful in enhancing mass transport, facilitating heterogeneous catalysis, and offering high recyclability.^[^ ^202^ ^]^ The common route is to form the polyHIPEs first followed by impregnation of metal ions solution. The metal ions are then reduced to generate metal NPs within the polyHIPE scaffold. For example, Backov and co‐authors prepared a series of organo‐Si(HIPE), which were then soaked in palladium(II) acetate [Pd(OAc)_2_] solution. Pd^2+^ ions were reduced to form Pd NPs within Si(HIPE) by NaBH_4_ under argon bubbling.^[^ ^203^ ^]^

**Figure 11:**
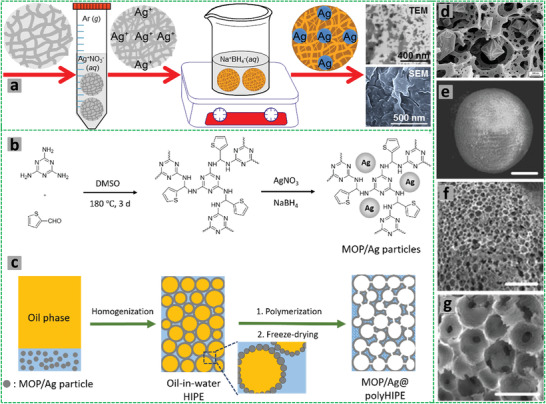
a) Schematics of the Ag NPs‐decorated emulsion‐templated hierarchically porous PVI beads, where the SEM image shows the pore surface morphology of the PVI beads and the transmission electron microscopy (TEM) image indicates the presence of Ag NPs. Reproduced with permission.^[^ ^102^ ^]^ Copyright 2017, American Chemical Society. Preparation of b) Ag‐incorporated melamine‐based microporous organic polymer (m‐MOP) particles and c) Ag‐incorporated m‐MOP particles‐decorated PAM monolith. Reproduced with permission.^[^ ^205^ ^]^ Copyright 2020, Elsevier. d) SEM image of Cu_3_(BTC)_2_MOF@polyHIPE. Reproduced with permission.^[^ ^208^ ^]^ Copyright 2008, Wiley‐VCH. SEM images show e) single bead view (scale bar = 500 µm), f) porous internal structure (scale bar = 200 µm), and g) porous internal structure (scale bar = 50 µm) of silica bead. Adapted with permission.^[^ ^182^ ^]^ Copyright 2003, Wiley‐VCH.

A range of Ag NPs‐polymer polyHIPEs have been prepared, particularly exploiting the anti‐bacterial properties of Ag NPs. Wang et al. prepared polystyrene sulfonate (PSS) polyHIPEs via Pickering HIPEs stabilized by halloysite nanotubes. The PSS polyHIPEs were impregnated with AgNO_3_ solution and then placed in boiling aqueous NaBH_4_ solution for reduction of Ag^+^ ions. The composite monoliths exhibited an open‐cell structure with interconnected pores, low density, and a surface area of 16.55 m^2^ g^−1^.^[^ ^204^ ^]^ Mudassir et al. used macroporous poly(1‐vinylimidazole) (PVI) beads to prepare the porous NCs with Ag NPs (**Figure** **11**a).^[^ ^102^ ^]^ The Ag^+^ ions‐loaded PVI beads were reduced in a boiling aqueous NaBH_4_ solution, which enhanced the swelling of porous PVI beads for easier penetration of the NaBH_4_ throughout the porous network to quickly and effectively reduce the Ag^+^ ions at the surface and interior of the beads, as indicated by the color of the intact and fractured beads. These hierarchically macroporous composite beads exhibited a BET surface area of 21.77 m^2^ g^−1^ and were stable for long time storage.^[^ ^102^ ^]^ The same group also prepared Ag‐poly(vinylsulfonic acid) (PVSA) beads via the chemical reduction of Ag^+^ ions using NaBH_4_ at RT without inert atmosphere. This approach produced Ag‐PVSA beads with hierarchical multimodal porosity (8.44 Å–28 µm) and small Ag NPs (down to ≈3.77 nm), which accounted for their decent BET surface area (197.74 m^2^ g^−1^).^[^ ^98^ ^]^

Lee and Chang employed Pickering O/W HIPEs stabilized by Ag‐incorporated particles for the preparation of Ag‐PAM porous composites.^[^ ^205^ ^]^ In the study, m‐MOP particles were prepared first via the reaction of melamine and thenaldehyde in DMSO at 180 °C. The m‐MOP particles were then dispersed in AgNO_3_‐DMF (dimethylformamide) solution and m‐MOP/Ag particles were obtained after reduction of Ag^+^ ions by NaBH_4_. The m‐MOP/Ag particles were dispersed in cyclohexane, which was then emulsified into aqueous solution containing AM, MBAM, and APS to form the O/W HIPE (Figure 11b,c). After polymerization and freeze drying, the prepared Ag‐PAM monoliths showed hierarchically pores (meso‐, micro‐, and interconnected macropores) with a BET surface area of 96 m^2^ g^−1^, also possessing water wettability and compressive properties.^[^ ^205^ ^]^

In addition to metal NPs‐polymer polyHIPE composites, Brun et al. prepared a series of organo‐Si(HIPE) materials and used them to obtain lanthanide‐loaded hybrid monoliths by directly imbibing Eu(NO_3_)_3_ solution into the porous Si(HIPEs) followed by drying. The Eu^3+^‐loaded hybrid monoliths exhibited luminescence with both macro‐ and microporosity and surface areas in the range of 150–360 m^2^ g^−1^.^[^ ^206^ ^]^

#### 2.2.4 MOF–Polymer PolyHIPE Hybrids

The objective of preparing MOF‐polymer polyHIPE hybrids is to combine the excellent control of microporosity and rich chemistry of MOFs and the highly interconnected macroporosity of polyHIPEs and optimize the properties of brittle and crystalline MOFs and flexible and malleable polymers. MOFs are regarded as microporous (and can also be mesoporous) crystalline materials synthesized by the bonding of metal ions and organic ligands. The huge variety of available metal ions and designed organic ligands make it truly attractive to produce MOFs and MOF/polymer composites with desired properties.^[^ ^191^, ^207^ ^]^ However, in the area of making MOF‐polymer polyHIPE hybrids, most of the researchers have focused on the use of easily prepared and relatively water‐stable MOF NPs, for example, HKUST‐1, UiO‐66, ZIF‐8 (ZIF is the acronym for zeolitic imidazolate framework), etc.

The preparation of MOF‐polymer polyHIPE composites has been achieved with the 2‐step preparation method that involves preparation of polymer polyHIPEs first and then synthesis of MOFs within the emulsion‐templated pores of the polyHIPEs. Schwab et al. prepared a polyHIPE monolith composed of VBC crosslinked with DVB in which the Cu(NO_3_)_2_.3H_2_O and 1,3,5‐benzenetricarboxylic acid (H_3_BTC) solution in ethanol/water was impregnated into the polyHIPE. A solvothermal reaction was then performed to produce Cu_3_(BTC)_2_ MOF inside the macroporous monolith. Notably, the resulting composites exhibited a high BET surface area of 570 m^2^ g^−1^ and a pore volume of 0.38 cm^3^ g^−1^ (Figure 11d).^[^ ^208^ ^]^ Likewise, the Cu_3_(BTC)_2_ MOF@PAM composite beads were prepared by a simple soaking and the solvothermal method. The prepared composites displayed hierarchical macro‐ and microporosity with improved mechanical stability and a surface area of 654 m^2^ g^−1^.^[^ ^209^ ^]^ By introducing Fe_3_O_4_ NPs into the continuous monomer phase, magnetic PS and PAM polyHIPEs were prepared. Both of them were then used to fabricate magnetic MOF‐polymer polyHIPE hybrids with a range of MOFs including ZIF‐8, UiO‐66, Cr‐MIL‐101 (MIL stands for Matérial Institut Lavoisier), HKUST‐1, and Fe‐MIL‐101‐NH_2_.^[^ ^210^, ^211^ ^]^

Another effective route is to use MOF NPs‐stabilized HIPEs as templates. UiO‐66 NPs have been found to be highly effective in forming O/W HIPEs.^[^ ^212^ ^]^ However, direct freezing drying of UiO‐66‐stabilized cyclohexane‐in‐water emulsions resulted in collapsed structures. The incorporation of monomers (e.g., AM) or polymer (e.g., PVA, as both co‐stabilizer and adhesive) could generate hierarchically porous UiO‐66‐polymer monoliths.^[^ ^212^, ^213^ ^]^ The inclusion of PVA during the preparation could reduce emulsion‐templated pores from 150 to about 25 µm.^[^ ^213^ ^]^ UiO‐66 NPs (with PVA as co‐stabilizer) were also used to form C/W HIPEs for preparing porous UiO‐66/PAM monoliths.^[^ ^142^ ^]^ Other MOF such as ZIF‐8 NPs were also used to stabilize O/W HIPEs and produce ZIF‐8‐PS microcapsules.^[^ ^214^ ^]^ HKUST‐1 particles were employed to generate HKUST‐1‐PAM monoliths via C/W HIPE templating.^[^ ^141^ ^]^ Fe_3_O_4_@HKUST‐1 core–shell particles were synthesized and then used to prepare magnetic MOF‐polymer polyHIPE adsorbents.^[^ ^215^ ^]^ Ni‐1,4‐dicarboxybenzene MOF NPs were used as stabilizers to form IL‐in‐water HIPEs, which were then used as template to obtain porous MOF‐PAM composites.^[^ ^216^ ^]^

Instead of using simple MOF NPs, Zhu et al. included OA‐modified CuO NPs in the preparation of PGMA polyHIPE.^[^ ^217^ ^]^ This polyHIPE was then impregnated with H_3_BTC solution in ethanol/water and HKUST‐type MOF particles were formed in situ after reaction at 120 °C. PEI functionalization was then achieved via epoxy‐amine reaction and amine‐metal interaction. This material displayed good thermal stability and CO_2_ capture from simulated flue gas.^[^ ^217^ ^]^

Attempts were also made to produce MOF‐polymer polyHIPEs in one step, that is, including both monomers and MOF precursors in the HIPEs. It has been proven to be highly challenging to form stable HIPEs. After many trials, Shirshova and co‐authors found that stable HIPEs could be prepared from stearyl methacrylate (SMA, monomer)/1,6‐hexanediol dimethacrylate (HDDMA, crosslinker)/Arlacel P135 (a triblock copolymer as surfactant) as the continuous phase and Cu(NO_3_)_2_.2.5H_2_O and H_3_BTC in DMF‐ethanol‐water (1:1:1 v/v) solution as the internal phase. The simultaneous formation of MOF particles and thermal polymerization of HIPE was able to produce the MOF‐polymer polyHIPE in one step. However, in that case, the surface area was quite low at 16 m^2^ g^−1^.^[^ ^218^ ^]^

#### 2.2.5 Other PolyHIPE Hybrids

Other inorganic‐polymer polyHIPEs or emulsion‐templated hybrids can be prepared by employing similar methodologies. For example, Na‐montmorillonite (MMT) was added into the aqueous phase (also containing SCMC, AM, and Pluronic F‐68) of a MIPE as co‐stabilizer and inorganic building blocks. The eco‐friendly inolenic acid was the internal oil phase. After polymerization, the resulting hybrid material was washed with acetone by Soxhlet extraction, and subsequently placed in the NaOH‐containing ethanol/water (7:3) solution in order to convert amides group to carboxyl groups. This was followed by dehydration with absolute alcohol in the presence of zeolite 3A. An interconnected macroporous structure was observed for the dry composite material.^[^ ^219^ ^]^ Plate‐like aminoclay NPs with adsorbed glucose were used to stabilize O/W emulsions with other co‐surfactants including SDS, CTAB, and Pluronic F‐127. 1,4‐butanediol diglycidyl ether was then added to crosslink the amine groups on aminoclay particles. It was found that the use of Pluronic F‐127 produced elastic and soft monoliths with interconnected macropores. The porosity could also be tuned by varying the volume fraction of oil phase while the encapsulated enzymes remained stable during preparation with chaotropic solvent and variation of pH.^[^ ^220^ ^]^

Although it is not covered in this review, the methods described above may also be employed for the preparation of organic–organic porous hybrids, with two organic components showing very different properties. For example, PS polyHIPE‐based membranes were infused with low surface tension poly(dimethylsiloxane) (PDMS) to produce slippery liquid‐infused porous surface systems, which exhibited very good self‐cleaning and self‐repairing properties.^[^ ^171^ ^]^ In another study, Zhang et al. employed O/W HIPEs by introducing heptadecafluorodecyl methacrylate into the oil (toluene) phase, which was polymerized at the water/oil interface to prepare porous PAM. This method produced a porous material with hydrophilic and oleophobic properties.^[^ ^138^ ^]^

#### 2.2.6 Summary

Section 2.2 describes the preparation of emulsion‐templated porous hybrid and composite materials such as Si‐containing hybrids using bridged and polyfunctionalized precursors (e.g., silsesquioxane) and silica‐organic hybrids, nanosized metals (e.g., Pd, Ag, etc.) and metal oxides/hydroxides (i.e., Fe_3_O_4_, FeOOH, TiO_2_, etc.) containing polyHIPEs, MOF (HKUST‐1, MOF‐2, Fe‐MIL‐101, UiO‐66, etc.) incorporated polyHIPEs, and other polyHIPEs including MMT and aminoclay‐based composites. Many research groups proposed different emulsion templating routes to design and fabricate a wide array of advanced organic–inorganic hybrid platforms and composite systems with their prospects from laboratory to market. Of these, the one‐step synthesis involves mixing of both organic and inorganic precursors or using inorganic NPs as stabilizers to form Pickering emulsions, whereas two‐step procedures correspond to the preparation of host emulsion‐templated porous polymers followed by in situ generation (i.e., sol–gel hydrolysis, coprecipitation, solvothermal reaction, etc.) or incorporation of guest inorganic components. Many presented reports mainly emphasized to control density, porosity (volume, size, and structure), surface area, composition, surface chemistry, and to develop shape, morphology, and size of such porous emulsion‐templated organic‐inorganic composites. However, the total control over design‐led synthesis of emulsion‐templated porous hybrids and composites to obtain desired features and optimization of their physical properties for appropriate utilization is still a matter of substantial interest. The typical preparation recipes and porosity properties of the polyHIPE hybrid/composite materials discussed in Section 2.2 are summarized in **Table** **2**.

**Table 2:** Summary of preparation methods and main properties of emulsion‐templated porous organic−inorganic hybrids/composites

Materials	Preparation	Porosity/Property^a)^	Ref.
Silsesquioxane‐polymer	W/O HIPEs, stabilizer K_2_SO_4_, EHA/DVB/VSQ/MSQ in continuous phase Polymerized at 65 °C, 24 h.	Density: 0.10–0.16 g cm^−3^	[177]
Silsesquioxane‐poly(2‐ethylhexyl acrylate)	W/O HIPEs, stabilizer K_2_SO_4_, EHA/DVB/VSQ or POSS in continuous phase	Improved thermal stability Better reinforcement with POSS	[180]
Silica‐poly(*tert*‐butyl methacrylate‐divinylbenzene) [P(*t*BMA‐DVB)]	W/O emulsion, 80% water phase TEOS introduced either oil or water phase Ferrocene derivative of cholesterol as stabilizer	Better pore structure when TEOS in oil phase Crush strength: up to 5.5 MPa Density: 0.12–0.29 g cm^−3^	[181]
Organo‐Si(HIPE)	O/W emulsions, TEOS, and alkoxysilanes hydrolyzed in aqueous phase first; Dodecane as the internal phase, tetradecyltrimethylammonium bromide as surfactant	Pore volume: up to 13.7 cm^3^ g^−1^ Bulk density: 0.07–0.27 g cm^−3^ Surface area: 53–450 m^2^ g^−1^	[183]
Silica‐P(St‐*co*‐DVB)	W/O emulsion stabilized by OA‐modified silica NPs and Hypermer 2296, internal phase 70–85%	Gas permeability adjusted Changed mechanical stability	[185]
Silica‐P(St‐*co*‐DVB)	W/O emulsion, with silica NPs and n‐OTES in the oil phase Acidified cotton as stabilizer	Density: 0.06–0.16 g cm^−3^ Surface area: <10 m^2^ g^−1^	[187]
Organo‐silica foam	W/O emulsion, PEOS in toluene as stabilizer AEAPS or GPTMS or AA/MBAM in the water phase Functionalized with amine, epoxy groups or PAA	Hierarchical pores Surface area: 450, 481, 500 m^2^ g^−1^ for amino‐, epoxy‐, and carboxyl‐functionalized samples, respectively	[172]
Silica‐P(St‐DVB‐EHA)	W/O emulsions, Span 80, and TPM also in the oil phase Silica sol prepared from MTMS Covalent bonded modification of polyHIPEs with silica sol	Macroporous and mesoporous structure Surface area: 115‐468 m^2^ g^−1^ Water contact angle: 160^o^–165° Density: 0.128–0.142 g cm^−3^	[190]
Fe_3_O_4_‐CS‐PAA	Functionalized Fe_3_O_4_ NPs‐stabilized O/W Pickering HIPEs CS, AA, MBAM, and F‐68 in the water phase Similar approach used to prepare composites with CS‐PAMPS, cellulose‐PAA beads and PAM beads	Interconnected macroporous structure maintained	[192, 193, 194, 195]
Fe_3_O_4_‐P(St‐*co*‐DVB)	Fe_3_O_4_ NPs‐stabilized W/O HIPEs, with Span 20 or Span 80 as co‐stabilizer	Hierarchical pores Surface area: 5.5 m^2^ g^−1^	[196, 197]
Fe_3_O_4_‐PAA beads	PAA beads prepared first by O/W/O sedimentation polymerization Fe_3_O_4_ NPs then formed in PAA	Macropores: 0.38–39 µm Surface area: 2.77 m^2^ g^−1^	[103]
FeOOH‐PAMPS	PAMPS polyHIPEs prepared first Precipitation of Fe(OH)_3_ by FeCl_3_ and NaOH inside the polyHIPEs Thermal treatment (50 °C, 1 h) to convert Fe(OH)_3_ to FeOOH.	Interconnected pores: 9–30 µm Surface area: 2.65 m^2^ g^−1^	[198]
Al_2_O_3_‐PAA‐PEI	Al_2_O_3_‐PAA beads prepared via surfactant‐aided Pickering emulsion templating Functionalization with PEI via grafting‐to approach	Macropores: 0.09–4.24 µm Surface area: 5.42 m^2^ g^−1^	[201]
TiO_2_‐P(St‐*co*‐DVB)	W/O HIPEs stabilized by PEI‐modified TiO_2_ NPs and Span 80, 90% internal phase	CO_2_ adsorption	[199]
TiO_2_‐P(St‐EGDMA)	W/O emulsion stabilized by OA‐modified titania NPs, up to 85% internal phase	Porosity: up to 86%, bulk density" 0.16 g cm^−3^; compression modules: 42–65 MPa for polyHIPEs	[200]
TiO_2_‐P(AM‐*co*‐AMPS)	PVA & TiO_2_ NPs‐stabilized C/W HIPE, AM as monomer, CO_2_ removed by depressurization.	Pore size adjusted by CO_2_ density and TiO_2_ amount; presence of –SO_3_^−^ group	[134]
TiO_2_‐PAM	P25 TiO2‐stabilized O/W emulsion Tween 85 as co‐stabilizer Paraffin as internal phase	Voids sizes: 4.2–11.1 µm for surfactant > 1.0 wt% Interconnectivity up to 27.8%	[135]
Pd@organo‐Si(HIPE)	Pd(OAc)_2_ solution impregnated into organo‐Si(HIPE); Reduction to Pd NPs by NaBH_4_ under Ar	Surface area: 300–400 m^2^ g^−1^ Pore volume: 8.8–10.7 cm^3^ g^−1^ Density: 0.1–0.14 g cm^−3^ Catalyze Heck coupling reactions	[203]
Ag@PSS	AgNO_3_ solution impregnated into PSS polyHIPEs; Reduced in boiling aqueous NaBH_4_ solution	Low density Surface area: 16.55 m^2^ g^−1^	[204]
Ag@PVI beads	AgNO_3_ solution impregnated into PVI beads Reduced in boiling aqueous NaBH_4_ solution	Hierarchical macroporosity Surface area: 21.77 m^2^ g^−1^	[102]
Ag@PVSA beads	AgNO_3_ solution impregnated into PVSA beads Reduced at RT in NaBH_4_ solution	Hierarchical multimodal porosity: 8.44 Å–28 µm Surface area" 198 m^2^ g^−1^	[98]
Ag@m‐MOP@PAM	m‐MOP NPs complexed with Ag^+^ and then reduced by NaBH_4_ in DMF Ag@m‐MOP NPs‐stabilized O/W HIPEs	Surface area: 96 m^2^ g^−1^ Macropores: 30–40 µm Meso‐, micro‐, and interconnected macropores	[205]
Cu_3_(BTC)_2_@P(VBC‐DVB)	P(VBC‐DVB) prepared and then hydrophilized by 3‐amino‐1‐propanol in DMF Cu(NO_3_)_2_.3H_2_O/H_3_BTC/ethanol/water solution impregnated Solvothermal reaction at 403.2 K, 12 h.	Pore volume:38 cm^3^ g^−1^ Surface area: 570 m^2^ g^−1^	[208]
HKUST‐1@PAM beads HKUST‐1@Oxide beads	Cu(NO_3_)_2_.3H_2_O/H_3_BTC/ethanol/water solution impregnated Solvothermal reaction at 120 °C, 12 h.	Hierarchical macro‐ and microporosity Surface area: 654 m^2^ g^−1^	[209]
HKUST‐1@PAM	Magnetic Fe_3_O_4_‐PAM polyHIPEs used as hosts; MOF precursor solutions impregnated Solvothermal reactions performed	Surface area: 701 m^2^ g^−1^	[211]
MOF‐2@PAM		Surface area: 68 m^2^ g^−1^	
UiO‐66@PAM		Surface area: 202.0 m^2^ g^−1^	
Fe‐MIL‐101@PAM		Surface area: 523 m^2^ g^−1^	
Fe‐MIL‐101‐NH_2_@PAM		Surface area: 581 m^2^ g^−1^	
UiO‐66 and composite polyHIPEs	Freeze‐drying of UiO‐66‐stabilized cyclohexane‐in‐water emulsion Adding PVA or AM (and polymerization) to improve stability	Low density: 0.012 g cm^−3^ 23.3–27.2% UiO‐66 loading	[212, 213]
UiO‐66@PAM monoliths	PVA & UiO‐66 NPs‐stabilized C/W HIPE, AM & AMPS as monomers, CO_2_ removed by depressurization	Hierarchically macroporous structure maintained, pore diameter 10–50 µm	[142]
HKUST‐1@PAM monoliths	PVA & HKUST‐1 NPs‐stabilized C/W HIPE, AM & AMPS as monomers, CO_2_ removed by depressurization	Surface area: 10 m^2^ g^−1^; Bulk density: 0.14 g cm^−3^	[141]
HKUST‐1@PGD	CuO‐PGD composites prepared first, conversion of CuO to HKUST‐1, further modified by PEI	Surface area: 620 m^2^ g^−1^ before and 96 m^2^ g^−1^ after PEI modification	[217]
HKUST‐1@polyacrylate	One step HIPE polymerization External phase including SMA, HDDMA, and Arlacel P135 as stabilizer Internal phase of Cu(NO_3_)_2_2.5H_2_O and H_3_BTC in DMF‐ethanol‐water (1:1:1 v/v) Very difficult to form stable emulsions	Surface area: 16 m^2^ g^−1^	[218]
MMT‐carboxymethyl cellulose (CMC)‐PAM	O/W emulsions stabilized by MMT and Pluronic F‐68 CMC, AM, and MBAM in water phase	Emulsion‐templated pore structure	[197]
Aminoclay composites	Glucose‐adsorbed aminoclay nanoplates used to stabilize O/W emulsion SDS, CTAB, or Pluronic F‐127 as co‐surfactant 1,4‐butanediol diglycidyl crosslinking aminoclay	Porosity up to 84% Interconnected macroporous structure	[220]

^a)^ Surface area values are for BET surface areas and pore volumes are measured by Hg intrusion porosimetry. Bulk density data are always quoted here for “density”.

### 2.3 Inorganic Structures

The fascination in design‐led synthesis of emulsion‐templated porous organic–inorganic hybrids/composite has recently received significant attention in advanced functional materials science, though the issues pertaining to the low conductivity, adsorptive nature, and poor stability of organic polymers undermine their use especially for the photocatalytic applications. Nonetheless, the shaping on macroscopic level is a matter of considerable interest on account of the difficult operation and complicated processability of nano/microscale inorganic materials.^[^ ^101^, ^221^ ^]^ In this regard, the emulsion templating has emerged as a promising approach to fabricate purely inorganic structures with hierarchical porosity, high surface area, and high thermal and mechanical stability. The following subsections demonstrate a detailed review of the existing literature on the preparation methods of state‐of‐the‐art purely inorganic materials.

#### 2.3.1 Silica‐Based Beads

As a molecular precursor, TEOS was hydrolyzed and then mixed with AM and MBAM solutions. An O/W HIPE was prepared with mineral oil as the internal phase. Porous PAM‐silica beads were prepared via an O/W/O sedimentation polymerization process.^[^ ^182^ ^]^ The composite beads were calcined at 520 °C in air to generate porous silica beads. Small shrinkage was observed after the calcination. However, both the bead shape and highly interconnected macroporous structure were retained (Figure 11e–g). These silica beads showed macropores of around 5 µm, mesopores of around 10 nm, a surface area of 422 m^2^ g^−1^, and a high pore volume of 5.8 cm^3^ g^−1^.^[^ ^182^ ^]^

In another study, porous PAM beads were immersed in a pre‐prepared TEOS sol. The soaked beads were taken out and a sol–gel process was allowed to occur in air at RT to produce porous PAM‐silica beads. The calcination of the composite beads led to the formation of emulsion‐templated porous silica with mesopores <3 nm and the surface area up to 640 m^2^ g^−1^.^[^ ^188^ ^]^ Surfactant templating (e.g., triblock copolymers poly(ethylene glycol)‐*block*‐poly(propylene glycol)‐*block*‐poly(ethylene glycol) (PEG‐*b*‐PPG‐*b*‐PEG) could be introduced into the TEOS sol to adjust the mesopore size and porosity of the silica. In this regard, the two‐step impregnation method is superior to the method directly employing molecular silica precursor.^[^ ^182^, ^188^ ^]^ A generic method that can be used to produce hierarchically porous Al_2_O_3_, TiO_2_, and ZrO_2_ beads simply involves the immersion of polyHIPE polymers into other metal oxide precursor sols.^[^ ^188^ ^]^ This method has been extended by replacing silica or metal oxide sols with relevant NPs suspensions. For example, porous silica beads were prepared by simply immersing PAM beads into the silica colloid solution to prepare porous silica‐PAM beads. A PEG‐*b*‐PPG‐*b*‐PEG triblock copolymer was further used to induce additional mesoporosity. The calcination of these composite beads generated porous silica beads.^[^ ^189^ ^]^ It was also possible to immerse the silica‐PAM beads in other metal oxide precursors. Furthermore, the porous hybrid inorganic beads such as silica‐titania and silica‐AO could be readily produced by using similar procedures.^[^ ^189^ ^]^

**Figure 12:**
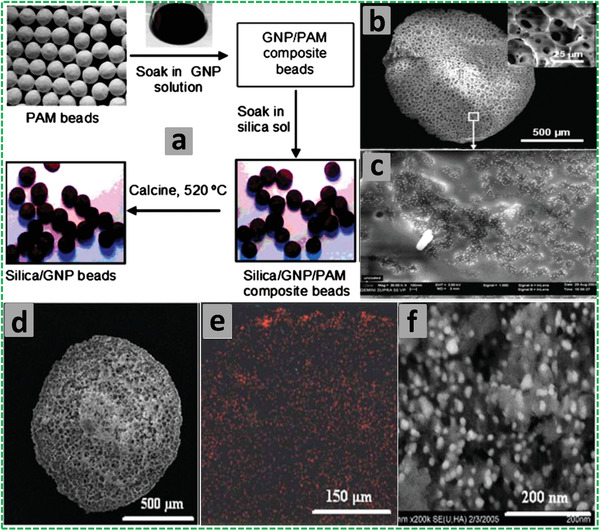
a) Schematic illustration for the step‐wise preparation of hierarchically porous GNP–silica composite beads. SEM images of GNP–silica composite beads showing b) porous structure and interconnected emulsion‐templated pores, and c) distribution of GNPs over silica surface. d) SEM image (cross‐sectional view) and e) energy‐dispersive X‐ray map of AO–GNP composite bead, and f) distribution of GNPs over AO surface. Adapted with permission.^[^ ^222^ ^]^ Copyright 2006, Royal Society of Chemistry.

Additional components or functionalities may be introduced to silica polyHIPEs during the multiple‐step preparation. Zhang et al. reported the preparation of hierarchically porous site‐isolated gold NPs (GNPs)‐silica beads.^[^ ^222^ ^]^ By soaking PAM beads in aqueous sodium acrylate‐stabilized GNPs, GNPs were adsorbed onto the pore walls of PAM, facilitated by the amine groups on PAM. The GNPs‐PAM beads were then immersed in TEOS sol, kept in a freezer at −20 °C overnight, incubated at RT for 12 h, and dried in vacuum oven at 60 and 120 °C each for 24 h in order to complete the sol–gel processing of the silica. After calcination, hierarchically porous GNP–silica beads with a BET surface area of 383 m^2^ g^−1^ were produced (**Figure** **12**a–c). Similarly, the hierarchically porous GNPs−AO beads could also be prepared (Figure 12d–f).^[^ ^222^ ^]^

#### 2.3.2 Metal Oxide Beads and Monoliths

Emulsion‐templated porous TiO_2_ has been fabricated using both precursor sols and NPs as building blocks. TiO_2_ precursor sol can be easily impregnated into a polymer polyHIPE, thereby generating porous TiO_2_‐polymer composite.^[^ ^101^, ^188^ ^]^ Mudassir et al. prepared clear titanium isopropoxide (TIP) sol by hydrolyzing with acetic acid. After impregnating PAM beads with the TIP sol, the nanoscale TiO_2_ building units were progressively nucleated, grown, and hierarchically organized during the sol–gel processing. The shape and pore structure of the polyHIPE beads were retained after template removal by calcination. The combination of the hierarchical macroporosity, crystalline anatase nature, nanoscale feature, and relatively high surface area (41.16 m^2^ g^−1^) makes these TiO_2_ beads highly effective in photodegradation of organic dyes and microbes.^[^ ^101^ ^]^

TiO_2_ NPs and poly(*N*‐isopropylacrylaimde‐*co*‐methacrylic acid) P(NIPAM‐*co*‐MAA)‐based microgels were used as stabilizer to prepare a stable Pickering O/W HIPE consisting of hexane and water. This Pickering HIPE was freeze‐dried to remove hexane and water. The sintering of the resultant material at 600, 700, 800, and 1200 °C removed microgel particles and caused fusion of TiO_2_ NPs, thereby producing a mechanically stable and hierarchically porous titania material. The resultant titania displayed interconnected macropores (30–72 µm) with throats (1.9–9 µm) as well as the microgel‐templated nanopores (≈100 nm).^[^ ^223^ ^]^ Koler and Krajnc prepared macroporous TiO_2_ monoliths via W/O HIPE templating by using the monomers, TiO_2_ particles (200 nm), and surfactant in the continuous phase. Polyacrylate‐titania polyHIPE monoliths were produced and then calcined at 1150 °C to generate 3D interconnected cellular monoliths with macropores of 70–100 µm connected by channels.^[^ ^224^ ^]^

To prepare AO polyHIPEs, a W/O HIPE was first prepared with alumina particles (290 nm), monomers, and a photoinitiator in toluene as the continuous phase. The resulting emulsion was transferred into a silicone mold, polymerized in a UV chamber, washed with ethanol, and dried under vacuum at 40 °C for 24 h. The composite monolith was sintered in air at 1400 °C (0.5 °C min^−1^) to obtain a macroporous AO of cellular interconnected morphology with cavities of ≈15 µm.^[^ ^225^ ^]^

**Figure 13:**
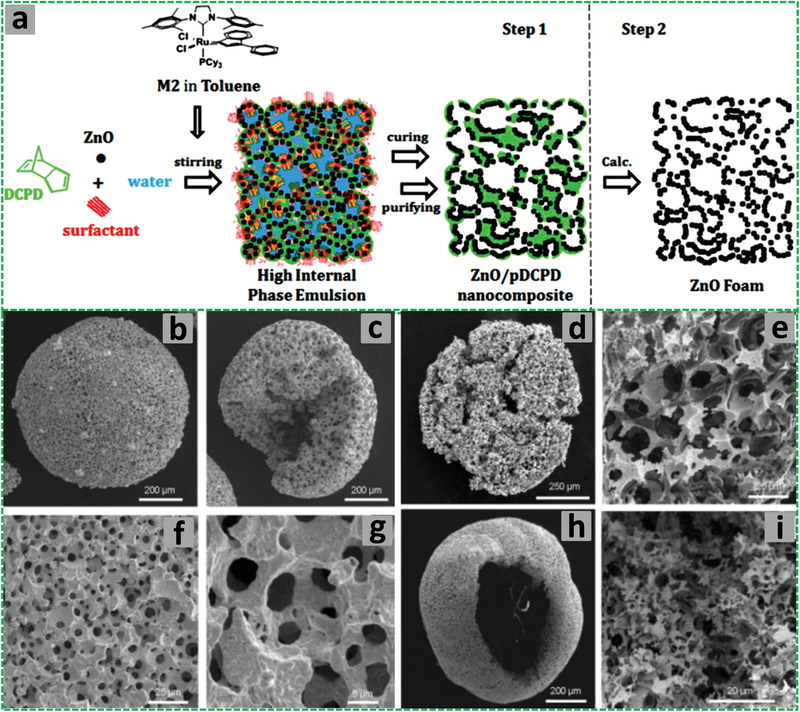
a) Schematic illustration for the preparation of ZnO/PDCPD NCs and macroporous ZnO Foams. Reproduced with permission.^[^ ^226^ ^]^ Copyright 2014, American Chemical Society. SEM images of b) a single porous gold bead prepared by using GNPs as the building blocks and c) its cross‐sectional and d,e) surface views showing the pore structures. SEM images of f) a single porous gold bead (cross‐sectional view) prepared via in situ reduction of HAuCl_4_ and g) its internal pores structure. h) Hollow gold bead resulted from the calcination of non‐uniformly distributed gold‐loaded porous polymer bead and i) its internal pores structure. Adapted with permission.^[^ ^228^ ^]^ Copyright 2004, Wiley‐VCH.

Macroporous ZnO foams were prepared via OA‐functionalized ZnO NPs‐stabilized HIPE templating. The Pickering emulsion was prepared by mixing dicyclopentadiene (monomer), ZnO NPs (stabilizer), Pluronic L‐121 (co‐stabilizer), and toluene. An initiator was added into the emulsion prior to its transfer into a mold. Curing the emulsion at 80 °C produced white solid monolith ZnO–poly(dicyclopentadiene) (PDCPD) monolith, which after calcination at 550 °C produced a needle‐like or sea urchin‐like ZnO foam that exhibited a specific surface area of 5.5 m^2^ g^−1^ (**Figure** **13**a).^[^ ^226^ ^]^

#### 2.3.3 Metallic Materials

Porous metals are useful for orthopedics, catalysis, energy storage, and electrochemical applications.^[^ ^17^, ^227^ ^]^ Purely metal materials with highly interconnected porosity and improved mass transport are highly promising for these applications. Zhang et al. reported the synthesis of emulsion‐templated porous gold beads by using GNPs as the building blocks through two different routes.^[^ ^228^ ^]^ In route 1, the emulsion‐templated PAM beads were soaked in aqueous sodium acrylate‐stabilized GNPs dispersion (average particle size = 15 nm) to prepare PAM–gold composites. The subsequent drying followed by calcination produced emulsion‐templated gold beads (Figure 13b–e). In route 2, the emulsion‐templated PAM beads were soaked in aqueous solution of HAuCl_4_ followed by in situ reduction of Au^3+^ ions using a freshly prepared aqueous NaBH_4_ solution. The subsequent drying and calcination generated highly porous gold beads. The core of these beads was hollow because of the non‐uniform distribution of gold. However, the surface of gold bead prepared via the second route was relatively more porous (Figure 13f–i).^[^ ^228^ ^]^ The affinity of amine groups on PAM ensured a high loading of GNPs within porous PAM, which was essential for the production of porous gold beads.

#### 2.3.4 Summary

We herein reviewed some state‐of‐the‐art porous purely inorganic designs such as SiO_2_, TiO_2_, Al_2_O_3_, ZrO_2_, ZnO, Au as well as SiO_2_‐TiO_2_, SiO_2_‐Al_2_O_3_, Au‐SiO_2_, and Au‐Al_2_O_3_ beads and monoliths with hierarchically organized interconnected micro‐, meso‐, and macropores, nanoscale features, high surface areas, crystalline nature, and millimeter sizes depending upon the nature, structure, and functionality of the building units/blocks. The discussion under Section 2.3 presented the preparations of well‐defined porous inorganic architectures involving the impregnation, controlled reactivity, organization, and assembly of inorganic molecular precursors (e.g., metal alkoxide, inorganic salts, etc.), inorganic NPs, or hybrid network (e.g., MOFs or organometallic compounds, etc.) into the emulsion‐templated porous polymeric scaffolds for producing organic‐inorganic hybrids/composites followed by removal of organic phase often via calcination to finally achieve fascinating inorganic structures of the desired properties with controlled size, shape, and tailored morphology as that of the template. The controlled generation of the monodispersed building blocks inside of the porous template typically allows greater control over the structure and properties of resulting inorganic structures. **Table** **3** summarizes the preparation and properties of the inorganic structures.

**Table 3:** Summary of preparation methods and properties of inorganic polyHIPEs

Materials	Preparation	Porosity/Property^a)^	Ref.
Silica beads	TEOS sol included in the continuous phase of O/W emulsion Silica‐PAM beads prepared by the O/W/O sedimentation polymerization Calcine the composite beads at 520 °C.	Bead size: 1.34 mm Pore volume: 5.68 cm^3^ g^−1^ Surface area: 422 m^2^ g^−1^	[182]
SiO_2_, AO, TiO_2_, and ZrO_2_ beads	PAM polyHIPE beads immersed in various precursor sols; Surfactants incorporated in the sols for more mesopores Sol‐gel process in air at RT to generate the composite beads Calcination at 520 °C to produce inorganic beads	Interconnected micropores (in silica beads), mesopores and large macropores (5–10 µm)	[188]
Silica, Silica−AO and silica−titania hybrid beads	PAM or PAM composite beads immersed in silica colloidal suspensions Drying and calcining at 520 °C for porous inorganic beads	Surface area: up to 197 m^2^ g^−1^	[86]
GNPs−silica and GNPs−AObeads	GNPs adsorbed on PAM beads, then soaked in silica sol or AO sol Calcine the composite beads at 520 °C	Combination of macropores (>5 µm) and interconnected mesopores (2–10 nm) Surface area: 383 m^2^ g^−1^ (gold–silica beads) Presence of discrete GNPs	[222]
TiO_2_ beads	PAM beads impregnated with the precursor sol Sol–gel and calcination at 520 °C	Hierarchical macroporosity with crystalline anatase Surface area: 41 m^2^ g^−1^	[101]
TiO_2_	Freeze‐drying of hexane‐in‐water emulsions stabilized by TiO_2_ NPs and P(NIPAM‐*co*‐MAA) microgels Sintering at 600–1200 °C Lower surface area and higher crystalline anatase phase with higher sintering temperature	Pore volume: 5.1–6.2 mL g^−1^ Surface areas: 4.88–28.78 m^2^ g^−1^ Density: 0.15–0.18 g mL^−1^	[223]
TiO_2_	TiO_2_‐polyacrylates prepared from W/O emulsions with TiO_2_ NPs in the oil phase Calcined at 1150 °C	Interconnected cellular structure with pores (70–100 µm)	[224]
AO	AO‐polyacrylates prepared from W/O emulsions with AO particles and photoinitiator in the oil phase Polymerized by UV irradiation Calcined at 1400 °C	Interconnected cellular morphology with voids of ≈15 µm	[225]
ZnO	ZnO‐PDCPD prepared from O/W emulsions stabilized by ZnO NPs and Pluronic L‐121 Calcined at 550 °C	Surface area: up to 5.5 m^2^ g^−1^	[226]
Gold beads	GNPs‐PAM beads prepared by direct adsorption of GNPs in PAM or by soaking PAM beads with HAuCl_4_ solution and subsequent reduction via the use of NaBH_4_ Calcined at 520 °C in air	Bead size: 0.5–1 mm Average macropores: ≈4 µm Density: 1.19 g cm^−3^ Surface areas: <5 m^2^ g^−1^	[228]

^a)^ Surface area values are for BET surface areas and pore volumes are measured by Hg intrusion porosimetry. Bulk density data are always quoted here for “density”.

### 2.4 Emulsion‐Templated Porous Carbon/Graphene‐Based Materials

Carbonaceous materials such as carbon nanotubes (CNTs), carbon aerogels, activated carbons, and graphene are promising candidates in a myriad of applications including electromagnetic interference shielding, electrodes in energy storage devices, heat transfer, catalyst supports, and water purification on account of their chemical inertness, excellent physicochemical stability, good thermal/electrical conductivity, high pore volume, hierarchical porosity, pores interconnectivity, high specific surface area, low density, biocompatibility, and low cost. These porous carbonaceous materials have been prepared via hard and soft templating routes.^[^ ^229^, ^230^, ^231^, ^232^ ^]^

One approach uses carbonization of polymerized St‐based HIPE to produce porous carbons with nanometer size pores in the walls resulting from densification during carbonization, but this method does not actively template mesopores. Another strategy involves agglomerated colloids of a carbonaceous precursor to produce hierarchical macroporous foam, whereas the surfactant templating in the colloids produces mesopores. In another method, a resin is polymerized around a silica colloid or micrometer‐sized polymer and subsequently carbonized to form hierarchical carbon. The pore size of the resultant carbonaceous materials depends upon the colloid diameter. For instance, use of colloidal crystals often produces macropores, whereas arranging smaller colloids around colloidal crystals is used to induce an additional level of mesoporosity. However, the usage of hydrofluoric acid (HF)‐based etching hinders the applicability of solid colloids‐based approach.^[^ ^23^, ^233^ ^]^

Nonetheless, the emulsion templating that uses inexpensive and easily removable templating materials has appeared as a promising approach to synthesize carbonaceous porous materials with hierarchically structured interconnected macropores, mesopores, and micropores.^[^ ^234^ ^]^ Although a range of emulsion‐templated polymers or polymer polyHIPEs have been prepared, not all of them are suitable for fabrication of porous carbon materials. To retain the shape and pore structure of a porous polymer during a carbonization process, carbon‐rich and/or highly crosslinked polymer should be used, for example, polyacrylonitrile (PAN), phenolic resins, etc.^[^ ^234^ ^]^ Alternatively, porous carbon materials may be prepared from building blocks such as CNTs and graphene.^[^ ^235^ ^]^

The detailed preparation and characteristics of the emulsion‐templated porous carbon and graphene‐containing carbon materials are discussed under the following subsections.

#### 2.4.1 Porous Carbon from W/O Emulsions

**Figure 14:**
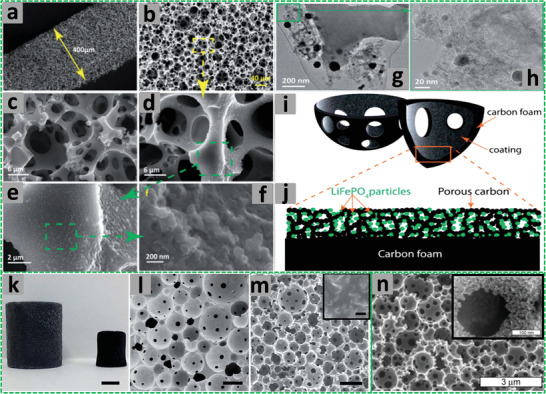
a,b,d–f) SEM images of LiFePO_4_ NPs‐coated carbon foam at different magnifications and c) a bare carbon foam. g,h) TEM images confirm the presence of LiFePO_4_ NPs. i,j) Cartoons show the entrapment of LiFePO_4_ NPs into the pores of carbon to form a LiFePO_4_–carbon composite layer. Adapted with permission.^[^ ^237^ ^]^ Copyright 2014, Royal Society of Chemistry. k) Reduced graphene oxide (rGO)‐stabilized PDVB polyHIPE monolith (left) and the resulting carbon monolith after carbonization (right), scale bar 5 mm; l) SEM image of PDVB monolith, scale bar 100 µm; m) SEM image of the carbon polyHIPE monolith and the inset image showing rGO platelets, scale bar 100 µm and inset scale bar 400 nm. Adapted under a Creative Commons Attribution 3.0 Unported Licence.^[^ ^241^ ^]^ Copyright 2018, The Author(s), published by the Royal Society of Chemistry. n) SEM images showing the HIPE‐templated porous carbon and the mesoporous pore wall (in the inset). Reproduced with permission.^[^ ^233^ ^]^ Copyright 2010, American Chemical Society.

With the use of W/O HIPE templating, preparation of P(St‐*co*‐DVB) or polydivinylbenzene (PDVB) has been mostly reported. However, direct carbonization of PS polyHIPEs usually results in the collapse of monolithic and porous structures. Post‐treatments such as oxidation or further crosslinking of PS polyHIPEs are necessary to produce hierarchically porous carbon monoliths. For example, a polyHIPE was made from St/DVB/VBC and subsequently sulfonated by acetic anhydride and concentrated H_2_SO_4_ in DCE. Carbonization of the sulfonated polymer at 700 °C under N_2_ produced a porous carbon material with macroporous/mesoporous structures and a surface area of 433 m^2^ g^−1^.^[^ ^236^ ^]^ The same group also treated P(St‐*co*‐VBC‐*co*‐DVB) with concentrated H_2_SO_4_ overnight. After washing with water and drying at 100 °C, the dry polyHIPE was carbonized at 900 °C in Ar. A further deposition of LiFePO_4_ NPs into the porous carbon enabled a good performance for 3D micro‐batteries (**Figure** **14**a–j).^[^ ^237^ ^]^

Silverstein and co‐authors compared the direct carbonization of poly(St‐*co*‐VBC‐*co*‐DVB) and the carbonization of hypercrosslinked poly(St‐*co*‐VBC‐*co*‐DVB).^[^ ^230^ ^]^ Higher mass/volume loss, reduced porosity, and fused wall structure were observed for the directly carbonized sample. The hypercrosslinking process led to a porous polymer with a very high surface area of 1473 m^2^ g^−1^ and also contributed to smaller mass loss and limited collapse of macropore structure during carbonization. This enabled the preparation of hierarchically porous carbon materials containing both macropores and micropores showing a BET surface area of 553 m^2^ g^−1^.^[^ ^230^ ^]^ Woodward et al. hypercrosslinked a P(St‐*co*‐DVB) polyHIPE and after carbonization obtained porous carbon materials with surface area of up to 417 m^2^ g^−1^ and an excellent electrical conductivity.^[^ ^93^ ^]^ Although PAN is a widely known carbon precursor, the preparation of PAN polyHIPEs has been highly challenging. Cohen and Silverstein successfully prepared PAN‐based polyHIPEs using a polyglycerol polyricinoleate surfactant, with acrylonitrile (AN)/DVB (AN content varied 82.5–94.8 wt%) as monomer/crosslinker.^[^ ^238^ ^]^ These polyHIPEs could be directly pyrolyzed under N_2_, generating macroporous/mesoporous carbon materials but with low surface areas.^[^ ^238^ ^]^

Woodward et al. prepared PDVB polyHIPEs using polymeric surfactants (Span 80 and Hypermer 2296) and silica NPs as stabilizers.^[^ ^239^ ^]^ The polyHIPE monoliths obtained from polymeric surfactants collapsed after carbonization at 800 °C under N_2_ while a macroporous carbon monolith with a surface area of 505 m^2^ g^−1^ was obtained when silica NPs were also used as stabilizers. This carbon monolith showed an excellent electrical conductivity of 81 S m^−1^. It was believed that silica NPs in the pore wall maintained the macropore structure during carbonization.^[^ ^239^ ^]^ When the silica nanoparticle‐stabilized PDVB was further treated with aqueous potassium hydroxide (KOH) solutions of 10 and 30 wt% for activation, emulsion‐templated porous carbon monoliths with high surface areas of 1123 and 1450 m^2^ g^−1^ were generated.^[^ ^240^ ^]^ In a further study, Woodward et al. employed amphiphilic rGO instead of silica NPs as stabilizers for the preparation of PDVB polyHIPEs. Without any post‐treatment, the carbonized monoliths displayed interconnected macroporous structures, very high surface areas (up to 1820 m^2^ g^−1^), and excellent electrical conductivities (up to 285 S m^−1^) (Figure 14k–m).^[^ ^241^ ^]^ He et al. prepared PDVB polyHIPE with toluene as porogen in the monomer phase with the surface areas in the range of 440–711 m^2^ g^−1^. After KOH treatment (no other oxidation or crosslinking treatment), carbonization at 700 °C under Ar, and acid washing with 3 m HCl, highly interconnected macroporous carbon monoliths with surface areas of up to 2190 m^2^ g^−1^ were obtained.^[^ ^242^ ^]^

#### 2.4.2 Porous Carbon from O/W Emulsions

Compared to W/O emulsions, a larger variety of porous polymers have been prepared from O/W emulsions and pyrolyzed to make hierarchically porous carbon materials. As effective precursors for carbon materials, resorcinol–formaldehyde (RF) resin polyHIPEs have been prepared and employed for the production of hierarchically porous carbon materials. Gross and Nowark mixed formaldehyde, resorcinol, and sodium carbonate in deionized water. Then, SDBS was added as a surfactant followed by the addition of silicone oil. The mixture was blended to get a white‐colored viscoelastic emulsion. After polymerization/aging, washing, and drying, the RF polyHIPE was pyrolized at 800 or 1200 °C for 6 h. The carbon foams exhibited surface areas of 187 and 593 m^2^ g^−1^, respectively. Furthermore, the macro‐ and mesoporosity of these monoliths could be independently controlled without using any hard template, whereas the internal oil phase and the aqueous external phase determined the macropore dimension and mesopore size distribution, respectively (Figure 14n).^[^ ^233^ ^]^

Liu et al. also investigated the formation of RF‐based polyHIPEs using liquid paraffin as the oil phase. Silica colloid sol was added into the aqueous phase that also contained RF precursors. This process led to the formation of silica nanoparticle‐embedded RF resin polyHIPEs. The carbonization process and the subsequent etching of silica by hydrofluoric acid generated porous carbon structures with macropores, open meso‐/macroporous windows, and a large number of micropores. Although this method was good to prepare 3D open‐cell structures with a high surface area of 1086–1501 m^2^ g^−1^, the use of hazardous HF to remove silica undermined its sustainability.^[^ ^243^ ^]^

Zhao et al. prepared phenol‐formaldehyde resin polyHIPEs using a gemini surfactant.^[^ ^244^ ^]^ In comparison, CTAB and Tween 20 were also used as stabilizers for the O/W HIPEs. The resin polyHIPEs were carbonized at 700 °C under N_2_ and further activated with KOH under the same heating condition. Well‐defined interconnected macroporous resin and carbon polyHIPEs with higher surface areas were obtained with the gemini surfactant as compared to those prepared with CTAB and Tween 20 as stabilizers. The activation process increased the surface area of the carbon polyHIPE (prepared from the gemini surfactant) from 738 m^2^ g^−1^ to 1140 m^2^ g^−1^. For the Tween‐20‐stabilized polyHIPE, although the carbon polyHIPE exhibited a much lower surface area (92 m^2^ g^−1^), the activation process increased the surface area dramatically to 1266 m^2^ g^−1^.^[^ ^244^ ^]^

The polycondensation reaction of diols (or compounds with multiple hydroxyl groups) and diamines is an effective route to the preparation of C,N‐rich organic materials. Carbonization of such materials results in the production of N‐rich porous carbon materials. Yang et al. reported the preparation of highly porous N‐rich carbon aerogels with hydrophobic surface and fire‐resistant properties.^[^ ^231^ ^]^ In this regard, an O/W emulsion was prepared by dropwise addition and mixing of toluene into the continuous phase containing melamine and formaldehyde monomers and lignin particles. This lignin particle‐stabilized toluene‐in‐water Pickering emulsion was then cured to produce easily carbonizable melamine‐formaldehyde polymer aerogel. Pyrolyzing this polymer aerogel at 700 °C produced an interconnected macroporous carbon material with a relatively low surface area of 12.5 m^2^ g^−1^.^[^ ^231^ ^]^

Celzard and co‐authors employed renewable chemicals such as tannin and sunflower oil to make emulsion‐templated porous carbon.^[^ ^229^ ^]^ The O/W emulsions were prepared by the addition of sunflower oil into the aqueous phase containing tannin as precursor with hydroxyl groups, hexamine as crosslinker, *p*‐toluenesulfonic acid as catalyst, and a commercial nonionic stabilizer. The emulsions were cured at 85 °C for 20 h. The dry polyHIPE monoliths were carbonized at 900 °C under N_2_. By varying the volume percentage of oil phase, this process produced hierarchical porous carbon materials with tunable porosity, mechanical stability, and surface areas up to 400 m^2^ g^−1^.^[^ ^229^ ^]^

Silverstein and co‐authors utilized a deep eutectic solution and Triton X‐405 as the aqueous phase where the DE polymer was formed by the polycondensation between urea and DHBQ.^[^ ^131^ ^]^ The oil phase of the O/W HIPEs was consisted of cyclohexane, toluene, and terephthaloyl chloride (TCL). The chain extension reaction between TCL and the DE polymer occurred in the HIPEs at RT. The freeze‐dried polyHIPEs were carbonized at 700 °C under N_2_. The chain extension process with TCL enhanced the mechanical and thermal stability of the monoliths. The hierarchical porous carbon monolith displayed bimodal macropore structure, surface area of 812 m^2^ g^−1^, micropore volume of 0.266 cm^3^ g^−1^, and meso/macropore volume of 0.238 cm^3^ g^−1^, and N content of 2.3%.^[^ ^131^ ^]^

**Figure 15:**
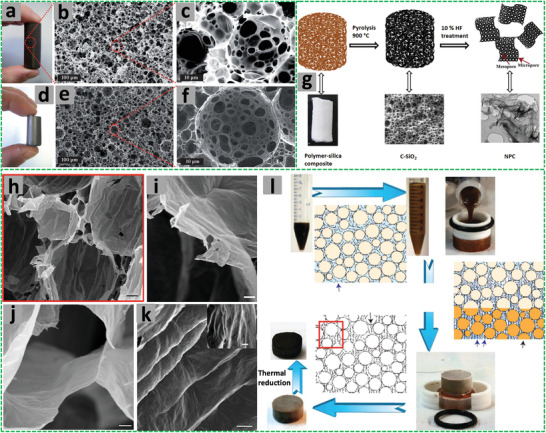
a) Image of HTC‐polyHIPE prepared with an oil percentage of 80%; b,c) corresponding SEM images at low and high magnifications show the macroporous structure; d) the image of pyrolyzed HTC‐polyHIPE at 750 °C; e,f) corresponding SEM images at low and high magnifications show the macroporous structure. Adapted with permission.^[^ ^249^ ^]^ Copyright 2103, Wiley‐VCH. g) Scheme shows the preparation of nanoporous carbon (NPC) sheets from silica‐PAM polyHIPE. Reproduced with permission.^[^ ^251^ ^]^ Copyright 2018, Elsevier. h–k) Porous structure and l) preparation scheme of chemically modified graphene network. h) The emulsion‐templated pore structure, scale bar 10 µm; i) the pore wall, scale bar 2 µm; j) the triple junction between emulsion‐templated pores, scale bar 1 µm; k) the pore wall surface shows the ice‐templated structure, scale bar 2 µm; l) the preparation scheme with the images shows the sample evolution. Reproduced under a Creative Commons Attribution 4.0 International License.^[^ ^259^ ^]^ Copyright 2014, Springer Nature.

Furfuryl alcohol (FA) can be made from renewable biomass and has been commonly used to prepare thermosetting resin poly(furfuryl alcohol) (PFA) via acid‐catalyzed polycondensation reactions.^[^ ^245^, ^246^ ^]^ PFA is a common precursor for preparation of carbon materials with high carbonization yield.^[^ ^247^ ^]^ Szczurek et al. reported the preparation of PFA‐tannin polyHIPEs, employing two renewable precursors of FA and tannin.^[^ ^245^ ^]^ The O/W HIPE was formed by dropwise addition of cyclohexane into the aqueous phase containing tannin, FA, and the catalyst 4‐hydroxybenzenesulfonic acid. However, the authors did not further investigate the preparation of porous carbon by carbonizing the PFA‐tannin polyHIPE.^[^ ^245^ ^]^ Mun et al. prepared emulsion‐templated porous PFA using an O/W emulsion template, wherein the Co(NO_3_)_2_ dissolved in the aqueous phase catalyzed the polymerization of the O/W emulsion at 85 °C. It further facilitated the carbonization process at 850 °C for 2 h under N_2_ and resultantly produced mesoporous partially graphitized carbon with the presence of interconnected macropores and a surface area of 340 m^2^ g^−1^.^[^ ^248^ ^]^ Brun et al. employed a hydrothermal carbonization approach (at 130 and 220 °C) to produce polyHIPEs from furfural and phloroglucinol. Tween 80 was used to stabilize the dodecane‐in‐water emulsions while FeCl_3_ in the aqueous phase was used as the catalyst. The hydrothermally carbonized (HTC) samples were further pyrolyzed under N_2_ at different temperatures (550, 750, and 950 °C). Higher surface area and pore volume (730 m^2^ g^−1^, 0.313 cm^3^ g^−1^) and well‐defined interconnected macroporous structure were observed for the carbon pyrolyzed at 750 °C (**Figure** **15**a–f).^[^ ^249^ ^]^

While crosslinked PAM polyHIPEs are the common porous polymers prepared via O/W HIPE templating, reports on the fabrication of porous carbon from PAM polyHIPEs are very limited. This is due to the collapse of macropore structure and the very low carbonization yield. However, it is possible to prepare porous carbon from porous PAM when an inorganic component is incorporated to enhance the macropore structure. Mazaj et al. dispersed Ti‐beta zeolite NPs into the aqueous phase of an O/W HIPE, which also included AM, MBAM, APS, and TMEDA in the continuous phase. The carbonization of zeolite@PAM polyHIPE was performed in two stages, heating to 450 °C in air to oxidize the PAM matrix and then heating to 900 °C under Ar for carbonization. This produced a zeolite‐embedded carbon material with interconnected four‐length‐scaled ultramicro‐, micro‐, meso‐, and macropores, and a surface area of 362 m^2^ g^−1^.^[^ ^250^ ^]^ Deshmukh et al. introduced TEOS in the oil phase (toluene) of an O/W HIPE and fabricated a PAM‐silica hybrid polyHIPE. Porous silica‐carbon composite was obtained after pyrolyzing at 800–1000 °C under Ar. However, when silica was removed from the composite by etching with 10% HF solution, porous carbon nanosheets were generated (Figure 15g), which were found to be good electrode materials for ultracapacitors.^[^ ^251^ ^]^

#### 2.4.3 Graphene‐Based Emulsion‐Templated Porous Carbon

CNTs and graphene are building blocks with excellent individual properties for fabrication of porous composites and carbon materials.^[^ ^235^ ^]^ However, their use for the emulsion‐templated porous materials has been quite limited. Cohen et al. prepared CNT‐P(St‐*co*‐DVB) polyHIPEs with improved conductivity and mechanical stability. CNTs were dispersed in the aqueous phase of W/O emulsion but were migrated across the O/W interface to form interconnected bundles within the wall.^[^ ^252^ ^]^ There are reports about the preparation of rGO‐polymer polyHIPE composites where GO nanosheets were similarly dispersed in the internal phase of W/O HIPEs.^[^ ^253^, ^254^, ^255^ ^]^ The hydrophobic interaction and *π*–*π* interaction between rGO nanosheets and polymer components have improved conductivity, elasticity, and thermal and mechanical stability of the porous rGO‐polymer composites.^[^ ^253^, ^254^, ^255^ ^]^ However, further thermal treatment of CNT‐ or graphene‐polymer polyHIPE composites has not been well reported. Yuan et al. prepared porous GO‐silica NPs‐P(DVB‐EHA) [poly(divinylbenzene‐2‐ethylhexyl acrylate)] composites via W/O HIPE templating. Both GO and silica NPs were dispersed in the internal aqueous phase. The dry porous composites were carbonized under N_2_ at 600 °C to produce interconnected porous carbon materials, which showed good performance in enriching trifluralin in soil.^[^ ^256^ ^]^

One exciting progress is to use GO nanosheets directly as emulsion stabilizers for the preparation of porous carbon (graphene) materials. Aqueous GO nanosheet dispersion (with ammonia) was slowly added into hot olive oil under intense stirring to form a W/O emulsion. Hollow GO microspheres were formed when the emulsion was heated at 90 °C and then 95 °C.^[^ ^257^ ^]^ Porous graphene foams were prepared from O/W emulsions with trimethylbenzene (TMB) or *n*‐hexadecane as the oil phase. In the preparation, the organic solvent was first added into aqueous HCl solution (2.0 m) to form an O/W emulsion. Precipitates were formed when aqueous GO dispersion was mixed with the pre‐formed emulsion. It was believed that the hydrophobic and *π*‐*π* interactions between TMB and GO nanosheets or the hydrophobic interaction between n‐hexadecane and GO nanosheets were responsible for the formation of precipitates. The precipitates were sintered at 350 °C and further 900 °C under Ar to generate macroporous graphene foams. Hydrophobic silanes (e.g., dimethyldimethoxysilane or surfactants (e.g., CTAB, Pluronic F‐108) could also be added into the oil phase to better control the pore size and improve the surface area.^[^ ^258^ ^]^

Saiz and co‐authors prepared GO suspension by the modified Hummers method and subsequently obtained GO flakes by freeze drying the suspension. The GO flakes were dispersed in water or with PVA:sucrose (1:1) as co‐stabilizer. Toluene was emulsified into aqueous GO dispersion to form an O/W emulsion with 75 v/v% oil phase. The O/W emulsion was directly frozen and freeze‐dried to produce a dry porous GO material, which was then reduced in 10% H_2_/90% Ar atmosphere between 300 and 1000 °C to obtain rGO porous network (Figure 15h–l).^[^ ^259^ ^]^ The freeze‐dried GO materials were also treated in a graphite furnace in the high temperature range of 1000–2400 °C. This method was able to produce complex graphene network with tunable properties for different applications.^[^ ^259^ ^]^ In a further study, graphene aerogel was fabricated from the O/W emulsion with 50 v/v% oil phase and then reduced at 1000 °C in 10% H_2_/90% Ar atmosphere. The resultant aerogel showed very good Joule heating characteristics.^[^ ^260^ ^]^

#### 2.4.4 Summary

Section 2.4 demonstrates the preparation of high surface area porous emulsion‐templated carbon/graphene‐based materials through simple carbonization of polymerized‐HIPE or freeze‐dried polymers. Since the direct carbonization of polyHIPEs is not much effective to tune their pore structures, carbonization of hypercrosslinked polymers or annealing before carbonization or hydrothermal carbonization has been used to serve the purpose. The use of hypercrosslinked polymers also enhanced carbonization yield as well as surface area of the porous carbonized materials. The SiO_2_ and rGO‐stabilized W/O HIPEs‐templated and KOH‐activated polymer‐derived carbon‐based materials exhibited exclusively high surface area. Likewise, the SiO_2_‐embedded RF resin polyHIPEs‐derived porous carbons showed impressive surface areas even higher than those of the simple RF resin polyHIPEs‐derived porous carbon materials. However, the partially graphitized and zeolite‐embedded carbons revealed interconnected hierarchically porous structures. Furthermore, GO‐based emulsion‐templated porous materials showed improved conductivity, thermomechanical stability, and porous network with tunable properties that make them promising candidates for ubiquitous applications. Summary of the preparation methods and properties of emulsion‐templated carbon materials is given in **Table** **4**.

**Table 4:** Summary of preparation methods and porosity properties of emulsion‐templated porous carbon/graphene‐based materials

Carbon material	Carbon precursor and preparation	Porosity/Property^a)^	Ref.
Porous carbon	Carbonized from P(VBC‐DVB), treated with conc. H_2_SO_4_ first, at 700 °C, under N_2_	Pore size: ≈25 µm Surface area: 433 m^2^ g^−1^	[236]
Porous carbon	Carbonization of P(St‐*co*‐VBC‐*co*‐DVB) and hypercrosslinked P(St‐*co*‐VBC‐*co*‐DVB) at 1000 °C under N_2_ Hard to maintain pore structures by direct carbonization Hypercrosslinking improves carbonization yield	Hierarchical porosity (macropore: 10 µm and micropore: 0.77 nm) Surface area: 553 m^2^ g^−1^ Much lower surface area than that of hypercrosslinked polymers	[230]
Porous carbon	Carbonization of hypercrosslinked P(St‐*co*‐DVB) at 800 °C under N_2_ Contents of DVB varied	Surface areas: 223–417 m^2^ g^−1^ Higher surface area with higher content of DVB Conductivity: 288–434 S m^−1^ on average	[93]
Porous carbon	Preparation of poly(acrylonitrile‐divinylbenzene) polyHIPEs Annealed at 250 °C under O_2_ and then carbonized at 960 °C under N_2_	Density: 0.08–0.25 g cm^−3^ N content: 3.8 at% by XPS Surface area: 26.5 m^2^ g^−1^	[238]
Porous carbon	Silica NPs‐stabilized W/O HIPEs Carbonized from silica‐PDVB Silica NPs not removed	Surface area: 505 m^2^ g^−1^ Conductivity: 81 S m^−1^	[239]
Porous carbon	Silica NPs‐stabilized W/O HIPEs Carbonized from silica‐PDVB or KOH‐activated silica PDVB Silica NPs not removed	Surface area: 521 m^2^ g^−1^ for carbonized sample; Surface area: 1456 m^2^ g^−1^ for activated carbon	[240]
Graphene‐porous carbon	rGO stabilized W/O HIPE Carbonized from rGO‐PDVB	Surface area: 1820 m^2^ g^−1^ Conductivity: 285 S m^−1^	[241]
Porous carbon	From PDVB with toluene as porogen KOH activated Carbonized at 700 °C, under Ar	Surface area: 2189 m^2^ g^−1^ Micropore volume: 0.96 cm^3^ g^−1^	[242]
Porous carbon	Carbonization of RF resin polyHIPEs at 800 or 1200 °C, under N_2_ Higher pore volume and surface area from carbonization at 1200 °C	Pore volume: up to 5.26 cm^3^ g^−1^ and surface area up to 593 m^2^ g^−1^ Density: 0.166–0.263 g cm^−3^ Conductivity: up to 0.34 S m^−1^	[233]
Porous carbon	Carbonization of silica‐embedded RF resin polyHIPEs at 850 °C under N_2_ Silica removed by HF etching Impregnated with KOH and activated at 850 °C	Surface area: up to 1501 m^2^ g^−1^ Micropore volume: 0.66 cm^3^ g^−1^	[243]
Porous carbon	Carbonization of polyRF, prepared from O/W HIPEs stabilized by a gemini surfactant, at 700 °C, under N_2_ Further activated with KOH Compared with other surfactants CTAB and Tween 20	Surface area: 738 m^2^ g^−1^ and 1140 m^2^ g^−1^ (after activation) Micropore volume: 16.1 cm^3^ g^−1^ and 23.5 cm^3^ g^−1^ (after activation)	[244]
Porous N‐rich carbon	Carbonization of PMF, prepared from lignin particles‐stabilized O/W emulsions, at 400–700 °C under N_2_	Porosity: 87.7%, surface area: 12.5 m^2^ g^−1^, pore size: 3.6 µm, 12.8% N (carbonized at 700 °C) Higher density and more hydrophobic at higher carbonization temperatures	[231]
Porous carbon	O/W HIPE templated polymers made from tannin and hexamine Carbonized at 900 °C, under N_2_	Bulk density: 0.13–0.28 g cm^−3^ Porosity: up to 93% Surface area: up to 400 m^2^ g^−1^ Intrusion pore volume: up to 7.5 cm^3^ g^−1^	[229]
Porous carbon	Carbonization of poly(DHBQ‐urea) polyHIPE at 700 °C, under N_2_ Chain extending reaction improve the carbonization yield	Macropores: 12 and 46 µm Surface area: 812 m^2^ g^−1^ Density: 0.059 g cm^−3^	[131]
Partially graphitized carbon	PFA prepared from O/W emulsions with Co(NO_3_)_2_ in aqueous phase Carbonization at 850 °C, under N_2_ Co^2+^catalyzed polymerization of FA and promoted graphitization	Hierarchically porosity with interconnected macro‐ and mesopores Surface area: 340 m^2^ g^−1^	[248]
Porous carbon	Hydrothermal carbonization (at 130 or 220 °C) of O/W emulsions with furfural and phloroglucinol as precursors and dodecane as oil phase Further pyrolyzed at 750 °C (and 550 and 950 °C) under N_2_	Surface area: 730 m^2^ g^−1^; total pore volume: 18 cm^3^ g^−1^; conductivity: up to 300 S m^−1^; micro‐/mesopore volume: 0.313 cm^3^ g^−1^	[249]
Zeolite‐embedded carbon	zeolite@PAM polyHIPE prepared Thermally treated at 450 °C in air and then carbonized at 900 °C under Ar	3D‐interconnected hierarchical ultramicro‐, micro‐, meso‐, and macro‐pore system Surface area: 362 m^2^ g^−1^	[250]
Porous carbon nanosheets	Carbonization of silica‐PAM at 800–1000 °C under Ar Etching silica with HF to produce porous nanosheets	Surface areas: 400–1150 m^2^ g^−1^ Optimal carbonization temperature at 900 °C for generating micropores and mesopores	[251]
GO and silica‐doped carbon	Porous GO‐silica‐P(DVB‐EHA) prepared first; Carbonization at 600 °C, under N2	Interconnected macropores: about 30 µm	[256]
Hollow GO microspheres	W/O emulsions formed by emulsifying aqueous ammonia with GO nanosheets into olive oil Microspheres formed when heating emulsions to 90–95 °C.	Hollow structure confirmed by imaging Wall thickness: ≈1 µm	[257]
Porous graphene foam	O/W emulsions with TMB or n‐hexadecane as the oil phase Precipitates formed when mixing the emulsions and aqueous GO dispersion Hydrophobic silanes, CTAB, or F‐108 added into the oil phase for pore size control and enhancing surface area The precipitates sintered at 350 and then 900 °C, under Ar	3D porous structure surface area: 451–611 m^2^ g^−1^ high conductivity at 35 kS m^−1^	[258]
Porous graphene network	First prepare GO‐stabilized toluene‐in‐water emulsion with PVA/sucrose as stabilizer; Directional freezing and freeze‐drying Further thermal treatments (300–1000 °C in 10% H_2_/90% Ar atmosphere or 1000–2400 °C in a graphite furnace under high vacuum)	Very low density: 1 mg cm^−3^ Properties tunable based on preparation conditions.	[259]

^a)^ Surface area values are for BET surface areas and pore volumes are measured by Hg intrusion porosimetry. Bulk density data are always quoted here for “density”.

## 3 Environmental Applications of Emulsion‐Templated Porous Materials

Environmental catastrophes, textiles, steel and mining industries' disposal, energy plant effluents, sewage discharge, agricultural runoff, and oil spilling has degraded the quality of water and air and in turn adversely affected public health because of their inherent toxicity to the central nervous system, brain tissue, reproductive system, and children's intellectual development. In this context, the development of innovative materials for environmental remediation and sensing is indispensable. Many promising new porous material‐based technologies are pressingly acclaimed to offer consolidated solutions for water and air protection and monitoring.^[^ ^102^, ^103^, ^261^ ^]^

Among all sorts of pollution, water contamination has been considered to be the foremost threat. Water treatment includes multi‐stage primary, secondary, and tertiary treatment processes. Primary treatment may be of physical and/or chemical nature that involves preliminary water purification processes (i.e., screening and microfiltration, sedimentation, centrifugation, chemical precipitation, coagulation, gravity, and flocculation) before applying further refined treatments. Secondary treatment is broadly categorized into anaerobic and aerobic treatment types, which depends on naturally occurring microorganisms capable of transforming water pollutants into relatively safer and simpler substances. However, tertiary treatment processes involve chemical oxidation, crystallization, electrochemical precipitation, distillation, membrane processing, ion exchange resins, adsorption, and photocatalysis.^[^ ^261^ ^]^

Porous materials have gained enormous consideration in adsorption, separation, catalysis, purification, etc., but there is no one‐size‐fits‐all solution; it is thus challenging to select a right kind of porous material for the specific application at community scale. By way of example, porous polymeric networks are usually amorphous but robust owing to strong covalent bonds, whereas MOF materials are typically crystalline but, in some cases, metal‐ligand bond is unstable to water. The amorphous materials with hierarchical pores can be advantageous for heterogeneous catalysis, whereas crystallinity is beneficial for molecular sieving, wherein uniform pores are desirable. Likewise, 3D frameworks and networks are, by definition, insoluble, whereas porous molecular materials and polymers of intrinsic microporosity possess no formal intermolecular bonds and may thus be processed as the molecular solutions to prepare gas separation membranes.^[^ ^14^, ^262^ ^]^

Many state‐of‐the‐art porous materials such as zeolites, MOFs, covalent‐organic frameworks, and porous polymers have been used for environmental applications though; practical deployment of most of all such materials to develop practically viable decentralized technologies is hindered by their complicated processability, leaching, and subsequent recovery. In this perspective, it is necessary to optimize multiple functions such as stability, sorption (adsorption/desorption) kinetics, processability, stability (i.e., hydrolytic, mechanical, thermal, chemical, or photolytic stability) together with pore structure, mass transport behavior, and surface area. In this connection, the hierarchically porous materials having high pore volume ratios, high surface areas, high accessibilities, and ready mass transport properties are considered to be superior to other simple porous materials for adsorption, separation, catalysis, and sensing.^[^ ^4^, ^14^ ^]^

In the same vein, the emulsion‐templated porous materials have been frontrunners to offer practically convincing water treatment solution owing to their hierarchical interconnected porous tunable structures that offer easy permeability, homogeneous flow‐through, high diffusion rates, convective mass transfer, and direct accessibility to interact with atoms/ions/molecules throughout the exterior and interior of the bulk. Above all, the robustness and macrosize of the emulsion‐templated porous materials favor easier handling and subsequent recovery that broaden their horizons for the development of point‐of‐use water purification systems.^[^ ^98^, ^101^, ^102^, ^103^ ^]^

Many review articles demonstrated general applications of the emulsion‐templated porous materials especially organic polymers. The present review for the first time comprehensively discusses recent research progress of emulsion‐templated porous polymers, organic‐inorganic hybrids, inorganic structures, as well as carbonaceous/graphene‐based materials in environmental applications (e.g., adsorption, separation, disinfection, catalysis/degradation, capture, and sensing of the inorganic, organic, and biological contaminants in water and air).

The emulsion‐templated porous materials have shown great potential to remove as well as detect metal ions, dyes, surfactants, pigments, additives, drugs, pesticides, microbial pathogens, etc., in wastewater. They have also been employed for gas capture and environmental sensing applications. The remediation performance (removal efficiency and capacity, etc.), sensing capability (sensitivity, selectivity, etc.), and practical value (scalability, leachability, processability, recyclability, durability, etc.) of different types of adsorbents and photocatalysts (membranes, microspheres, beads, monoliths, etc.) for a wide variety of pollutants under certain experimental/operational conditions (dose of emulsion‐templated porous materials, initial pollutant concentration, pH of media, water flux, pressure, etc.) are also summarized in **Table** **5**. The advantages of different emulsion‐templated porous materials for practical water treatment are mentioned in each case.

**Table 5:** Environmental applications of the emulsion‐templated porous materials

Material	Target	Performance	Advantages	Ref.
CS‐ PAA hydrogel	Removal of: Cu^2+^ Pb^2+^	Adsorption capacity: 302 mg g^−1^ 613 mg g^−1^	Easily regenerated for five cycles	[212]
CMC‐MMT‐PAM	Removal of: Pb^2+^ removal Cd^2+^ removal	Adsorption capacity: 456 mg g^−1^ 278 mg g^−1^	Five adsorption–desorption cycles without any significant loss of uptake capacities	[272]
Fe_3_O_4_ NPs‐P(St‐co‐DVB)	Removal of: Pb^2+^ Cd^2+^	Adsorption capacity: 257 m^2^ g^−1^ 129 m^2^ g^−1^	Easily separated and recycled by a magnet	[197]
Magnetic HPC‐PAA beads	Removal of: Cd^2+^ Cu^2+^	Adsorption capacity: 300 mg g^−1^ 243 mg g^−1^	Easy separation with a magnet, similar capacities for five cycles	[194]
PAA−Fe_3_O_4_ NC beads	Removal of: Crystal violet (CV) Pb^2+^ removal	Adsorption capacity: 80 mg g^−1^ 291 mg g^−1^	Easily handled No leaching of iron	[103]
Organo‐silica foam	Removal of: Sunset yellow MB Cu^2+^	Adsorption capacity: 1.21 g g^−1^ 280 mg g^−1^ 226 mg g^−1^	Materials containing amino, epoxy, and carboxyl groups	[172]
Amino‐organosilica monolith	Cr^4+^ removal	Removing efficiency: 92.8%	Working capacity: 4.24 kg g^−1^	[275]
FeOOH NP hydrogels	As^5+^	Removal efficiency: 50%	Easily recovered	[198]
Al_2_O_3_‐PAA‐PEI	Cr(VI) CR	141 mg g^−1^ 37 mg g^−1^	No chance of secondary contamination	[201]
Ag NPs‐PVI beads	Adsorption of: As^3+^ Eriochrome black T	Adsorption capacity: 333.36 mg g^−1^ 81.14 mg g^−1^	Multifunctional for removing inorganic, organic and biological contaminants Easily handled	[102]
	Inactivation of *S*. *aureus*	Bactericidal efficiency: ≈96%		
	Inactivation of *E*. *coli*	Bactericidal efficiency: ≈100%		
Amine‐modified PEGMA	Removal of: Ag^+^ Cu^2+^ Cr^3+^	Adsorption capacity: 9.05 mmol g^−1^ 4.31 mmol g^−1^ 2.92 mmol g^−1^		[156]
Polyelectrolyte‐based polyHIPEs	Ag^+^ ion exchange	Exchange capacity: 3.53 mmol g^−1^		[146]
GMA‐based microspheres	Adsorption of Li^+^	Adsorption capacity: 38.13 mg g^−1^	Selective removal Recycled five times	[277]
Ag‐modified PS sulfonate	Removal of Li^+^	Adsorption capacity: 59.85 mg g^−1^ at 15 °C 35.06 mg g^−1^ at 25 °C 27.09 mg g^−1^ at 35 °C	Adsorption efficiency: 80.71% after being recycled seven times	[204]
Amidoxime‐modified hollow MF resin microspheres	UO_2_^2+^ removal	Adsorption capacity: 553.3 mg g^−1^	Higher selectivity in the presence of other ions	[165]
P4VP grafted P(St‐*co*‐DVB)	Pu separation		Ion exchange column with convective mass transfer property	[115]
CMC‐PAM/MMT	Removal of: Rb^+^ Cs^+^	Adsorption capacity: 178 mg g^−1^ 266 mg g^−1^		[219]
Yeast‐PAA	Removal of: Rb^+^ Cs^+^ Sr^2+^	Adsorption capacity: 180 mg g^−1^ 230 mg g^−1^ 167 mg g^−1^	Materials recycled five times with good performance	[155]
CS‐*g*‐PAM	MB removal	Adsorption capacity: 454 mg g^−1^		[139]
UiO‐66‐PAM	MB removal	Adsorption capacity: 50 mg g^−1^		[142]
PDMAEMA and HEMA polymers	Adsorption of: MB methyl orange (MO)	Adsorption capacity: 6.5 mg g^−1^ 1.6 mg g^−1^		[157]
Polyampholytes	Removal of: MB Erythrosine	Adsorption capacity: 88 mg g^−1^ 57 mg g^−1^		[147]
Porous PAM microspheres	Removal of: MB methyl violet (MV)	Adsorption capacity: 669 mg g^−1^ 750 mg g^−1^	Prepared by O/W/O emulsion templating	[169]
TiO_2_/P(AM‐co‐AMPS) monoliths	Removal of: MB TC	Adsorption capacity: 1.66 g g^−1^ 1.13 g g^−1^		[134]
Molecularly imprinted polymers hollow microspheres	Removal of *λ*‐cyhalothrin	Adsorption capacity: 24.79 mg g^−1^	Material can be recycled seven times with 8.11% loss of affinity	[167]
P(St‐*co*‐NPA) beads	Removal of atrazine	Removal efficiency: 98%		[117]
Graphene and silicon‐doped porous carbon	Removal of trifluralin	Removal efficiency: up to 100%	Used as a column for solid phase extraction to detect trifluralin in soil samples	[256]
Fe_3_O_4_@HKUST‐1‐embedded P(EHA‐DVB‐MMA) [poly(2‐ethylhexyl acrylate‐divinylbenzene‐methyl methacrylate)]	Sorptive extraction of TC	Limit of detection: 1.9–4.6 and 5.5–13.9 ng mL^−1^ for milk and egg samples of chicken Limit of quantification: 1.8–3.7 and 5.3–13.0 ng g^−1^ for muscle and kidney samples of chicken		[215]
Modified Fe_3_O_4_ NPs‐CS‐PAMPS porous adsorbents	Removal of: TC Chlorotetracycline (CTC)	Adsorption capacity: 806.60 mg g^−1^ 876.60 mg g^−1^	Material recycled five times	[193]
rGO‐polyHIPE hybrids	Adsorption of polyaromatic hydrocarbons (PAHs)	Adsorption capacity: 47.5 mg g^−1^	Reused for ten consecutive cycles	[254]
poly(4‐vinylbenzyl chloride) (PVBC) monolithic columns	Removal of benzoyl chloride	Removal efficiency: 98%		[116]
P(St‐EGDMA)	Adsorption of: Toluene Benzene	Adsorption capacity: 0.777 g g^−1^ 0.907 g g^−1^	Material can be reused ten times	[120]
P(St‐*co*‐VBC‐*co*‐DVB) monoliths	Absorption of chemical warfare agents	Mass increase 40–55 times that of dry polymer		[113]
P(St‐*co*‐DVB) aerogels	Adsorption of organic liquids and oil	Adsorption capacities: 13–29 g g^−1^	Material stable and reusable	[110]
P(St‐*co*‐DVB)	Adsorption of: *n*‐hexane Mineral ether Kerosene Benzene THF DCM Salad Gasoline Machine oil	Adsorption capacity: 4.45 g g^−1^ 5.02 g g^−1^ 6.19 g g^−1^ 18.71 g g^−1^ 15.23 g g^−1^ 20.21 g g^−1^ 2.75 g g^−1^ 16.49 g g^−1^ 2.51 g g^−1^	Materials recycled ten times	[109]
Silica‐P(*t*BMA‐DVB) composites	Adsorption of: *n*‐hexane Benzene DCM THF Ethanol Methanol Acetone Kerosene oil Used‐transformer oil	Adsorption capacity: 3.86 g g^−1^ 15.37 g g^−1^ 17.33 g g^−1^ 13.42 g g^−1^ 5.61 g g^−1^ 4.52 g g^−1^ 3.43 g g^−1^ 8.17 g g^−1^ 4.98 g g^−1^	Materials recycled at least 13 times	[181]
PPI‐SPS‐*b*‐PE‐*r*‐Bt‐*b*‐PS xerogels	Adsorption of: Hexane Toluene Xylene DCE Chloroform Vegetable oil Gasoline Diesel Engine oil Crude oil	Adsorption capacity: 19.8 g g^−1^ 22.3 g g^−1^ 24.9 g g^−1^ 22.6 g g^−1^ 32.2 g g^−1^ 24.2 g g^−1^ 21.2 g g^−1^ 23.8 g g^−1^ 31.5 g g^−1^ 15.1 g g^−1^	Materials recycled at least 40 times	[126]
SPS‐*b*‐PE‐*r*‐Bt‐*b*‐PS and PAMAM dendrimers‐based sponges	Removal of: *n*‐hexane Chloroform Crude oil	Adsorption capacity: 25.4 g g^−1^ 25.8 g g^−1^ 14.4 g g^−1^	Material recycled 30 times with ≈15% loss of performance Oil/water separation	[125]
Fluorinated P(PEM‐St‐DVB) material	Remove of DCM	Removal efficiency: 95%	Materials reused for ten cycles	[122]
PDMS‐infused PS‐based slippery membrane systems	Liquid (oil and water) and solid‐ contaminant repelling		Materials showing self‐repairing and regeneration properties	[171]
PU monoliths	Oil spill reclamation	Recovery rate: ≈85%	Materials recycled 20 times	[128]
Cellulose‐based PU	Adsorption of: DMSO Non‐polar solvent	Adsorption capacity: 22 cm^3^ g^−1^ 9.5 cm^3^ g^−1^	Void filling, no expansion	[129]
PU with reactive block copolymer	Adsorption of: Chloroform DCE	Adsorption capacity: 36 g g^−1^ 27.3 g g^−1^	2 min half equilibrium time	[130]
Silica−polyHIPE composite networks	Sorption/desorption of crude oil	Sorption/desorption capacity: ≈16 g g^−1^	Material reused for 25 cycles	[190]
Fe_3_O_4_/PS composites	Diesel/water separation	Adsorption capacity: 7.9 g g^−1^	Materials recycled ten times	[196]
	Edible oil/water separation	Adsorption capacity: 8.3 g g^−1^		
	Lubricating oil/water separation	Adsorption capacity: 16.4 g g^−1^		
P(St‐*co*‐DEAEMA) membranes	Oil/water separation	Chloroform/water, hexane/water	CO_2_‐switchable separation	[119]
sPS‐based nanofibrous monoliths	Adsorption of: Organic solvent Edible oil Fuel oil	Adsorption capacity: 81.3 g g^−1^ 44.4 g g^−1^ 41.9 g g^−1^	Highly reusable, loss of 10% uptake capacity after 20 cycles.	[112]
Graphene foams	Removal of oil (toluene, hexadecane, and olive oil)			[258]
rGO‐based cellular network	Adsorption of diesel, gasoline, motor oil, petroleum, and toluene	Adsorption capacities: 100–300 g g^−1^ using the material with a density of 4.3 mg cm^−3^; over 600 g g^−1^ with a lower density material (1.5 mg cm^−3^)	Recycled by compression for at least six cycles with the capacity maintained at >95%	[259]
Ag NPs‐PVSA beads	Adsorption of: Hg^2+^ Rhodamine B	Adsorption capacit: 190.58 mg g^−1^ 53.02 mg g^−1^	Multifunctional in removing inorganic, organic, and biological contaminants Easily handled	[98]
	Inactivation of *S*. *aureus*	Bactericidal efficiency: 98.39%		
	Inactivation of *E*. *coli*	Bactericidal efficiency: ≈100%		
Ag‐m‐MOP‐PAM composites	Catalytic reduction of 4‐nitrophenol	Rate of reaction: 0.037–0.197 min^−1^		[205]
Fe_3_O_4_‐PAM beads	Decomposition of MO	Decomposition efficiency: 99.6%	Material reused six times	[195]
TiO_2_ beads	Photodegradation of MB	Degradation efficiency: 98.53%; rate constant = 0.05 min^−1^	Photodegrading the organic and photodisinfection of biological contaminants	[101]
	Photodisinfection of *E*. *coli*	Bactericidal efficiency: 100%		
	Photodisinfection of *S*. *aureus*	Bactericidal efficiency: 100%		
PAE‐based polyHIPEs	Degradation of bisphenol A (BPA)	Degradation efficiency: 98%	Organic photocatalyst Visible light‐active	[147]
P(St‐*co*‐DVB) impregnated with PEI	CO_2_ capture from different sources	Adsorption capacity: 5.6 mmol g^−1^ (pure CO_2_); 4.5 mmol g^−1^ (10% CO_2_/N_2_); 6.4 mmol g^−1^ (under moisture)	High CO_2_/N_2_ selectivity, fast kinetics, stability	[108]
P(St‐*co*‐DVB)/nano‐TiO_2_/PEI	CO_2_ capture	Capture capacity: 5.25 mmol g^−1^	Rapid adsorption/desorption within 10 min; reused 50 times with a capacity loss of less than 10%	[199]
PGD‐HKUST‐1‐PEI	CO_2_ capture from different sources	Adsorption capacity: 4.3 mmol g^−1^ (pure CO_2_); 3.0 mmol g^−1^ (simulated flue gas); 1.8 mmol g^−1^ (air)	2.8 mmol (CO_2_ from simulated flue gas) per gram after 20 cycles	[217]
CNT‐PEI foam	CO_2_ capture	Capture capacity: 2.555 wt%	Highly recyclable	[288]
HIPE templated p(NMe_3_^+^–MS OH^−^) material	CO_2_ capture	Overall sorption rate: 2.5 × 10^−2^ mmol; Swing size: 4.9 × 10^−1^ mmol g^−1^	Reversible CO_2_ capture by humidity swing	[186]
P(VBC‐DVB) modified with quaternary ammonium hydroxide groups	CO_2_ adsorption	Adsorption rate: up to 1.1 × 10^−1^ mmol min^−1^ g^−1^; desorption rate = up to 3.5 × 10^−2^ mmol min^−1^ g^−1^; overall rate = up to 2.5 × 10^−2^ mmol min^−1^ g^−1^ Swing size: up to 7.2 × 10^−1^ mmol g^−1^		[291]
Zeolite‐embedded PAM‐derived carbon foams	CO_2_ capture	Capture capacity: 5 mmol g^−1^ Regenerated by electric swing adsorption	CO_2_/N_2_ selectivity of up to 80 70% performance retention after 30 cycles under humid conditions	[250]
Silica‐P(St‐*co*‐DVB)	Removal of particulate matters (PM_2.5_)	Removal efficiency: 93% Saturated adsorption capacity: ≈520 mg g^−1^	Easily separated, recycled	[187]
P(St‐*co*‐MMA) monolith filter	Removal of particulate matters (PM_2.5_) and CO_2_	Removal efficiency: 73% for PM2.5 and 77.2% for CO_2_	Materials showing excellent dust loading capacity and good resistance	[118]
Zwitterionic hydrogel polyHIPEs	Environmental sensitivity	Anti‐polyelectrolyte effect		[145]
Modified P(St‐DVB‐EHA) membranes	Sensing K^+^ ions	Improved detection limits and selectivity [compared to poly(vinyl chloride) (PVC) membranes]	Nernstian response to K^+^ ions	[121]
Porous Co_3_O_4_	NH_3_ gas sensing	Sensitivity 146% and response time 2 s for a concentration of 100 ppm	Limit of detection: 0.5 ppm	[293]
rGO/PolyHIPE foam	Pressure sensing	High sensitivity (over 0.6 Pa to 200 kPa pressure range) Response time: less than 15.4 ms Cyclic stability: at least 10 000 cycles		[253]
rGO/polyHIPE foam	Pressure sensing	Gauge factor: 1.5 within 15% strain Pressure response range: up to > 200 kPa Pressure sensitivity: 0.83 kPa^−1^ for pressure <20 kPa		[255]

### 3.1 Adsorption, Separation, and Disinfection

Generally, pollutants exist as complex mixtures in contaminated water. The key aspect to carry out environmental remediation is the type of pollutant. In broader sense, the pollutants may be classified into inorganic (heavy metals, halides, radioactive waste, etc.), organic (dyes, pharmaceuticals, agrochemicals, PAHs, etc.), and biological (bacteria, viruses, fungi, etc.) pollutants.

The natural clay minerals generally containing MMT, laponite, sepiolite, hectorite, vermiculite, rectorite, saponite, zeolite, kaolinite, and chlorite have been extensively employed as adsorbents to eliminate environmental pollutants due to their wide availability and cost‐effectiveness. These are also considered superior to many other commercially available adsorbents such as activated carbon owing to their abundance, low cost, and exceptional adsorption performance, but low surface area, crystal structure and negative charge, absence of standard protocols for their recycling in aqueous systems, and narrow potential for the remediation of micro‐pollutants undermine their practical usage. The agriculture‐based bio‐adsorbents offer economic benefits in water treatment though; they boost total organic carbon, biological oxygen demand, and chemical oxygen demand in water thereby limiting practical worth. The polymeric resins have been used as good alternatives to circumvent such concerns, but their pH dependency, poor water wettability, and particle size sensitivity are yet to be addressed for their practical utility. Likewise, the development of activated carbon, CNTs, silica gel, AO, and metal/metal‐oxide nano‐composite/materials presents promising solution to overcome limitations associated with the polymeric materials and also improve treatment efficiency, but the high cost, poor recyclability, leaching problem, and ecoconcerns still undermine their field applications for environmental remediation.^[^ ^263^, ^264^ ^]^

In this regard, the emulsion‐templated porous materials offer leapfrogging prospects for adsorption, separation, and disinfection applications on account of their hierarchically porous structure, high surface area, good sorption kinetics, easier processability, and intriguing mechanical and thermal properties. Owing to higher diffusion rates inside their well‐defined interconnected meso‐ and macro‐pores and the associated improved activity to reactants, the emulsion‐templated porous materials are highly advantageous for applications especially involving large molecules. The smaller pores bestow a high specific surface area, the large pores permit efficient and fast mass transport, and the active functional groups contribute to the higher efficiency and affordability, thereby mutually favoring stable adsorption processes.^[^ ^94^, ^103^ ^]^

#### 3.1.1 Adsorption of Inorganic Pollutants

Several methods have been proposed for the wastewater treatment; adsorption has gained enormous research consideration on account of its environmental friendliness, easier processing, high efficiency, low cost, easy scalability, and versatility for various water streams containing different types of highly toxic ions. In adsorption, the pollutant adsorbs onto the surface of the sorbent via physisorption (non‐covalent such as hydrogen bonding and van der Waals forces) and/or chemisorption (covalent). The adsorption capacity of a material mainly depends on the nature of the adsorbent (particle size, surface chemistry, pore size/structure, dosage, etc.), the adsorbate (p*K*_a_, functionality, polarity, molecular weight, size, initial concentration, etc.), and the experimental conditions (pH, contact time, temperature, ionic strength, the presence of other pollutants, etc.).^[^ ^102^, ^263^, ^265^ ^]^

The surface, ground, and tap water have mainly been contaminated with heavy elements, trace metals, noble metals, lanthanides, actinides, and non‐metallic elements. For humans, the toxicity level of some selected metals follows the order Hg > Cd > Cu > Zn > Ni > Pb > Cr > Al > Co. The emulsion‐templated porous adsorbents such as simple and functionalized polymer and hybrid/composite microspheres, macrobeads, and monoliths have effectively been used to remove inorganic pollutants such as Cd, Pb, Cu, As, Ag, Li, U, Pu, Rb, and Cs ions from water.^[^ ^266^ ^]^

These conventional pollutants come from plastics, glass manufacturing, microelectronics, batteries manufacturing, electroplating, painting, pigments, dyeing, coatings, mining, fertilizer, pharmaceutical industries, production of nuclear powered electricity and nuclear weapons, nuclear power plant accidents, natural geological processes, and anthropogenic activities on account of rapidly increasing industrialization and urbanization. These inorganic pollutants lead to severe health problems including kidney infection, renal abnormalities, renal damage, lung infection, heart attacks, liver infection, mental retardation, edema to the immune system, asthma, anemia, numerous types of cancer, allergic reactions, hypertension, DNA damaging, and reduction in fertility in animals and human.^[^ ^103^, ^267^, ^268^, ^269^ ^]^

##### Pb^2+^/Cd^2+^/Cu^2+^

Pb, Cd, and Cu are typically considered to be the conventional water contaminants. Pb is one of the most toxic heavy metals and permissible to be 0.01 and 0.015 mg L^−1^ in drinking water according to the World Health Organization (WHO) and US Environmental Protection Agency (USEPA). Its exposure may occur by ingesting contaminated food and water or via inhalation of contaminated aerosols and dust particles and may cause Pb poisoning in humans, which in turn damages the brain, heart, liver, kidneys, nervous system, and skeleton. Likewise, Cd heavy metal is chronically toxic to the respiratory, cardiovascular, renal, and skeletal systems and may cause lungs, prostate, kidneys, and stomach cancers in children. The people's exposure to Cd may occur through smoking cigarettes, eating contaminated food, and working in Cd‐contaminated work places as well as in primary metal industries. The regulatory limit of Cd in drinking water set by WHO and USEPA is 0.003 and 0.005 mg L^−1^, respectively. Cu is a vital trace element that plays an essential role in enzyme synthesis, tissues, and bone development in human bodies, though Cu(II) ions may also be poisonous and carcinogenic and cause gastrointestinal distress after short‐term exposure and liver and kidney damage as well Wilson's Disease after long‐term exposure. It may come into the environment through erosion of natural deposits and corrosion of household plumbing systems. The permissible concentration of Cu in drinking water recommended by WHO and USEPA is 2 and 1.3 mg L^−1^, respectively.^[^ ^266^, ^268^, ^270^, ^271^ ^]^

**Figure 16:**
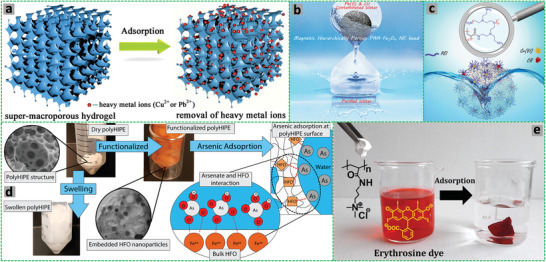
Graphics show removal of a) Cu^2+^ and Pb^2+^ ions by supermacroporous CS‐PAA monolithic hydrogel. Adapted with permission.^[^ ^94^ ^]^ Copyright 2016, Elsevier. b) Pb(II) and CV by magnetic hierarchically porous PAA‐Fe_3_O_4_ NC bead. Reproduced with permission.^[^ ^103^ ^]^ Copyright 2019, American Chemical Society. c) Cr(VI) and CR by PAA‐AO‐PEI bead. Reproduced with permission.^[^ ^201^ ^]^ Copyright 2021, American Chemical Society. d) Schematic illustrates the plausible mechanism of arsenate adsorption by hydrated ferric oxides NPs‐embedded porous AMPS‐based polyHIPE monoliths. Reproduced with permission.^[^ ^198^ ^]^ Copyright 2019, American Chemical Society. e) Image reveals adsorption of erythrosine dye by (3‐acrylamidopropyl)‐trimethylammonium chloride‐based cationic polyelectrolyte monolith. Reproduced with permission.^[^ ^146^ ^]^ Copyright 2018, American Chemical Society.

The Cu^2+^ and Pb^2+^ ions were effectively removed with rapid adsorption kinetics and good adsorption capacities using macroporous CS‐PAA hydrogels owing to their 3D interconnected supermacroporous structure. This material showed adsorption capacities of 302 and 613 mg g^−1^ in the optimized pH range of 4–6 for Cu^2+^ and Pb^+2^, respectively (**Figure** **16**a). These materials were found effective to be reused up to five cycles with slight decrease in adsorption performance.^[^ ^94^ ^]^ The CMC‐MMT‐PAM‐based macroporous monoliths prepared via Pickering MIPE (50 v/v% internal phase) templating were used to remove heavy metal ions from water. Such materials exhibited adsorption capacities of 456 and 278 mg g^−1^ for Pb^2+^ and Cd^2+^, respectively. The macroporous structures were retained after five adsorption–desorption cycles without any significant loss of uptake capacities.^[^ ^272^ ^]^

Amine‐functionalized Fe_3_O_4_ NPs‐P(St‐*co*‐DVB) composites were used to efficiently adsorb Pb^2+^ and Cd^2+^ ions from water with removal capacities of 257 and 129 mg g^−1^ achieved within 15 min, respectively. The adsorption performance was influenced by solution pH and ionic strength due to the presence of amine groups. The authors related the process of adsorption with the ion exchange and cation‐*π* interactions between aromatic rings on the surface of MPPs and heavy metals. The magnetic adsorbents could be easily separated using a magnet and subsequently recycled.^[^ ^197^ ^]^ Magnetic hydroxypropyl cellulose (HPC)‐PAA beads were reported for their excellent potential in removing heavy metal ions. Using these materials, the adsorption process of Cd^+2^ and Cu^+2^ ions reached equilibrium within 20 min with capacities of 300 and 243 mg g^−1^, respectively. These beads could be re‐used over five consecutive cycles of adsorption–desorption with similar adsorption capacities.^[^ ^194^ ^]^ Macroporous Fe_3_O_4_‐PAA beads were employed for removing heavy metal ions. The macropores and abundant AA moieties rendered these materials with enhanced mass transfer and higher negative charges to effectively adsorb the cationic organic (CV) and inorganic (Pb^2+^) pollutants with maximum adsorption capacities of 80 and 291 mg g^−1^ (Figure 16b). These beads were mechanically stable without and did not show Fe leaching in the aqueous media, thereby excluding the chance of secondary pollution.^[^ ^103^ ^]^

Amino‐, epoxy‐, and carboxyl‐functionalized silica foam were prepared via one‐pot interfacial sol–gel reaction within hyperbranched PEOS‐stabilized surfactant‐free HIPE templating. Sodium polyacrylate‐functionalized silica foam was found to be efficient in removing Cu^2+^ ions with an adsorption capacity 226 mg g^−1^.^[^ ^172^ ^]^ Chromium (Cr) has extensively been used in electroplating, metallurgy and in the manufacturing of pigments, paints, pulp, papers, and preservatives. It may come into the environment via discharge from industry and sewage as well as from erosion of natural deposits. In nature, chromium exists in several oxidation states (II–VI) but commonly occurs in the trivalent and hexavalent states. The acceptable limit of Cr in drinking water established by WHO and USEPA is 0.05 and 0.1 mg L^−1^, respectively. Cr(VI) is considered to be even more toxic (about 100 times) than that of Cr(III) due to its smaller size, greater solubility, and higher mobility. Cr(VI) exists as oxyanions (HCrO_4_^−^, CrO_4_^2−^, and Cr_2_O_7_^2−^) in water, which becomes mutagenic and carcinogenic to humans beyond its safe limit of 0.05 mg L^−1^ in drinking water according to WHO and USEPA.^[^ ^266^, ^269^, ^271^, ^273^, ^274^ ^]^

Hierarchically porous amino‐functionalized organosilica monoliths, prepared via a single‐step emulsion templating technique, removed Cr(VI) ions by 92.8% from aqueous solution at RT. The removing efficiency was reduced to 85.1% after five cycles. The working capacity investigated was 4.25 kg g^−1^ for a packed column (internal diameter 1.3 cm, packing height 0.5 cm). This was based on an initial concentration of 0.2 mg L^−1^ that met the standard concentration limit set up by the WHO and USEPA after treatment. This study demonstrated an effective way to prepare emulsion‐templated porous adsorbents for the large‐scale practical environmental applications.^[^ ^275^ ^]^

Hyperbranched PEI‐tethered hierarchically macroporous beads, prepared via surfactant‐aided Pickering emulsion templating technique, were used to effectively adsorb Cr(VI) and CR ions/molecules with adsorption capacities of 141 mg g^−1^ and 37 mg g^−1^, respectively (Figure 16c). The hierarchical macroporosity, high charge density, and diverse anchoring sites accounted for their better remediation characteristics. The interaction of AO NPs with PAA excluded the risk of secondary contamination while the mechanical robustness of PAA‐AO‐PEI beads paved their way for practical applications.^[^ ^201^ ^]^

##### Arsenic Ions

The word arsenic was derived from the Greek arsenikon that means bold, valiant, or potent. As is the heavy metal found everywhere in the earth's crust. The introduction of As into the human may be via different pathways such as air, food, and particularly drinking water. The As is categorized as the priority issue among other highly toxic substances by WHO and USEPA when present above the acceptable limit of 0.01 mg L^−1^. White arsenic (As_2_O_3_) is known to be the king of poisons. The inorganic As species are more toxic than those of the organoarsenic compounds, whereas inorganic arsenite [As(III)] species are more toxic as compared to the arsenate [As(V)] species. The toxicity order of arsenic species is arsenite > arsenate > monomethylarsonic acid > dimethylarsinic acid > trimethylarsinoxide. The most common species of arsenic are As(III) and As(V). The presence of arsenite (H_3_AsO_3_) and arsenate (HAsO_4_^2−^) species at a neutral charge makes it difficult to remove arsenic from water. The inorganic arsenic is normally oxidized to As(V) that easily accumulates in the human body owing to its high mobility in various water streams.^[^ ^276^ ^]^

Hydrated ferric oxides NPs‐embedded polymerized HIPEs of AMPS were examined to remove arsenate from aqueous arsenic pentoxide solution (Figure 16d). A maximum water holding capacity of over 4000 wt % and arsenic removal efficiency of 50% (on average) from 4.5 ppm solution in a batch removal process were recorded. This was 13% better than the polymer polyHIPEs without hydrated ferric oxide NPs. The improvement in removal efficiency was attributed to the ionic interactions between the arsenic species and the hydrated ferric oxide NPs. Furthermore, the polymeric hosts were beneficial for the easier recovery of the porous adsorbents after tests.^[^ ^198^ ^]^ Ag NPs‐decorated emulsion‐templated hierarchically porous PVI beads were used to treat water contaminated with inorganic, organic, and biological water pollutants. These PVI‐Ag NCs removed As^3+^ at a capacity of 333 mg g^−1^ and also showed a good performance for eriochrome black T (81 mg g^−1^).^[^ ^102^ ^]^

##### Ag^+^ Ions

The advent of nanotechnology has tremendously increased the application of NPs in electronics, cosmetics, drug delivery systems, etc., and in turn contamination of water resources. Many conventional nano/microsized materials have been used as sorbing media in wastewater/groundwater treatment applications, but again cause secondary contamination owing to their complicated recovery on account of their very small sizes. In this regard, porous macrosized emulsion‐templated materials have emerged as exciting new materials to serve the same purpose effectively. Ag and Ag NPs are being used in a range of applications and consumer products owing to its antimicrobial properties. Yet, it is known as an emerging environmental contaminant. The tolerable Ag level in drinking water established by WHO and USEPA is 0.1 mg L^−1^.^[^ ^267^ ^]^

In recent years, only a few researchers have studied the way of separation of NPs from water. In this regard, PEGMA‐based polyHIPE monoliths were modified with various amines and evaluated for the removal of Ag^+^, Cu^2+^, and Cr^3+^. Overall, these monoliths showed better adsorption for Ag^+^ as compared to Cu^2+^ and Cr^3+^. The maximum amounts of Ag^+^, Cu^2+^, and Cr^3+^ adsorbed were reported to be 9.05, 4.99, and 2.92 mmol g^−1^, respectively.^[^ ^156^ ^]^ The highly porous and stable (3‐acrylamidopropyl)‐trimethylammonium chloride‐based cationic polyelectrolytes prepared via the O/W HIPEs showed the total ion exchange capacity of 3.53 mmol of AgNO_3_ per gram of dry resin and water uptake of up to 95 g g^−1^, which was superior to many benchmark polyHIPEs with the same cationic functional groups. The materials also exhibited the promising uptake capacities for erythrosine dye predominantly via chemisorption (Figure 16e).^[^ ^146^ ^]^

##### Li^+^ Ions

Lithium plays a matchless role in contemporary technologies of pharmaceuticals, battery, lubricants, etc. Keeping in view its rapid consumption worldwide, the extraction and recovery of lithium from brines and waste batteries has received much attention. In this context, functional macroporous GMA‐based microspheres with multiple interconnected chambers fabricated via a multiple emulsion templating technique were used for selective capture of Li^+^ ions with an adsorption capacity of 38.13 mg g^−1^. This performance was attributed to their macroporous structure and immobilized crown ethers. However, their adsorption capacity decreased to 91.14% after five cycles of adsorption/desorption.^[^ ^277^ ^]^ The halloysite nanotubes‐stabilized Pickering HIPEs‐derived Ag‐modified PS sulfonate monoliths were employed to selectively bind Li^+^ in the presence of K^+^, Mg^2+^, and Na^+^ ions. The process showed fast binding kinetics and Langmuir adsorption capacities of 59.85, 35.06, and 27.09 mg g^−1^ at 15, 25, and 35 °C, respectively. The adsorbent maintained an adsorption efficiency of 80.71% after seven sequential cycles of adsorption–regeneration. These materials also displayed antifouling performance.^[^ ^204^ ^]^

##### Radioactive Ions

The natural geological processes, catastrophic incidents, usage of nuclear powered electricity, and production of nuclear weapons have augmented the release of radioactive constituents into the environment. Radioactive elements including uranium and plutonium, iodine, cesium, strontium, etc., are the worst water contaminants. The handling and processing of radioactive ions is a risky and expensive practice owing their toxic and hazardous nature. It is thus urgent to hunt for the development of new materials and technologies.^[^ ^267^, ^278^ ^]^

Uranium is an actinide element that exists as an oligomeric species in aqueous medium. It forms uranyl ion (UO_2_^2+^) at low pH (<2) and hydrolyzed products or partially hydrolyzed products such as (UO_2_)_3_(OH)_7_, (UO_2_)_4_(OH)_7_, UO_2_(OH)^+^, (UO_2_)_3_(OH)_5_^+^, and (UO_2_)_2_(OH)_2_^2+^ at high pH values. The U(VI) may cause liver damage and cancer after long‐term exposure. The permissible level of U in drinking water set by WHO and USEPA is 0.015 and 0.03 mg L^−1^, respectively.^[^ ^267^, ^271^, ^274^ ^]^

**Figure 17:**
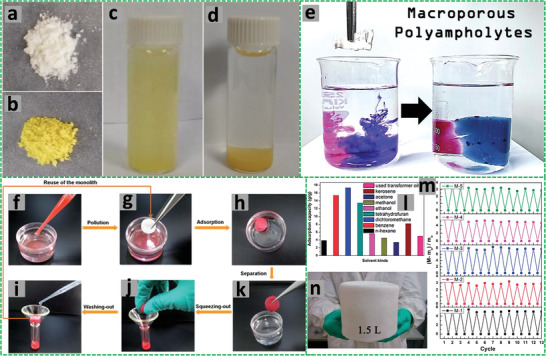
Images show amidoxime‐modified hollow porous melamine resin polymer microspheres before adsorption (a,c) and after adsorption (b,d) of U(VI). Reproduced with permission.^[^ ^165^ ^]^ Copyright 2020, Elsevier. e) Photograph shows dyes removal by macroporous amphoteric polyelectrolyte monolith. Reproduced with permission.^[^ ^147^ ^]^ Copyright 2020, Elsevier. f) Water polluted with gasoline and dyed with 1,6,7,12‐tetra‐*tert*‐butylphenoxyperylene‐3,4,9,10‐tetracarboxylic dianhydride, g) addition of porous PS monolith to gasoline mixture, h) adsorption of gasoline by monolith, i) monolith recovery after adsorption, j) pressing of monolith to remove gasoline, and k) washing of monolith for reuse. Reproduced with permission.^[^ ^109^ ^]^ Copyright 2013, Royal Society of Chemistry. The graphs show l) adsorption capacities of LMMG‐based W/O gel emulsion‐templated porous polymeric monolith (M‐3), m) regeneration studies of monoliths prepared via different methods (M‐1–M‐5) using kerosene oil as an example, and n) a digital photograph of M‐3 monolith. M‐1–M‐5 indicates monoliths prepared with different silanes. Adapted with permission.^[^ ^181^ ^]^ Copyright 2014, Royal Society of Chemistry.

To extract UO_2_^2+^, hollow porous microspheres with amidoxime groups prepared via silica NPs‐stabilized CO_2_ Pickering emulsion templating exhibited excellent potential with a maximum adsorption capacity of 553.3 mg g^−1^ in 1 h at 298 K even in the presence of high concentrations of Ca^2+^, Mg^2+^, Na^+^, Zn^2+^, and other ions (**Figure** **17**a–d). The impressive adsorption capacity, fast equilibrium rate, and high selectivity were resulted from the well‐defined hollow porous morphology, uniform size, and high‐density of amidoxime groups.^[^ ^165^ ^]^

Pu is an artificial element. Its radioisotopes such as ^239^Pu and ^240^Pu are highly radiotoxic, biotoxic, and long‐lived hazards, which are abundantly found in radioactive wastes and spent nuclear fuel and released into the environment by human activities. It thus highlights the need for key control on environmental mobility of Pu by using highly effective materials for its removal.^[^ ^279^ ^]^ In this regard, P4VP grafted P(St‐*co*‐DVB) polyHIPEs showed good promise as the ion exchange column prototypes for Pu^4+^ separation. A greater ion exchange capacity per unit volume for Pu separation than those of the commonly used materials (e.g., Reillex HPQ resin), with a narrower elution profile, was recorded. This high‐performance separation was believed to be due to the convective mass transfer and exposed ion‐exchange sites (quaternized pyridine) on the surface of their large pores, as compared to the diffusion into the smaller pores on a resin beads surface.^[^ ^115^ ^]^ 4VP‐functionalized P(St‐VBC) foams were reported for their heavy metals separation capability. In general, these monoliths demonstrated improved and better uptake kinetics (moderate for Pu^4+^ while high for iron ions) and distribution coefficients than those of the resins with similar structures. Furthermore, the foams exhibited good flow properties in column and were found durable to bear pressure up to 40 psi.^[^ ^114^ ^]^

The radioactive by‐products such as Rb^86,87^ and Cs^134,137^ nuclei, discharged by the nuclear power plants also contaminate water bodies. Many materials such as emulsion liquid membranes, PAM‐supported materials, certain oxides, and composites, have been used, though the meaningful remediation of such types of radionuclides has recently been achieved by emulsion‐templated porous materials.^[^ ^280^ ^]^ By way of example, the hybrid CMC‐MMT‐PAM porous adsorbents showed good potential in removing radioactive Rb^+^ and Cs^+^ ions with fast adsorption equilibrium time (30 min) and high adsorption capacities of 178 and 266 mg g^−1^, respectively. The complexation between COO^−^ and Rb^+^ or Cs^+^ was considered as the main driving force responsible for the adsorption.^[^ ^197^ ^]^ The superporous Yeast‐PAA monoliths, prepared through eco‐friendly yeast‐stabilized O/W Pickering HIPEs templating, offered fast capture rate and strong adsorption for radioactive Rb^+^, Cs^+^, and Sr^2+^ ions with saturated capacities of 180, 230, and 167 mg g^−1^, respectively. This performance was attributed to a large number of interconnected pores and carboxylate moieties. Furthermore, these porous adsorbents exhibited good regeneration and reusability up to five adsorption‐desorption cycles while maintaining its high adsorption performance.^[^ ^155^ ^]^

#### 3.1.2 Adsorption of Organic Pollutants

##### Dyes

Dyes are intensely colored organic compounds widely used in textiles, printing, paper, food, drugs, cosmetics, paints, rubbers, and plastics industries, which adversely affect human health. These organic environmental pollutants such as azo, sulfur, cyanine, anthraquinone, and heterocyclic dyes are stable to heat, light, and oxidizing agents and highly toxic to living organisms due to their less or non‐biodegradability.^[^ ^281^ ^]^ The activated carbon, CNTs, silica, and many others materials have been tested to remove organic dyes though; emulsion‐templated porous adsorbents offer convincing solution for the efficient dye removal.

Gemini surfactant‐stabilized porous CS‐PAM monoliths were able to remove MB, which is a widely reported cationic dye. These emulsion‐templated porous monoliths exhibited an adsorption capacity of 454 mg g^−1^.^[^ ^139^ ^]^ Hierarchically porous UiO‐66‐PAM composite monoliths exhibited relatively high stresses at 82% strain and recovered their shape quickly. This provided mechanical stability and facile recycling when evaluated for the removal of MB. The adsorption capacity for MB was found to be 50 mg g^−1^ at a faster adsorption rate.^[^ ^142^ ^]^

Macroporous polymer monoliths prepared from DMAEMA and HEMA could adsorb both the cationic (MB) and anionic (MO) dyes from water with adsorption capacities of 6.5 and 1.6 mg g^−1^ within 120 min, respectively.^[^ ^157^ ^]^ When the pH of the MB solution increased, PDMAEMA was deprotonated, resulting in increasing MB adsorption. For anionic dye MO, PDMAEMA was protonated with the decrease of pH, leading to enhanced interaction with MO and higher adsorption.^[^ ^157^ ^]^ The amino‐modified silica foams exhibited fast adsorption of sunset yellow with an excellent uptake capacity (1213 mg g^−1^) and remarkably short adsorption equilibrium time (≈5 min). When the silica foam was functionalized by polyacrylate, the removal of MB at an adsorption capacity of 280 mg g^−1^ was achieved.^[^ ^172^ ^]^ Three forms of porous polyampholytes bearing cationic (–NR_3_^+^) and anionic (–SO_3_^−^) groups were constructed and evaluated for the removal of MB (concentration 100 ppm) and erythrosine (anionic dye, 100 ppm) in a batch reactor. These three different framework structures of porous polyampholytes performed differently for dyes removal. For both dyes, the copoly‐ampholyte form performed quite poorly and considerable improvement was observed for the mixed‐amphoteric form, while the bilayered‐amphoteric form showed the best performance with the adsorption capacity of 88 mg g^−1^ for MB, 57 mg g^−1^ for erythrosine (Figure 17e).^[^ ^147^ ^]^

Porous PAM microspheres, prepared via O/W/O emulsion templating, were evaluated for the removal of cationic dyes MB and MV. High adsorption capacities; 669 mg g^−1^ for MB and 750 mg g^−1^ for MV, were achieved in a short period of time (30 min) at a controlled temperature of 25 °C and pH 8. The adsorption capacity almost increased linearly with increasing pH 2–4 due to the increased negative zeta potential.^[^ ^169^ ^]^ A functionalized PAM with TiO_2_ composite, TiO_2_/P(AM‐*co*‐AMPS), was found to be a more efficient adsorbent for MB with an adsorption capacity of 1.66 g g^−1^. The pollutant could be removed in a wide pH range of 3–10. It was proposed that the electrostatic attraction between −SO_3_^−^ and MB was the driving force for the high adsorption capacity.^[^ ^134^ ^]^

##### Pesticides

The pesticides are extensively being used in agriculture to kill pests for improving production yield. However, exposure to pesticides may cause eye irritation, nausea, skin diseases, Parkinson's disease, and cancer. These pesticides usually contaminate water bodies that are in turn seriously threatening the environment and ecosystem.^[^ ^282^ ^]^ Among different types of materials including composite material, ion exchange resins, CNTs, graphene, and nanocrystalline metal oxides, emulsion‐templated porous materials have recently been employed for the remediation of pesticide‐contaminated water.

Molecularly imprinted multi‐hollow microspheres were prepared via lignin (hydrophilic and lipophilic) particles‐stabilized Pickering W/O/W emulsion templating. These microspheres showed promising recognition and subsequent removal of *λ*‐cyhalothrin. An adsorption capacity of 24.79 mg g^−1^ was achieved in an equilibrium time of 3 h. These microspheres could be regenerated by washing with methanol/acetonitrile and reused many times, with a loss of 8.11% affinity after seven testing cycles. This approach has demonstrated the preparation of porous adsorbents for selective recognition/detection of targeted molecules.^[^ ^167^ ^]^

##### Herbicides

A triazine herbicide, atrazine, is the endocrine‐disrupting chemical typically used in agricultural production and migrates to groundwater like the other organic pesticides owing to its mobility and high chemical stability, thereby causing water pollution and threatening human health. Likewise, the trifluralin is extensively used dinitroaniline herbicide that may persist in the environment and cause considerable ecotoxicity particularly to the aquatic organisms.^[^ ^283^ ^]^

Piperazine‐functionalized P(St‐*co*‐NPA)‐based porous polyHIPEs were reported to remove atrazine from water. Because of their high porosity and good accessibility to active sites, these polyHIPEs performed more efficiently to remove atrazine (68% in 6 h and 98% in 48 h) from aqueous solutions than those of their chemically analogous beads, which showed a relatively lower atrazine removal efficiency (15% in 6 h and 98% in 144 h).^[^ ^117^ ^]^ The porous carbon prepared from GO and silica NPs‐laden P(DVB‐EHA) was found to be highly efficient in adsorbing trifluralin, a herbicide causing harm to aquatic life, with the removing efficiency up to 100%. This was attributed to the *π*–*π* and hydrophobic interactions between trifluralin and the carbon matrix. A syringe packed with such porous carbon was then used to enrich trifluralin from soil samples by solid phase extraction and detect the level of the herbicide in the soil.^[^ ^256^ ^]^

##### Antibiotics

Antibiotics are widely used to treat/prevent bacterial infections by killing the bacteria or inhibiting their growth or reproduction. On the other hand, they may be introduced into the aquatic environment through food chains/webs. They are currently emerging as prevalent pollutants on account of their increased consumption for the human and animals’ disease treatment and agricultural activities. The alarming levels of antibiotics in municipal and medical wastewater as well as in surface and groundwater may be highly toxic to the aquatic ecosystems.^[^ ^284^ ^]^

TiO_2_/P(AM‐*co*‐AMPS) could remove TC (a cationic antibiotic) efficiently with an adsorption capacity of 1.13 g g^−1^.^[^ ^134^ ^]^ Porous magnetic Fe_3_O_4_@HKUST‐1‐embedded P(EHA‐DVB‐MMA) monolithic composites could extract TC from milk, chicken egg, muscle, and kidney samples with high column capacity owing to the improved *π*–*π* and electrostatic interactions and hydrogen bonding. The limits of detection and the limits of quantification were in the range of 1.9–4.6 ng cm^−3^ and 5.5–13.9 ng cm^−3^ for milk and egg samples and 1.8–3.7 ng g^−1^ and 5.3–13.0 ng g^−1^ for muscle and kidney samples of chicken, respectively. The recoveries of TC from spiked food samples ranged from 86.6% to 110.7% with relative standard deviations less than 7.0%.^[^ ^215^ ^]^

Magnetic CS‐*g*‐PAMPS porous adsorbents prepared by templating the modified‐Fe_3_O_4_ NPs‐stabilized Pickering HIPE displayed high efficiency in removing the antibiotics including TC and CTC in equilibrium time of 90 and 50 min with adsorption capacities of 807 and 877 mg g^−1^, respectively. The high uptake capacities were mainly attributed to the electrostatic attraction between the adsorbents and the targeted adsorbates. Furthermore, the tested adsorbents can be regenerated for five consecutive cycles without any significant adsorption capacity losses.^[^ ^193^ ^]^

##### PAHs

PAHs are environmental pollutants that come from anthropogenic as well as natural sources. Owing to their mutagenic and carcinogenic effects, 16 PAHs have been enlisted as priority pollutants by USEPA. Despite being lipophilic in nature, PAHs compounds may enter into industrial wastewater and thus contaminate drinking water. Additionally, they also exist in the air and cause air pollution. Therefore, the effective clean‐up of such PAHs is needed on urgent basis.^[^ ^285^ ^]^

In this context, rGO‐polyHIPE hybrids with hydrophobic nature and open‐cell structure showed good adsorption performance for PAHs with a saturated adsorption capacity of 47.5 mg g^−1^ in an equilibrium time of 8 h. These materials could be reused for ten consecutive adsorption–desorption cycles without any significant loss of adsorption capacities. Importantly, these hybrid materials were flexible and robust.^[^ ^254^ ^]^

##### Liquids with Odor/Warfare Agents

The chemical warfare agents are known to be extremely toxic and unsafe to retain in the environment. The high‐temperature incineration, caustic bleaching, metal‐catalyzed hydrolysis, supercritical water‐induced oxidation, and bioremediation have emerged as promising methods to remediate chemical warfare agents, but enzymes instability under harsh conditions, high energy costs, and generation of secondary pollution undermine their suitability for practical applications. In this context, the development of new practically worthwhile materials are highly required.^[^ ^5^ ^]^

Highly macroporous PVBC monolithic columns were reported for the effective removal of acid chloride because of their interconnected porous structure and good permeative properties. These tris(2‐aminoethyl)amine ‐functionalized polyHIPE monolithic columns showed the capability of removing 94.7%, 97%, 97.5%, and 98% of benzoyl chloride from DCM solution after 1st, 2nd, 3rd, and 4th cycles, respectively.^[^ ^116^ ^]^ Porous P(St‐EGDMA) prepared from an amphiphilic block copolymer‐stabilized gel emulsion could effectively remove a wide variety of volatile organic compounds including benzene, toluene, xylene, ethylbenzene, chloroform, THF, acetone, formaldehyde, hexane, etc. These porous polymers could be reused for ten cycles of adsorption and desorption with nearly no change in adsorption capacity. The adsorption capacities of these monoliths, 907 mg g^−1^ for benzene and 777 mg g^−1^ for toluene, were quite high.^[^ ^120^ ^]^

The P(St‐*co*‐VBC‐*co*‐DVB) HIPE‐templated monoliths were found to immobilize chemical warfare agents (CWAs) such as sulfur mustard, isopropyl methyl phosphonofluoridate, diethylaminoethyl O‐ethyl methylphosphonothioate, diethylaminoethyl O‐isobutyl methylphosphonothioate, diethylaminoethyl O‐butyl methylphosphonothioate, diisopropylaminoethyl O‐ethyl methylphosphonothioate, and bis(2‐chloroethyl) sulfide. The adsorption of these CWAs was increased by increasing the VBC content and the volume of the internal phase emulsion, which could be positively correlated with the swelling. The adsorption performance, determined by the mass of absorbed CWAs to the mass of dry porous polymer, was in the range of 40–55 for the tested CWAs. Furthermore, the materials were suitable for practical applications because of their ability to swell from both the monolithic and the compressed states.^[^ ^113^ ^]^

#### 3.1.3 Oil/Water Separation

Besides the soluble pollutants, weekly soluble or even insoluble oily pollutants also contaminate water on account of the intentional/accidental oil spill accidents and industrial discharge. The discharge of valuable non‐renewable crude oil and petrochemicals is problematic from both economic and environmental perspectives. The consumption of foods of oil‐polluted seas exposure to volatile hydrocarbons adversely affects human health. The adsorption of oil from oil/water mixture usually requires natural adsorbents including the activated carbon, but the loading capacity of such materials is too low. A great deal of research work has been dedicated to explore inventive strategies for the preparation of superhydrophobic materials with high oil/water separation ratio.^[^ ^281^ ^]^ In this connection, emulsion templating has been taken as a promising approach to fabricate porous materials with desired functional groups for the effective oil/water separation. For instance, hydrophobic polyHIPEs are well positioned as efficient adsorbents for oil uptake and oil/water separation due to their highly interconnected macropores, ultralow density, and high pore volume. The induction of elasticity, flexibility, and mechanical stability makes these polyHIPEs highly recyclable materials with easy handling and facile regeneration.

Hydrophobic P(St‐*co*‐DVB) aerogels with a water contact angle of 84.5° and improved mechanical stability by the incorporation of acrylate monomers could adsorb 13–29 times (g g^−1^) of organic solvents (i.e., toluene, phenol, *n*‐heptane, DCM) and remove oil from the oil/water mixture.^[^ ^110^ ^]^ Ultralow density porous polyHIPEs, prepared with LMMG as stabilizer, showed fast and selective adsorption of DCM (high density) and mineral oil (viscous) with capacities in the range of 2.51–20.21 g g^−1^ for various types of organic solvents and oils (Figure 17f–k).^[^ ^109^ ^]^ Another LMMG was employed as stabilizer for W/O emulsions in the preparation of porous P(*t*BMA‐DVB) monoliths. Different silanes were introduced into the continuous oil phase to improve the stability of the polyHIPEs. These composites could rapidly adsorb water‐immiscible and water‐miscible liquids including benzene, kerosene oil, used transformer oil, ethanol and THF. The adsorbents could be easily regenerated by squeezing, centrifuging, and washing, and then reused for at least 13 times with small performance decline (Figure 17l–n).^[^ ^181^ ^]^

A polyHIPE xerogel was prepared from the complexation of PPI and SPS‐*b*‐PE‐*r*‐Bt‐*b*‐PS by emulsion freeze drying. This xerogel could remove diesel rapidly from its water mixture in 20–30 s. It also displayed the potential of absorbing various types of oils including hexane, toluene, xylene, DCE, chloroform, vegetable oil, gasoline, diesel, engine oil, and crude oil, in the capacity of 20–32 g g^−1^ of the adsorbent. Impressively, this adsorbent could be recycled at least 40 times without any significant loss of the performance in the temperature range of 0–50 °C.^[^ ^126^ ^]^ A similar approach was used to prepare a macroporous sponge between SPS‐*b*‐PE‐*r*‐Bt‐*b*‐PS and PAMAM dendrimer. This hydrophobic polyHIPE exhibited a water contact angle of around 128.1° and could selectively absorb oil from oil–water mixture with the absorption capacities in the range of 12.5–25.8 g g^−1^. This material could be recycled 30 times with about 15% loss in adsorption performance.^[^ ^125^ ^]^

Fluorinated acrylate monomers were introduced in the preparation of P(St‐*co*‐DVB) polyHIPEs. The resulting materials were superhydrophobic (water contact angle 151°) and oleophilic. They could efficiently separate 95% of DCM from the oil/water mixture and reused for ten cycles without affecting separation capacity.^[^ ^122^ ^]^ A robust PDMS‐infused porous PS‐based slippery membrane system was fabricated and used to repel both water‐ and oil‐based contaminants (e.g., ink, milk, and coffee) with very low sliding angles (3.0 ± 1.3°), as well as solid contaminants (e.g., dust). Such membrane systems showed promising self‐repairing properties without obvious impact of physical scratching damage on their liquid‐repelling performance. Furthermore, the liquid repelling ability of such porous membrane systems could be recovered fully only in 10 s together with excellent durability to maintain their water sliding angle at <5.0° for about 80 cycles.^[^ ^171^ ^]^

Highly macroporous hydrophobic PU polyHIPEs prepared from mannitol within non‐aqueous HIPEs showed good potential for oil spill reclamation with regeneration ability through centrifugation. The absorbed oils could be removed at a high recovery rate of ≈85%. Furthermore, the regenerated monoliths could be recycled 20 times without any significant loss of uptake capacity and recovery rate.^[^ ^128^ ^]^ The PU polyHIPEs fabricated from non‐aqueous emulsions with a reactive block copolymer as stabilizer showed a good mechanical stability with Young's moduli of 0.3–4.4 MPa. This facilitated the reuse of the PU polyHIPEs as adsorbents for halogenated liquids. The absorption capacities of 36.0 g g^−1^ for chloroform and 27.3 g g^−1^ for DCE were achieved at fast rates, with a short time of 2 min to reach half equilibrium uptakes.^[^ ^130^ ^]^ A robust cellulose‐based PU polyHIPE was found to be effective in oil/water separation. The oil uptake capacities, 22 cm^3^ g^−1^ for DMSO and 9.5 cm^3^ g^−1^ for non‐polar solvents, were observed. The analysis showed that the adsorption was based on void filling of the polyHIPE without swelling‐induced expansion.^[^ ^129^ ^]^

**Figure 18:**
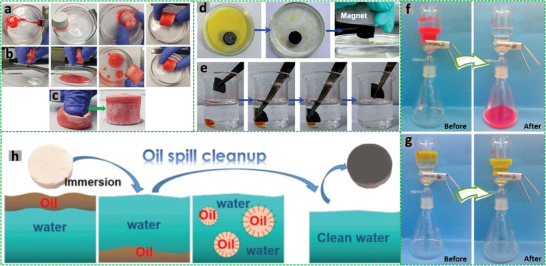
Photographs demonstrate a) sorption/compression/resorption, b) recovery, and again resorption of the crude oil from oil–water mixture by using emulsion‐templated covalently bonded silica−polymer composites, and c) flexible/compressible nature of the adsorbent. A red dye is added into the crude oil for easy observation. Reproduced under a Creative Commons Attribution 4.0.^[^ ^190^ ^]^ Copyright 2017, The Authors(s), published by Springer Nature. Photographs show the removal of d) diesel and e) tetrachloromethane (stained with Sultan VI) from water. Reproduced under a Creative Commons Attribution‐NonCommercial 3.0 Unported Licence.^[^ ^196^ ^]^ Copyright, The Author(s), published by the Royal Society of Chemistry. Images of CO_2_‐controlled O/W separation. Separation of f) water/chloroform (red) using dried membrane and g) water/hexane (yellow) by CO_2_‐treated membrane. Reproduced with permission.^[^ ^119^ ^]^ Copyright 2017, American Chemical Society. h) Schematics illustrates oil absorption from water surface, underwater oil, and emulsified oil by SPS monolith. Reproduced with permission.^[^ ^112^ ^]^ Copyright 2019, American Chemical Society.

Porous silica–polyHIPE monoliths, where the silica aerogel was covalently bonded to the silanol groups of the polyHIPE matrix, were found to be highly efficient for the adsorption of oil from the water/oil mixtures. The materials displayed good mechanical stabilities with a Young's modulus of 10.47 MPa and original shape restoring ability after applying ≈76% compression. These highly flexible and stable porous materials could be regenerated easily by squeezing the swollen adsorbent (**Figure** **18**a–c).^[^ ^190^ ^]^ The sorption/desorption capacity for crude oil was recorded as ≈16 g g^−1^. This composite adsorbent could be reused for 25 sorption cycles with similar adsorption capacity.^[^ ^190^ ^]^

Magnetic porous Fe_3_O_4_/PS composites, prepared from the W/O emulsion co‐stabilized by Fe_3_O_4_ NPs, were used to adsorb oils (i.e., diesel, edible oil, lubricating oil, and tetrachloromethane) from their mixtures with water. The adsorption process was effective for both oil on top of water and underwater (Figure 18d,e).^[^ ^196^ ^]^ The oil adsorption capacity of this material was approximately in the region of 8–16 g g^−1^ based on the mass of dry adsorbent. Notably, these materials could be recycled 10 times without any significant loss in the adsorption capacity.^[^ ^196^ ^]^

Highly porous poly(styrene‐*co*‐2‐*N*,*N*‐diethylamino)ethyl methacrylate) [P(St‐*co*‐DEAEMA)] membranes showed their promise in smart oil–water separation. These polymer membranes not only facilitated fluid penetration through their open‐cell structures, but also the combination of CO_2_‐switchable functionality and porous microstructure rendered them with CO_2_‐switchable wettability between hydrophobic/superoleophilic and hydrophilic/superoleophobic. This was achieved via using CO_2_ treatment or bubbling by thoroughly drying the membrane first and then immersing it into the water. Such membranes were efficient for the gravity‐driven CO_2_‐controlled oil/water separation, wherein the oil (chloroform) selectively passed through the membrane and separated out from water. Interestingly, a reversed separation of water and oil (hexane) could also be realized after treating the membranes with CO_2_ (Figure 18f,g).^[^ ^119^ ^]^

The sPS polyHIPEs with nanofibrous pore walls were found to be highly efficient as oil adsorbents. The hierarchical porosity, superhydrophobicity, high specific surface area, and high strength of this material provided additional benefits for fast oil adsorption. The adsorption of oils on the water surface (toluene), underwater (chloroform), and within O/W emulsions (toluene‐in‐water stabilized by Tween 80) was demonstrated (Figure 18h). Since the adsorption of toluene and chloroform dyed with oil red O could be easily seen, the adsorption of oil phase from the O/W was confirmed by microscopic observation (i.e., the disappearance of oil droplets). The uptake capacities of a variety of organic solvents and oils were determined by soaking the sPS polyHIPEs in pure oil phase for 24 h, with the values in the range of 28.0–66.7 g g^−1^ depending on the type of oil and the composition of sPS polyHIPE. The sPS polyHIPE adsorbent was highly reusable. The loss of 10% in diesel adsorption capacity was recorded after at least 20 cycles of uptake‐centrifugation measurements.^[^ ^112^ ^]^

On the basis of the *π*‐conjugation, hydrophobic nature, and large available surface of the graphene walls, highly porous 3D graphene foams showed great potential to adsorb oils (toluene, hexadecane, and olive oil) from water and aqueous solutions. The high thermal stability of the graphene foams meant that they could be recycled after oil uptake by direct combustion in air.^[^ ^258^ ^]^ A low density rGO‐based porous material (4.3 mg cm^−3^, 99.8% porosity) were found to be highly efficient in absorbing oils with the intake capacity in the range of 113–276 g g^−1^.^[^ ^259^ ^]^ When a lighter rGO‐based porous material (density 1.5 mg cm^−3^) was used, the absorption capacity of motor oil was increased from 276 to 605 g g^−1^. These rGO‐based materials were found chemically and mechanically stable, as demonstrated by their exposure to the oils for a few weeks. The adsorbed oil could be efficiently “squeezed out” of the adsorbent by compressing it as far as over 95% of its initial height. The compressed adsorbent could be reused simply by soaking in the oil phase again for at least six cycles, with the adsorption capacity maintained at >95%.^[^ ^259^ ^]^

#### 3.1.4 Antibacterial Treatments

Various types of pathogenic microbes like virus, bacteria, fungi, algae, amoebas, and planktons present in water as soluble, colloidal, and/or suspended form are the main cause of several diseases. Many materials such as biopolymers (e.g., CS, etc.), metal NPs (e.g., gold, Ag, copper, zinc, etc.), and proteins have been used to inhibit the bacterial growth or reproduction or to kill the bacteria. However, the currently used antibacterial compounds present some drawbacks. For instance, the use of antibiotics has induced the production of antibiotic‐resistant bacteria or antibiotic resistance genes, whereas the proteins are highly prone to environmental conditions and lose their antibacterial activity in the long‐term on account of the agglomeration and conformational changes.^[^ ^286^ ^]^

**Figure 19:**
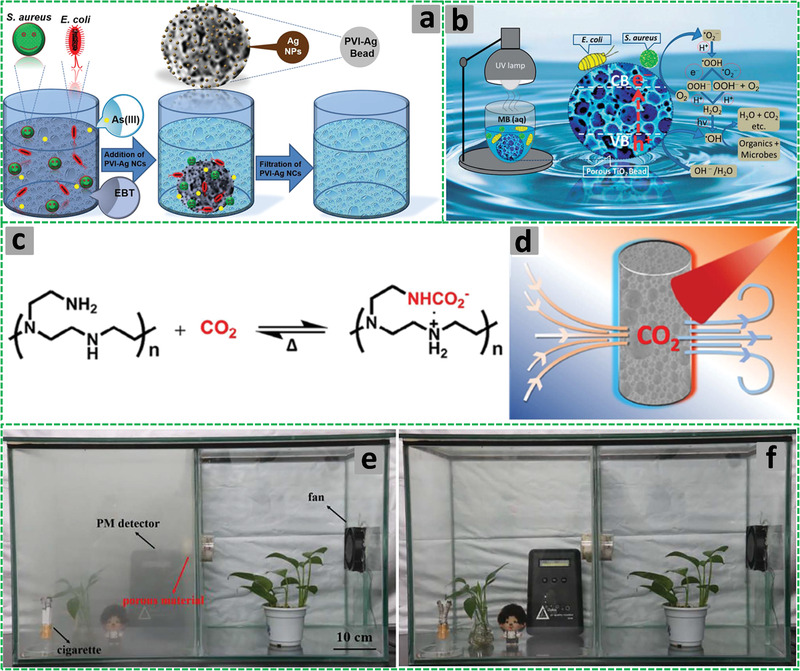
a) Cartoons illustrate the use of the Ag NP‐decorated emulsion‐templated hierarchically porous PVI beads to treat water contaminated with inorganic, organic, and biological pollutants. Reproduced with permission.^[^ ^102^ ^]^ Copyright 2017, American Chemical Society. b) Schematics of the plausible mechanism for photodegradation of organic dyes and microbes by porous TiO_2_ beads. Reproduced under a Creative Commons Attribution‐NonCommercial 3.0 Unported Licence.^[^ ^101^ ^]^ Copyright 2018, The Author(s), published by the Royal Society of Chemistry. c) Reaction between PEI‐based adsorbent and CO_2_. d) Representative illustration of light‐triggered CO_2_ breathing. Reproduced with permission.^[^ ^288^ ^]^ Copyright 2017, American Chemical Society. e) Photographic demonstration of the compartment with cigarette smoke containing polluted air and the compartment with clear air. The two compartments are connected with a filter packed with amino‐functionalized P(St‐*co*‐MMA). f) After turning on the fan, the particulate matter is removed after passing the polluted air through the filter, indicated with the red arrow. Reproduced with permission.^[^ ^187^ ^]^ Copyright 2018, Elsevier.

Anti‐bacterial agents can also be incorporated into the polyHIPEs to achieve both the adsorption of pollutants and the killing of bacteria. Ag nanomaterials are widely used for antibacterial or antimicrobial treatments.^[^ ^287^ ^]^ Mudassir et al. reported the in situ formation of Ag NPs within porous PVI beads. The Ag‐PVI composite beads could effectively remove As^3+^ and eriochrome black T. They were also found to kill *Escherichia coli* (Gram‐negative) and *Staphylococcus aureus* (Gram‐positive) bacteria with bactericidal efficiencies of ≈96% and ≈100%, respectively (**Figure** **19**a).^[^ ^102^ ^]^ These beads could have a great potential for real‐life applications owing to their macrosize (1.80 ± 0.15 mm), hierarchical porosity, easy handling and separation, and long‐term stability.^[^ ^102^ ^]^ In a further study, emulsion‐templated Ag‐PVSA beads were utilized to adsorb Hg^2+^ and Rhodamine B with maximum uptake capacities of 191 and 53 mg g^−1^, respectively, and effectively kill *S. aureus* and *E. coli* bacteria with bactericidal efficiencies of 98.39% and 100%, respectively.^[^ ^98^ ^]^

### 3.2 Catalytic Degradation/Oxidation of Organic Pollutants

In addition to removing pollutants by adsorption, degradation or oxidation of inorganic, organic, and biological pollutants is an imperative theme for environmental remediation. Photocatalytic degradation via various catalytic mechanisms of water pollutants is one of the ideal solutions whereby large organic pollutant molecules are degraded into nontoxic small molecules, which can be released directly. This catalytic degradation or oxidation approach removes the need of regenerating adsorbents and recycling the recovered pollutants.

Different metals/metal oxide NPs such as gold, Ag, platinum, palladium, and bimetallic composites have widely been employed as proficient catalysts to degrade aromatic nitro compounds and organic dyes. However, small size, high surface energy, and aggregation tendency undermine catalytic efficiency, stability, handling, and recovery of these nanocatalysts. Likewise, the photocatalytic activity of substrate‐supported metals and metal oxide‐based catalytic materials decreases mainly on account of the adsorptive nature of these supports (e.g., stainless steel, quartz, glass, silica, AO, carbon, clay, and polymer). Also the leaching of catalysts from supporting materials causes secondary pollution. To circumvent these pressing concerns that limit practical worth of such catalysts, emulsion templating has emerged as a cutting‐edge strategy to fabricate hierarchically porous materials, which can be easily processed and subsequently recovered for recycling, thereby providing cost‐effective benefits and alleviating the risk of secondary pollution.^[^ ^101^, ^281^ ^]^

There are many examples of emulsion‐templated porous materials used for the heterogeneous catalysis.^[^ ^152^, ^203^, ^211^ ^]^ For example, emulsion‐templated magnetic Fe_3_O_4_ NPs‐impregnated PAM composite monoliths with different MOFs (HKUST‐1, MOF‐2, UiO‐66, Fe‐MIL‐101, and Fe‐MIL‐101‐NH_2_Fe_3_O_4_) showed promising applications in heterogeneous catalysis because of their high surface areas and fast mass diffusion, resulted from the presence of micropores and macropores, respectively. Among them, HKUST‐1@PAM composites were found to catalyze the isomerization of *α*‐pinene oxide with better performance (conversion 90% and selectivity 70%) than that of bulk HKUST‐1 (conversion 62%, selectivity 63%).^[^ ^211^ ^]^ The superior catalytic performance of the polyHIPE composites, as a result of catalysts supported by polyHIPEs, may be similarly translated to catalytic degradation of organic pollutants, which is the focus of the below discussion.

The hierarchically porous Ag‐incorporated m‐MOP particle‐decorated PAM monolith was prepared via the polymerization of the O/W Pickering HIPEs followed by washing and freeze drying.^[^ ^205^ ^]^ Due to their water wettability, hierarchical porosity, and good compressive mechanical properties, the monoliths were employed as a heterogeneous catalyst to reduce 4‐nitrophenol in aqueous medium with a reaction rate constant of 0.037 min^−1^, seven times higher than that of the m‐MOP/Ag particles. The rate constant could be further enhanced up to 0.197 min^−1^ with the compression and release of the monoliths (15 s per cycle).^[^ ^205^ ^]^

Porous Fe_3_O_4_‐PAM beads were prepared by an O/W/O sedimentation process using O/W HIPEs stabilized by Tween 85 and Fe_3_O_4_ NPs (100–300 nm) and then used as catalysts for Fenton reactions. The catalytic reactions were carried out by passing the aqueous MO solution through the chromatography column packed with these composite beads. The results showed that these beads were able to decompose 99.6% of MO. These composites beads were reusable and could be used as Fenton catalysts for decomposition of other organic pollutants.^[^ ^195^ ^]^

Hierarchically porous nanostructured TiO_2_ beads were highly effective for photodegradation of MB with a rate constant of 0.05 min^−1^ and efficient disinfection of *E*. *coli* and *S*. *aureus* under UV light (Figure 19b).^[^ ^101^ ^]^ The millimeter size and mechanical stability of these TiO_2_ beads have many advantages over traditional TiO_2_‐based NCs and slurry systems to address the inherent bottlenecks of secondary contamination, difficult operation, and energy‐intensive post‐recovery, which are believed to be the obstacles to developing practically viable water treatment technologies.^[^ ^101^ ^]^

Highly porous PAE‐based *π*‐conjugated microcellular polymers, prepared by the Pd and Cu‐catalyzed Sonogashira cross‐coupling polycondensation reaction in the continuous phase of the HIPEs, were used as the heterogeneous photocatalyst for the degradation of BPA. The optimal alignment of the valence and conduction band levels of these PAE‐based polyHIPEs was effective for their photocatalytic performance. The strong light‐harvesting ability of such porous foams in the visible‐light region was mainly attributed to the *π*‐extended PAE main backbone with donor–acceptor characteristics of finely tuned optical bandgaps (1.70 to 2.35 eV).^[^ ^127^ ^]^

### 3.3 Gas Capture/Air Treatment

Air pollution resulting from industrial production and transportation is the severest environmental concern of the day. The anthropogenic CO_2_ emissions are increasing global warming, whereas the prolonged stay of the harmful particulate matter in atmosphere is causing respiratory disease, readily triggering allergy, and even cancer.^[^ ^289^ ^]^ PolyHIPE materials, usually with amine groups or other basic groups, have been widely used for CO_2_ capture. P(St‐*co*‐DVB) polyHIPEs impregnated with PEI exhibited good performance in capturing CO_2_ with adsorption capacity of 5.6, 4.5, and 6.4 mmol g^−1^ for pure CO_2_, 10% CO_2_/N_2_, and under moisture, respectively. Noteworthy, this adsorbent showed outstanding CO_2_/N_2_ selectivity, good adsorption/desorption kinetics, and promising stability during the capture and release cycles.^[^ ^108^ ^]^ Porous P(VBC‐DVB) was functionalized with various diamines for CO_2_ capture. The adsorption capacity was affected by the type of amine and the loading of amine groups within the porous P(VBC‐DVB). Highest CO_2_ uptake was achieved with EDA‐functionalized P(VBC‐DVB).^[^ ^124^ ^]^

The P(St‐*co*‐DVB)/nano‐TiO_2_/PEI‐50 composites could capture CO_2_ with an uptake capacity of 5.25 mmol g^−1^. The high adsorption/desorption rates, long‐term stability over a wide range of temperatures, high stress–strain strength, and thermal conductivity made this material highly useful for potential large‐scale industrial applications.^[^ ^199^ ^]^ Porous HKUST‐1‐PGD polyHIPEs were further functionalized by PEI via epoxy‐amine reaction and amine‐metal complexation. These composites offered high surface area and amine groups, resulting in high capacity of CO_2_ adsorption, that is, 4.3, 3.0, and 1.8 mmol of CO_2_ g^−1^ adsorbent from pure CO_2_, simulated flue gas, and air, respectively. Highly efficient separation of CO_2_/N_2_, with a separation factor of 76, was also demonstrated. These adsorbents were highly reusable, with the adsorption capacity higher than 2.8 mmol of CO_2_ (from simulated flue gas) per gram after 20 cycles.^[^ ^217^ ^]^

PEI polyHIPEs doped with 1.5 wt% acidified CNTs could capture CO_2_ by “breathe‐in” at dark and release CO_2_ by “breathe‐out” via NIR light irradiation. The photothermal conversion ability of CNTs‐doped black foam regulates the CO_2_ absorption and desorption (Figure 19c,d).^[^ ^288^ ^]^ The CO_2_ capture ability of this porous material was 2.555 wt % using 25 mg cm^−3^ PEI. The reversible CO_2_ capture and release could be carried out for several cycles. Moreover, this breathing foam showed the capability to separate CO_2_ mixed with CH_4_, which is highly beneficial for CO_2_ enrichment and practical methane purification.^[^ ^288^ ^]^

PolyHIPEs containing quaternary ammonium ions and hydroxide counter ions have been found very useful for reversible CO_2_ capture by humidity swing. Under dry condition, CO_2_ is captured because the formation of bicarbonates is favored. However, under humid condition, CO_2_ is released and bicarbonates are changed to carbonates. The adsorption rate and swing size are two important parameters for this technology to be practically useful.^[^ ^290^ ^]^ A polyHIPE was formed by copolymerization of *p*‐chloromethylstyrene and DVB and subsequent quaternization with trimethylamine and ion exchange. This material showed a very fast adsorption rate (1.1 × 10^−1^ mmol g^−1^ min^−1^), moderate desorption rate (3.3 × 10^−2^ mmol g^−1^ min^−1^), and an excellent swing size (4.9 × 10^−1^ mmol g^−1^) for CO_2_ capture from ambient air, much better than the commercially available materials (Excellion membrane and Excellion active resin) with similar functional groups.^[^ ^186^ ^]^ The P(VBC‐DVB) polyHIPEs were modified by trimethylamine and then undergone ion exchange with KOH to obtain quaternary ammonium hydroxide groups. The performance of these functional polyHIPE materials for reversible CO_2_ capture was compared to that of the commercially available Excellion membrane bearing similar chemical moieties.^[^ ^291^ ^]^ The Excellion membrane gave rise to an overall rate of 2.1 × 10^−3^ mmol min^−1^ g^−1^ and a swing size of 1.3 × 10^−1^ mmol g^−1^. However, the best performing polyHIPE showed a swing size of 7.2 × 10^−1^ mmol g^−1^ with an overall rate of 1.6 × 10^−2^ mmol min^−1^ g^−1^.^[^ ^291^ ^]^

Hierarchically porous zeolite@carboHIPE foams were used to selectively capture CO_2_. The ultramicropores from zeolite combined with highly interconnected macro‐ and mesopores from the carbon matrix helped to achieve a CO_2_ uptake capacity of 5 mmol g^−1^, CO_2_/N_2_ selectivity of up to 80, and recyclability under humid conditions (50% humidity, 70% performance retention after 30 regeneration cycles).^[^ ^250^ ^]^ This adsorbent was regenerated via an electric swing adsorption method. It was therefore essential to utilize the electrically conductive carbon matrix (2.2 S m^−1^) to generate efficient Joule‐heating under the applied voltages. The cooling process was also fast with the carbon matrix. This enabled a fast and energy‐efficient regeneration of zeolite@carboHIPEs as adsorbents.^[^ ^250^ ^]^

Silica‐P(St‐*co*‐DVB) polyHIPEs were fabricated by using the acidified cotton as a stabilizer. These polyHIPEs offered good mechanical stability, high porosity, high diffusivity, and strong affinity for the particulate matter. The silica‐P(St‐*co*‐DVB) was employed to remove particulate matters (smoke from burning cigarette as model particles) with a removal efficiency of 93% for PM2.5 (particulate matter with sizes less than 2.5 µm) and saturated adsorption capacity of ≈520 mg g^−1^ at ambient conditions. These monoliths were easily separated, recycled, and cost‐effective to be used for the real‐life practical applications (Figure 19e,f).^[^ ^187^ ^]^ HIPE‐templated porous amino‐functionalized P(St‐*co*‐MMA) monolith filters showed promise in air purification to capture CO_2_ and PM_2.5_ with 73% and 77.2% efficiencies, respectively. Such porous monoliths also demonstrated excellent dust loading capacity with a reduced pressure drop and reusability as an air filter on continuous usage.^[^ ^118^ ^]^

### 3.4 Sensing

The monitoring of heavy metals, food color, aldehydes, biochemical and biohazard‐related compounds, environmental nutrients, toxins, and toxic gases via sensing is crucial especially for environmental applications. The sensors identify, respond, and subsequently exchange chemical, biological, and physical environmental changes into analytical/electrical signals. On the basis of type of analyte and change in environment, sensors can be categorized as ion sensors, strain sensors, bacterium sensors, temperature sensors, humidity sensors, gas sensors, etc. They play key roles in areas such as pollutant detection, gas alarms, human health monitoring, and so forth.^[^ ^292^ ^]^ It helps to take necessary precautionary measures to keep the levels of such hazardous substances below permissible limits. Many types of materials such as NPs, nanotubes, nanowires, nanosheets, and nanobelts have widely been used as promising sensors. But, the development of emulsion‐templated porous materials is receiving more attention nowadays. A few reviews summarizing the progress in the sensing area have recently been reported.

Functionalized polyHIPEs have been used for sensing applications. A zwitterionic hydrogel‐based polyHIPEs showed rapid swelling in aqueous medium. The water uptake was affected by pH due to the ionic groups presented on the polyHIPEs. This could be used to sense the surrounding aqueous environment.^[^ ^145^ ^]^ It was found that the combined effects from the zwitterionic charges and the polyHIPE structure amplified the anti‐polyelectrolyte effect. The uptake of NaCl solution was increased upon increasing NaCl concentration. Unusually, the uptake of urine was twofold higher than the water uptake. This technique provided insights for the design and synthesis of new materials for the amplification, acceleration, and sensitization of the environmental variations by emulsion‐templated porous materials.^[^ ^145^ ^]^

The ionophores, graphite particles, electron mediators, and enzymes‐impregnated P(St‐DVB‐EHA) membranes were reported for their superior capability in electrochemical sensing. Among the prepared materials, a valinomycin ionophore‐incorporated plasticized membrane exhibited a Nernstian response to K^+^ ions with faster response time, enhanced limits of detection, and an improved selectivity coefficient better than PVC membranes. This was attributed to the pore morphology, decreased impedance, and enhanced transport to the membrane backbone. Furthermore, the asymmetric composite membrane fabricated from a polyHIPE layer and PVC layer showed the most improved performance for the detection of K^+^ ions.^[^ ^121^ ^]^

Hierarchically porous Co_3_O_4_ material was prepared using assembled PS microspheres as templates.^[^ ^293^ ^]^ Although it was not fabricated by the emulsion templating method, this material exhibited a porous structure that resembled the emulsion‐templated structure. Porous Co_3_O_4_ was evaluated for sensing NH_3_ gas based on the increase in resistance resulting from the interaction of a p‐type semiconductor and NH_3_ gas. The electrode coated with Co_3_O_4_ was exposed to NH_3_ in a gas chamber at a humidity of 26% and 21 °C. NH_3_ gas sensing with high sensitivity (146%, 100 ppm NH_3_), quick response time (2 s, 100 ppm NH_3_), and low limit of detection (0.5 ppm) was achieved. This high performance in NH_3_ sensing was attributed to the hierarchically porous structure, high surface area, gas sorption behavior, and catalytic nature of Co_3_O_4_.^[^ ^293^ ^]^

A hierarchically porous composite of rGO/polyHIPE was evaluated as a piezoresistive pressure sensor, which showed high sensitivity over a pressure range of 0.6 Pa–200 kPa with very fast response times (less than 15.4 ms). Excellent cyclic stability of this sensor was demonstrated with 10 000 cycles or more. The hierarchical porosity was suggested to improve deformation homogeneity, enhance the construction of new conductive pathways, and extend pressure detection thresholds.^[^ ^253^ ^]^ Yang et al. fabricated rGO/polyHIPE composites from multiple monomers (including St, DVB, *n*‐butyl acrylate, and 2‐ethylhexyl methacrylate) and assessed their performance as piezoresistive sensor. These composite foams exhibited elastomeric behavior, shape‐retaining capability, and improved electrical conductivity. The high sensitivity in response to compressive strain was reflected by the gauge factor value of 1.5 in the strain range of less than 15%. The pressure response range was found to be more than 200 KPa. A highest pressure sensitivity of 0.83 kPa^−1^ was observed for pressure <20 kPa.^[^ ^255^ ^]^

### 3.5 Summary

Section 3 is specifically devoted to review the existing literature on environmental applications of emulsion‐templated porous materials. To sum up, significant progress has been made to prepare emulsion‐templated porous polymers, organic–inorganic hybrids/composites, inorganic structures, and carbonaceous/graphene‐based materials for solving some pressing environmental problems. Such emulsion‐templated porous nanomaterials have been reported for the removal and sensing of water and air/gas contaminants via adsorption, separation, degradation, oxidation mechanism, etc. In terms of performance, the most influential findings manifested promising adsorption, separation, and disinfection potential of the emulsion‐templated porous materials for Pb (613 mg g^−1^), MB (1.66 g g^−1^), chloroform (81.30 g g^−1^), oil (600 g g^−1^), and *E*. *coli* (100%) (see (Table 5). This outstanding performance pertaining to the enhanced convective mass transport through macropores, improved surface area on account of the meso‐/micropores and nanodimensions, higher overall charge density, diverse anchoring sites, electrostatic/ionic interactions, and coordination/chelation/complexation properties that makes the emulsion‐templated porous materials highly competitive and even superior to many state‐of‐the‐art conventional practically worthwhile materials being used for the same purpose. However, the endowment of appropriate functional groups and chemistry to these emulsion‐templated porous materials is highly indispensable for high specificity of interaction with targeted pollutants. The reported materials can also be recycled 5–20 times on average and 50 times in some cases. Given the macrosize of the emulsion‐templated porous materials, these are highly valuable for industrial adoption. This review presented many examples of high‐impact research, though there is still a wider scope to develop new paradigms in emulsion templating for environmental remediation to emerge. On the other hand, the concern of the community has moved toward the promotion of industrial‐scale utilization of these materials. In this context, the scaling‐up, long‐term robustness, and durability of these emulsion‐templated porous materials to sustain their functions as well as critical structure after multiple cycles in realistic condition is still challenging. This indeed needs further research on the preparation of high performance, scalable, stable, durable, recyclable, and safely disposable emulsion‐templated porous materials and their subsequent assessment on their stability in acidic/basic environments, complicated organic solvent systems, and at high temperatures before their commercialization for community‐scale applications. It is therefore imperative for the researchers, engineers, visionaries, and industrialists to accelerate their best collaborative efforts to find compelling new solutions for real life applicability of the emulsion‐templated porous materials for materializing a dream of practically viable, environment‐friendly, and cost‐effective point‐of‐use water treatment technology.

## 4 Conclusions and Perspectives

Environmental pollution is the gravest concern of the day and the life symbols (i.e., water and air) are uninterruptedly being contaminated largely by the rapid industrialization and human activities, and thus the sustainable provision of safe and clean water and air has become a universal challenge. A wide range of materials and technologies have been developed in monitoring key trace contaminants and enabling their subsequent remediation. Among various materials developed for environmental remediation, porous materials including porous polymers, porous organic cages, supramolecular organic frameworks, zeolites, porous carbon, mesoporous silica, and metal/covalent/hydrogen‐bonded‐organic frameworks have recently received much scientific attention for their potential breakthroughs in environmental applications, but most of these materials possess micropores and mesopores of less than 10 nm with limited mass transfer and slow response time. The emulsion templating method has been unique and versatile in generating highly interconnected macroporous materials (with the pore sizes mostly in the region of microns) in the forms of monoliths, beads, and microspheres.

By combining emulsion templating with other templating methods, incorporation of microporous materials, and a rich variety of chemical modifications, it has been exciting to see a wide range of functional and hierarchically porous materials being developed for environmental and other applications. Many environmental researchers have set out to develop novel porous polymers, organic–inorganic hybrids or composites, inorganic structures, and carbonaceous/graphene‐based materials via greener, cheaper, easily reproducible, and scalable approaches. These state‐of‐the‐art porous emulsion‐templated materials have shown great promise for environmental applications via adsorption, affinity, and catalytic mechanisms.

In addition to the environmental applications described above, emulsion‐templated porous materials have also been widely used in other applications such as heterogeneous catalysis,^[^ ^132^, ^152^, ^203^, ^211^, ^220^, ^226^, ^249^ ^]^ biomedical applications,^[^ ^26^, ^50^, ^161^, ^170^ ^]^ energy storage,^[^ ^234^, ^236^, ^241^, ^251^, ^258^ ^]^ and chromatography,^[^ ^111^, ^123^ ^]^ to name a few. All these applications have explored the highly interconnected hierarchical porosity, chemical functionality and desired physical properties presented by emulsion‐templated porous materials. Here, we summarize and propose some design concepts or criteria in order to develop novel advanced porous materials for target applications.

1) A careful choice of suitable monomers to produce hierarchically porous materials with desired hydrophobicity/hydrophilicity, acidity/basicity, negative/positive charges, and the potential for subsequent modification. For example, AA and AMPS are widely used for acidity and negative charges, DEAEMA for positive charges, and benzyl chloride and epoxy groups for quaternization. Importantly, the use of sustainable monomers such as FA and tannin offers additional green credentials. An exciting development is to hypercrosslinked St/DVB/VBC‐based polyHIPEs for porous polymers with high surface area and hierarchical porosity.
2) Suitable selection of surfactants and stabilizers. These include charged surfactants, non‐ionic polymeric surfactants, reactive surfactants, and NPs based stabilizers for Pickering emulsions. First, the selected surfactants should be able to form stable emulsions or double emulsions. Co‐stabilizers (e.g., PVA or a combination of inorganic NPs and polymer stabilizers are often used. The use of NPs or nanosheets as stabilizers are becoming attractive choice to prepare emulsion‐templated composites with improved mechanical/thermal stability and other properties. Generally, the use of metal oxide and clay nanoparticles and CNTs/GO can improve thermal/mechanical stability and electrical conductivity. Using Fe_3_O_4_ and TiO_2_ NPs as stabilizers, magnetic and photocatalytic properties can be additionally induced to the porous composites. The same may be explored for other types of metal oxides or inorganic materials in general. Recently, the development and use of microporous polymer NPs and MOF NPs provide a new platform for Pickering stabilizers, thereby offering microporosity and the rich surface chemistry in the emulsion‐templated materials, but it should be noted that although the MOF and MOP exhibit very high surface area and designed chemical functionality, the micropores may be blocked during the polymerization.
3) Incorporating functional components into the emulsion‐templated porous materials. This can be achieved during the stage of polymerizing the emulsion or via post‐treatment. By adding desired components into the internal phase, external phase, or both phases of the emulsion, functional composite materials can be produced by one‐step polymerization. However, the emulsions may be destabilized when additional components are added and hence the quality of the emulsion‐templated porous structures can be destroyed or impacted. This disadvantage may be addressed by employing the post‐modification approach. The functional components may be chemically bonded to the polymer matrix and/or physically attached to the pore wall or entrapped within the pores. Furthermore, the property of the functional component added may be retained. For example, the high surface area and other properties of MOFs can be readily maintained in the MOF/polymer composites prepared by the post‐modification method.
4) Tuning the porosity and relevant properties by altering the emulsion formulation and combining the emulsion templating with other templating methods. The internal phase volume percentage and the concentration of monomers/additives can be rationally varied to tune the pore volume, pore size, and pore interconnectivity which will in turn influence mechanical stability, diffusivity, and response sensitivity. This concept can be further enhanced by using emulsion templating and other templating, for example, in conjunction with sol–gel process,^[^ ^190^ ^]^ freeze‐drying/ice templating,^[^ ^82^, ^158^, ^259^ ^]^ molecularly imprinting approach,^[^ ^154^ ^]^ etc.
5) Fabrication of hierarchically porous carbon and composite materials. A high percentage of the applications require high surface area, interconnected porosity, and enhanced mass transport via large pores. It is not common to combine all these properties in one material. For examples, microporous and mesoporous materials, for example, zeolite, silica, active carbon, and MOFs, offer high surface area but lack interconnected macropores while most of the emulsion‐templated porous polymeric materials (although there are exceptions, e.g., hypercrosslinked polyHIPEs, porogen‐templated polyHIPEs) exhibit low surface area (usually <100 m^2^ g^−1^). However, emulsion‐templated porous carbon, prepared by carbonization of polyHIPEs, meets all these requirements. The porous structures and carbonization yield can be enhanced by the incorporation of inorganic or carbon nanomaterials. There are a few aspects that need to be highlighted in this category: i) carbon‐rich polymer polyHIPEs can be prepared by polycondensation reactions with various monomers/macromers including the ones from renewable sources; ii) choosing suitable monomers or inclusion of additional components (e.g., metal NPs) for N, S, P‐doped advanced carbon materials; iii) the use of CNTs and graphene nanosheets, either as building blocks or additional components, for improving elasticity, conductivity, stability, etc.; iv) adopting carbon activating approaches (both chemically and physically) to achieve high surface area and high microporosity; v) utilizing crystalline porous materials (e.g., MOFs, covalent organic frameworks, organic cages) as novel precursors for carbon materials with controlled nanopores to achieve properties superior to original precursor materials (e.g., conductivity, catalytic property, etc.) can be achieved. It is generally very difficult to control pore size and pore morphology when carbonizing conventional polymer precursors.
6) Generating interconnected porous metals, metal oxides, and composite materials. In this regard 03 possible routes may be applied: i) directly use their NPs (and maybe with co‐stabilizers) to form emulsions, which are then frozen and freeze‐dried to produce the target porous materials. However, their mechanical stability can be very weak. Thermal treatment is recommended to fuse the NPs while maintaining the emulsion‐templated porous structures. This may be only applied to metals or metal oxides with low melting points. Porous metals can be prepared from porous metal oxides by reduction, for example, with H_2_/Ar under high temperature; ii) incorporating metal precursors into the emulsion to prepare metal‐ or metal oxide‐polymer composites. A calcination process is then applied to fabricate the relevant porous inorganic materials; iii) impregnating metal oxide precursor sols into the pre‐formed polymeric polyHIPEs. It is important to control the loading so that the pore walls are coated, the windows are not blocked, and pore structure is not collapsed after calcination. Various templating methods that are used to prepare silica or metal oxides, for example, surfactant assembly, colloids templating, may be still included in the precursor sols in order to prepare hierarchically porous metal oxides.

From an environmental application perspective, polyHIPEs are highly porous and permeable materials wherein the large number of pores play a substantial role to increase their performance in environmental applications, but the unbound/unreacted ingredients/components of HIPE recipe and sometimes the nano/microparticles of the polyHIPE‐based composite/hybrids are known to be leachable and may cause secondary contamination. Likewise, the practical viability of polyHIPEs suffers from poor mechanical robustness ascribed to their relatively lower density that in turn impedes their application in point‐of‐use technology development. Among various strategies including the addition of organic or inorganic charges and the use of different types and doses of monomers to circumvent this conundrum, the more attractive method is to use MIPEs or LIPEs. This approach may considerably improve mechanical properties of the emulsion‐templated porous materials, though the relatively higher density (wall thickness) may result in the loss of highly interconnected open‐cell structure typically found in polyHIPEs. Also, the shape and structure of the pores in emulsion‐templated porous materials may undergo deformation after drying step of the nanoparticle formation inside the porous polymers and/or after the regeneration process for their next‐cycle metal/dye ion/molecule remediation, air/gas filtration, and oil spill clean‐up applications.

The future development of emulsion‐templated porous materials is most likely to be application based, that is, first decide the functions and properties required for the target applications and then fabricate the materials that meet the requirements. For environmental applications, the emulsion‐templated porous materials will continue to offer various benefits and more specifically to address the low performance in cost‐effective terms, secondary contamination, and energy‐intensive regenerating/recycling processes. These are believed to be among the major challenges in advancing practically worthwhile water treatment technologies. With the design concepts proposed above and intensive efforts by the researchers from multidisciplinary and interdisciplinary subjects, advanced functional emulsion‐templated porous materials will more likely continue to be developed for environment applications and other relevant applications, with high performance, low cost, and decent potential for upscaling.

## Conflict of Interest

The authors declare no conflict of interest.
